# Accurate prediction of gain- and loss-of-function missense variants in GABA_A_ receptors

**DOI:** 10.1016/j.ebiom.2026.106417

**Published:** 2026-08-03

**Authors:** Christian M. Boßelmann, Sebastian Ortiz, Rebekka Dahl, Vivian W.Y. Liao, Serene El-Kamand, Susan X.N. Lin, Anthony Sze Hon Kan, Tobias Brünger, Dennis Lal, Holger Lerche, Jules Kreuer, Nico Pfeifer, Mary Chebib, Nathan L. Absalom, Philip K. Ahring, Rikke S. Møller

**Affiliations:** a Department for Neurology and Epileptology, Member of ERN EpiCARE, Hertie Institute for Clinical Brain Research and Hertie Center of Neurology, University Hospital Tübingen, Tübingen, 72076, Germany; b Department of Epilepsy Genetics and Personalized Medicine, Member of ERN EpiCARE, Danish Epilepsy Centre, Dianalund, 4293, Denmark; c Department of Regional Health Research, Faculty of Health Sciences, University of Southern Denmark, Odense, 5230, Denmark; d Department of Neurology, McGovern Medical School, The University of Texas Health Science Center at Houston, Houston, TX, 77030, USA; e School of Medical Sciences, Faculty of Medicine and Health, Brain and Mind Centre, The University of Sydney, Sydney, NSW, 2050, Australia; f Department of Neuroscience, University of Copenhagen, Copenhagen, 2200, Denmark; g Epilepsy Center, Neurological Institute, Cleveland Clinic, Cleveland, OH, 44195, USA; h Program in Medical and Population Genetics, Broad Institute of Massachusetts Institute of Technology (M.I.T.) and Harvard, Cambridge, MA, 02142, USA; i Stanley Center for Psychiatric Research, Broad Institute of Harvard and M.I.T, Cambridge, MA, 02142, USA; j Department of Computer Science, Methods in Medical Informatics, University of Tuebingen, Tuebingen, 72076, Germany; k School of Science, Western Sydney University, Penrith, NSW, 2750, Australia

**Keywords:** Ion channel, Electrophysiology, Machine learning, Epilepsy, Genetics, Neurodevelopmental disorders

## Abstract

**Background:**

Missense variants in genes encoding GABA_A_ receptors are involved in the pathophysiology of common and rare epilepsies. Variant effects on channel biophysical function are associated with key clinical characteristics and treatment response. Predicting variant effects is therefore key to improving care for individuals with GABA_A_ receptor-related disorders.

**Methods:**

We collected data from 505 affected individuals with 272 (likely) pathogenic GABA_A_ receptor missense variants (*GABRA1, GABRB2, GABRB3, GABRG2*). All variants were evaluated with *in-vitro* electrophysiology. Variants were annotated with features based on sequence, structure, and phenotype. Model performance was estimated using cross-validation and external validation on a further 197 individuals with 138 (likely) pathogenic variants.

**Findings:**

Our models enable highly accurate prediction of missense variant effects in GABA_A_ (AU-ROC 0.862–0.946), outperforming state-of-the-art models (AU-ROC 0.495–0.756) and clinical decision-making. Model scores correlated with GABA sensitivity and were consistent with expert-based structure–function hypotheses, supporting plausibility. Predictions on population variants were similar to functionally neutral variants, while cases from ClinVar were similar to GOF/LOF variants. Our model may provide additional evidence for 5–25% of variants in ClinVar. Lastly, we show that we can predict likely clinical characteristics from variant information alone (median Lin similarity 0.754 IQR 0.161).

**Interpretation:**

We demonstrate accurate missense variant effect prediction in GABA_A_ receptors with rigorous validation across a large dataset of functionally tested variants. These predictions may facilitate timely diagnosis and precision treatment of individuals with GABA_A_ receptor-related disorders, pending prospective clinical validation. A web interface, precomputed scores, and calibrated score thresholds for all possible variants are openly available.

**Funding:**

Else Kröner-Fresenius-Stiftung; German Federal Ministry of Research, Technology and Space; German Research Foundation; Medical Faculty University of Tübingen; Lundbeck Foundation; Novo Nordisk Foundation.

**Research in context**

**Evidence before this study**

While genome-wide computational tools for predicting variant pathogenicity exist (e.g., REVEL, AlphaMissense), and one recent tool (LoGoFunc) attempts functional effect (i.e., gain- or loss-of-function) prediction, no gene family-specific functional prediction model has been developed specifically for GABA_A_ receptors. Previous studies on voltage-gated ion channels have demonstrated the feasibility of variant functional effect prediction but were limited by small or heterogeneous datasets and lacked comprehensive validation.

**Added value of this study**

Variants in GABA_A_ receptors have recently been demonstrated to lead to either gain- or loss-of-function, where either variant effect has immediate clinical consequences, including choice of medication (e.g., avoiding vigabatrin in gain-of-function disease) and prognosis. This study extends previous research to enable accurate prediction of variant mechanisms in GABA_A_ receptors by training machine learning models on a large curated dataset of functionally characterised GABA_A_ receptor variants, comprising 505 individuals with 272 variants. Model predictions were generated for all possible missense variants, comprehensively benchmarked against other models, aligned with structure-function relationships, applied across a large case-control cohort of individuals with epilepsy, and calibrated to evidence thresholds to facilitate use.

**Implications of all the available evidence**

This work addresses a key translational gap in precision medicine for GABA_A_ receptor-related disorders. Pending prospective clinical validation, the accurate prediction of variant functional effects may help facilitate timely diagnosis, inform genetic counselling, and enable patient identification and stratification for emerging precision therapies.

## Introduction

Variants in genes encoding for GABA type A (GABA_A_) receptor subunits are associated with both common and rare epilepsies, ranging from risk factors for febrile seizures or genetic generalised epilepsy in otherwise healthy individuals to severe developmental and epileptic encephalopathies.^1^, ^2^, ^3^, ^4^, ^5^, ^6^, ^7^ GABA_A_ receptors are ligand-gated, primarily postsynaptic anion channels that mediate inhibitory neurotransmission and thus play a central role in the homoeostasis of the excitatory-inhibitory balance.^8^^,^^9^ In total, 19 genes encode the GABA_A_ receptor subunits: *GABRA1-6* (α1-6), *GABRB1-3* (β1-3), *GABRG1-3* (γ1-3), *GABRD* (δ), *GABRE* (ϵ), *GABRP* (π), *GABRQ* (θ), and *GABRR1-3* (ρ1-3). Out of these, the following have so far repeatedly been associated with Mendelian disease: *GABRA1, GABRA2, GABRA3, GABRA4, GABRA5, GABRB1, GABRB2, GABRB3, GABRG2, GABRD*.^1^^,^^6^^,^^8^^,^^10^, ^11^, ^12^, ^13^, ^14^, ^15^, ^16^, ^17^, ^18^, ^19^, ^20^ A complete overview of gene-disease associations is provided in Supplementary Table S1, with the understanding that the phenotypic spectra and penetrance estimates for each disorder are subject to ongoing research.

Previously, GABA_A_ receptor variants associated with epilepsy were thought to only lead to loss of receptor function (LOF, e.g., reduced GABA sensitivity and/or decreased GABA-evoked current amplitudes), in turn resulting in impaired inhibitory neurotransmission, neuronal network hyperexcitability, and thus seizures. This assumption was challenged by recent research that demonstrated gain-of-function effects (GOF, primarily observed as increased GABA sensitivity) in variants associated with vigabatrin hypersensitivity and distinct neurodevelopmental phenotypes.^1^^,^^13^, ^14^, ^15^, ^16^^,^^18^ Thus, knowledge of variant functional effect (GOF/LOF) has become key information for translational care in these often severely affected individuals. In related ion channel and transporter disorders, variant functional effect can facilitate diagnosis, inform genetic counselling, enable candidate precision therapies, and thus improve prognosis.^21^, ^22^, ^23^

Direct experimental evidence from *in-vitro* electrophysiology remains the gold standard to ascertain variant functional effect. However, this traditional approach represents a potential challenge to translational care as electrophysiology requires considerable time, experience and resources and thus is not available for routine clinical care. With the increasing availability of next-generation sequencing, including population-wide newborn screening programmes, an increasing number of individuals are affected by variants of uncertain significance and unknown functional effect.^24^, ^25^, ^26^ The resulting uncertainty delays a timely diagnosis and adds to the substantial clinical, psychosocial, and emotional burden of the ‘diagnostic odyssey’.^27^^,^^28^ This translational gap may be addressed by machine learning algorithms that predict variant effect, which has been successfully demonstrated for voltage-gated ion channels (Na_V_, K_V_, Ca_V_) and NMDA receptors.^29^, ^30^, ^31^, ^32^, ^33^

Three insights now enable the development of such a predictive algorithm specifically for GABA_A_ receptor subunits. First, multi-task learning can make efficient use of gene family information to learn from paralogous variants.^32^^,^^34^, ^35^, ^36^ Second, phenotypic learning integrates harmonised clinical data and leverages known and implicit genotype-phenotype correlations.^13^^,^^14^^,^^33^ Third, variant distance from pore axis and allosteric pathways separates functional subgroups.^31^^,^^35^^,^^37^ Here, we integrate these concurrent developments to train our model on a large clinical and experimental dataset. The resulting model, GABA_A_ receptor functional variant effect prediction using multi-task phenotypic learning (GENTLY), outperforms state-of-the-art genome wide predictors and clinical decision-making. We demonstrate biological plausibility by correlating model scores with GABA sensitivity and conforming to expert-based structure–function hypotheses. Lastly, our model has utility for large cohorts by separating case from population variants and allowing for inference of likely clinical characteristics.

## Methods

### Clinical data collection

Data were collected between 1 June 2023 and 1 October 2025. Individuals carrying (likely) pathogenic variants, classified according to ACMG criteria,^38^ in genes encoding GABA_A_ receptor subunits were recruited through an international network of collaborating paediatric and adult neurologists and medical geneticists including the European Reference Network for Rare and Complex Epilepsies (ERN-EpiCARE). Additionally, individuals were recruited via the international patient advocacy group CURE GABA-A Variants. Independent from referral mode, all individuals were reassessed either virtually or in-person at the Danish Epilepsy Centre in Dianalund. Variant classification as (likely) pathogenic was determined by the submitter and their diagnostic laboratory. Additionally, we conducted automated ACMG classification for each variant (Supplementary Methods, Supplementary Table S2). No power analysis was used, and sample size was determined by availability under a prospective registry-based study design.

Electronic data capture was standardised with case report forms via REDCap (Research Electronic Data Capture) hosted at the Danish Epilepsy Centre in Dianalund.^39^ Clinical data included age at seizure onset and were further harmonised using the human phenotype ontology (HPO), 2024-12-12 Release.^40^ Data collection and experimental characterisation first focused on four genes (*GABRA1, GABRB2, GABRB3, GABRG2*) which therefore constitute the training dataset. As data on additional variants in other genes became available these were retained as an independent prospective test set.

### Functional experiments

Functional evaluation of variant receptors was performed using a custom-made two-electrode voltage clamp apparatus described previously.^1^^,^^14^^,^^18^ Briefly, concatenated pentameric receptor constructs using human GABA_A_ receptor subunits were used that allowed for the systematic introduction of a point mutated subunit into the first, second, third or fourth position of a γ2-β2-α1-β2-α1 or γ2-β3-α1-β3-α1 construct. Mutant constructs were made and verified by sequencing followed by sub-cloning into the concatenated construct using standard restriction digestion and ligation. Linearised cDNA was generated and cRNA for each concatenated receptor construct was produced using the mMessage mMachine T7 Transcription Kit (Thermo Fisher, #AM1344).

The cRNAs of wildtype and mutant concatenated receptors were injected into oocytes at ∼25 ng cRNA per oocyte. Oocytes were incubated for 2 days at 18 ^°^C in modified Barth's solution (96 mM NaCl, 2 mM KCl, 1 mM MgCl_2_, 1.8 mM CaCl_2_, 5 mM HEPES, 2.5 mM sodium pyruvate, 0.5 mM theophylline, and 100 mg/L gentamicin; pH 7.4). All recordings were performed at room temperature. Oocytes were placed in a recording chamber, and ND96 solution (96 mM NaCl, 2 mM KCl, 1 mM MgCl_2_, 5 mM HEPES, 1.8 mM CaCl_2_, pH 7.4) was continuously perfused. The pipettes were backfilled with 3 M KCl and had open pipette resistances from 0.4 to 2 MΩ when submerged in OR2 solution. Oocytes were voltage clamped using an Axon GeneClamp 500B amplifier (Molecular Devices) at a holding potential of −60 mV. Amplified currents were low-pass filtered at 20 Hz using a four-pole Bessel filter (Axon GeneClamp 500B), digitised using a Digidata 1440A (Molecular Devices; RRID:SCR_021038) and sampled at 200 Hz on a personal computer using the pClamp 10.2 suite (Molecular Devices; RRID:SCR_011323). Episodic traces following triggering events representing responses to individual applications were collected.

On each experimental day, the functional properties of wildtype receptors were assessed along with the mutant receptors to eliminate the impact of inter-day variation and variation between batches of oocytes. To assess maximum current amplitudes, 10 mM GABA was applied. GABA concentration-response relationships were then determined by applications of increasing concentrations of GABA to the oocyte. Final datasets for GABA concentration-response were collected from at least 10 independent experiments performed on at least two different batches of oocytes.

Raw traces were analysed using pClamp 10.2. To determine the EC_50_ values of GABA concentration-response relationships, the Hill equation was fitted to peak GABA-evoked current amplitudes for individual oocytes using GraphPad Prism 10:

$$I={Abs.I}_{\max}\left({\left[A\right]}^{nH}/\left({\left[A\right]}^{nH}+{\left[{EC}_{50}\right]}^{nH}\right)\right)$$

Where $\text{Abs}.I_{\max}$ is the absolute maximum current, ${\text{EC}}_{50}$ is the concentration that evoke half-maximum response, [A] is the ligand (GABA) concentration and nH is the Hill slope. For each individual oocyte, a complete concentration-response curve was recorded as a single determination (n). From the ${\text{EC}}_{50}$ value the corresponding $\log\;{\text{EC}}_{50}$ value was calculated. By fitting the Hill equation to all data for each construct, final ${\text{EC}}_{50}$ values were calculated. For each experimental day the mean $\log\;{\text{EC}}_{50}$ for wildtype construct ($\log\;{\text{EC}}_{50,\text{wt}}$) was calculated. In addition, the $\Delta \log\;{\text{EC}}_{50}$ value for each oocyte containing a mutant construct tested on the same day was calculated using the following equation:

$$\Delta \log\;{EC}_{50}=\log\;{EC}_{50,wt}-\log\;{EC}_{50}$$

Variants were defined as GOF if the ΔlogEC_50_ was greater than 0.2 and *p* < 0.0001, and LOF where the ΔlogEC_50_ was less than 0.2 and *p* < 0.0001 when compared by one-way ANOVA with Dunnett’s post-hoc test under a known lognormal distribution.

The normalised maximum GABA-evoked current amplitude ($I_{\max}$) was calculated using the peak current evoked by 10 mM GABA at wildtype controls (${\text{Abs}.I}_{\max,\text{wt}}$) and mutants $\left({\text{Abs}.I}_{\max}\right)$ for parallel experiments performed on the same experimental day. To determine the $\left(I_{\max}\right)$ for each individual experiment on a variant, the following equation was used:

$$I_{\max}=\frac{Abs.I_{\max}}{{Abs.I}_{\max,wt}}$$

Variants were defined as LOF based on the Imax where the reduction was greater than 50% and *p* < 0.0001 when compared with a Mann–Whitney test to wild-type under a known non-normal distribution. We did not measure surface expression and did not identify mixed (GOF and LOF) variant effects within our cohort under the previous observation that in cases with increased GABA sensitivity, reduced expression or even changes of ∼70% loss of current still result in clinical phenotypes consistent with GOF.^14^^,^^16^ Thus, each variant had three labels: *(i)* the categorical label of variant effect on channel biophysical function classified as binary gain- or loss-of-function (GOF/LOF); *(ii)* the change in GABA sensitivity, ΔpEC_50_; *(iii)* normalised maximum GABA-evoked current amplitudes.

### Variant annotation

For each gene, variants were described using Matched Annotation from NCBI and EMBL-EBI (MANE) reference transcripts. A list of gene names, transcript IDs, and UniProt accession numbers is provided in Supplementary Table S1. Reference protein sequences were aligned using MUSCLE and used for taxonomy-based multi-task learning as previously described.^41^^,^^42^ Protein-level features based on sequence and structure were calculated as previously described, including domain and motif annotation from UniProt under the accession numbers listed above, binding sites, local disorder, conservation, and residue physicochemical properties.^32^^,^^33^ Protein-level features were transformed into pairwise similarities with the radial basis function (RBF) kernel $k\left(x_{i},x_{i^{\prime }}\right)=\exp-\frac{||x_{i}-x_{i^{\prime }}|{|}^{2}}{2\sigma^{2}}$.^43^

Three-dimensional structural features were calculated using predicted structures from AlphaFold under the UniProt accession numbers listed above.^44^ For each gene, we calculated the Euclidean distance between each pair of residues. Distances were normalised per gene by min-max normalisation. Lastly, distance *d* between each pair of residues *r*_1_ and *r*_2_ was converted to similarity as follows: $\frac{1}{1+d\left(r_{1},r_{2}\right)}$ which resulted in a distance-based similarity matrix.

Clinical features were based on HPO terms for each individual and used to calculate pairwise phenotypic similarity as previously described.^33^ If a variant occurred in more than one individual, the union of sets of HPO terms at the variant level was used. The resulting phenotypic similarity matrix was weighted by the absolute difference in age at seizure onset to allow for better representation of age-dependent phenotypes.

### Model development

The model framework used in this study is a multi-task multi-kernel support-vector machine (MTMKL-SVM), which we have previously developed and found to be accurate and robust in the prediction of variants in voltage-gated ion channels.^32^^,^^33^ The cost regularisation hyperparameter of the support-vector machine was tuned over the range $C=\left\{10^{-6},10^{-4},\ldots ,10^{6}\right\}$. RBF kernel matrices for protein-level sequence and structure features were calculated separately for a range of $\gamma =\frac{1}{2\sigma^{2}}=\left\{10^{-5},10^{-4},10^{-3},10^{-2},10^{-1},1,10\right\}\text{.}$ For taxonomy-based multi-task learning, we tested a range of baseline similarities α={1,2,3,5,10,100}. For multi-kernel learning, we tested sparse and efficient methods (SimpleMKL, SEMKL)^45^, ^46^, ^47^ and uniformly weighted kernels.^48^ For calculating phenotypic similarity between sets of HPO terms, we explored different similarity measures: Jaccard, Lin, Resnik, Euclidean, and cosine.^33^ The optimal values for these hyperparameters were estimated using exhaustive grid search (Table 1).

**Table 1:** Model hyperparameters.

Model	SVM: cost (C)	SVM: gamma (γ)	MTL: baseline similarity (α)	MKL: method	Phenotypic similarity
Full model	16	0.01	100	Uniform	Lin
No clinical features	4	0.01	3	Uniform	–

MKL—multi-kernel learning; MTL—multi-task learning; SVM—support-vector machine.

Model performance was estimated using five repeats of 5-fold cross-validation. Nested cross-validation was prohibitively time-consuming given the large search space defined above and does not generally result in more accurate performance estimates.^49^ Cross-validation splits were done at the variant level to avoid data leakage across individuals, and identical (e.g., recurrent) variants did not appear in both training and test folds. Kernel construction and transformation (scaling, centring) was done on the training splits within folds only to avoid data leakage. No relevant group imbalance was present. Model predictions obtained from *k*-fold cross-validation (distance from decision boundary) were converted into class (GOF/LOF) probabilities using Platt scaling.^50^ We report the following metrics: *(i)* Accuracy, the number of correct classifications divided by the number of all classifications; *(ii)* Balanced Accuracy, the average recall for each class; *(iii)* Sensitivity (true positive rate, TPR); *(iv)* Specificity (true negative rate, TNR); *(v)* Matthews correlation coefficient (MCC), the correlation coefficient between true and predicted classes; *(vi)* F1 score, the harmonic mean of precision and recall; *(vii)* the area under the receiver operator characteristics (AU-ROC) curve; *(viii)* and the area under the precision–recall curve (AU-PRC).

### Benchmark comparisons

We calculated three state-of-the-art genome-wide functional prediction scores for comparison to our own method. Scores were calculated for all possible non-synonymous single-nucleotide variants across all genes encoding GABA_A_ receptor subunits. First, we implemented LoGoFunc, an ensemble learning method trained on 1492 GOF variants from 344 genes and 13,524 LOF variants from 2030 genes where labels were inferred from literature based on a natural language processing pipeline.^51^ Importantly, the genes in their training dataset included *GABRA1, GABRA3, GABRB2, GABRB3, GABRG2*, and *GABRD*.^51^ This would be expected to positively inflate the performance of LoGoFunc on our benchmark. Next, we implemented the 1 billion and 40 billion parameter models of evo2, a large biological foundation model that can be used to assign delta log–likelihood scores for variants compared to reference sequences (Supplementary Methods).^52^ Score calculation required 4 h (1.2 kWh) and 67 h (26.8 kWh) on two NVIDIA H100 Tensor Core GPUs, respectively.

### Population variants

Non-synonymous missense variants in each of the genes encoding GABA_A_ receptor subunits were obtained from two large population databases (gnomAD v4.0 and Regeneron Million Exome Variant Browser; last accessed 12/03/2025) and ClinVar (last accessed 20/11/2025) by querying the gene names listed above while aligning variants to the respective MANE Select reference transcripts.^53^, ^54^, ^55^ Variants that did not pass gnomAD v4.0 quality control filters were discarded.

### Phenotype prediction

Relative variant position within the feature space of the model was visualised using UMAP (Uniform Manifold Approximation and Projection) at default parameters, with multivariate normal distributions calculated from the resulting coordinates.^56^ Enrichment of clinical characteristics in functional classes was calculated using two-sided Fisher’s exact test. Likely phenotypes were estimated using a leave-one-out procedure: For each variant, the k-nearest neighbours in the feature space of the model with no clinical features were found. The set of HPO terms of all *k*-nearest neighbours was then compared to the held-out set of HPO terms of the variant of interest using the Lin similarity measure (range 0–1, with higher values denoting higher phenotypic similarity). For comparison, two baseline methods were implemented: First, the set of HPO terms of *k* random variants that were not *k*-nearest neighbours (“Random other”) was compared to the set of HPO terms of the variant of interest. Second, the elements in the set of “Random other” were instead resampled without replacement from all possible terms in the HPO (“Random HPO”). Both baseline estimates were repeated 10-fold.

### Ethics

The study was approved by the Institutional Review Board of the Danish Epilepsy Centre, Filadelfia (reference number EMN-2024-01998). The study was conducted in accordance with the 2024 Declaration of Helsinki. Written informed consent was obtained from all participants (or guardians) included in the study. Data are reported in line with the Strengthening Reporting of Observational Studies in Epidemiology (STROBE) statement. *X. laevis* oocytes were supplied by Victor Chang Cardiac Research Institute and approved by the Garvan Institute/St. Vincents Hospital Animal Ethics Committee (Animal Research Authority 23_11).

### Statistical analysis

Continuous values were compared using the Wilcoxon rank-sum test. For very large samples (population-level analyses; comparisons across all possible missense variants), Student’s t-test was used under assumptions of normality. Categorical values were compared using Fisher’s exact test. The nominal significance threshold was set at α=0.05. Multiple testing correction was done for genotype-phenotype associations using the Benjamini-Hochberg procedure. For correlation analyses, Pearson and Spearman correlations were used to test for linear and monotonic relationships, respectively. Where appropriate, the AUCs of ROC curves across different models were compared using DeLong’s test. Model decisions were visualised using Cherkassky histograms and calibration plots.^57^ This study was carried out in the R programming language, version 4.4.2, with RStudio, version 2024.9.0.375. Packages used included tidyverse, kernlab, and ontologyX.^58^, ^59^, ^60^

### Role of funders

The funding sources had no influence on the study design, data collection, data analyses, interpretation, or writing of the report.

## Results

### Accurate and explainable prediction of GABA_A_ receptor subunit variant functional effects

We collected clinical and *in-vitro* electrophysiological data from 505 affected individuals with 272 (likely) pathogenic GABA_A_ receptor missense variants. We focused on investigating the effects of missense variants, as intronic splice-site variants, frameshift or nonsense variants are always expected to disrupt receptor function. To exclude a potential source of systematic bias, we investigated possible variant effects on splicing but found no evidence for this in our study cohort (Supplementary Methods, Table S1 and Fig. S1). Overall, 174 missense variants were unique, and the remaining cases had recurrent variants. Variants were distributed across four genes: *GABRA1* (*n* = 82), *GABRB2* (*n* = 55), *GABRB3* (*n* = 87), and *GABRG2* (*n* = 48). Functional effects were grouped as GOF (*n* = 83), LOF (*n* = 91), neutral effect (*n* = 27), and unknown (untested) effect (*n* = 71). Clinical characteristics were represented with 8185 total and 309 unique HPO terms, with a median of 14 HPO terms per individual (range 5–45; IQR 10–21 terms). Median age at seizure onset was 7 months (range 0–216; IQR 2.5–13 months). Age at seizure onset and number of HPO terms were not correlated (Spearman’s *ρ* = −0.01, *p* > 0.05), suggesting that annotation was not biased towards older individuals. There was no evidence of sex bias across our cohort (229/505 (45%) female, 227/505 (45%) male, 49/505 (10%) unknown). Median length of follow-up was 126 months (IQR 37–158 months), which we considered to be sufficient to observe all disease-relevant clinical characteristics. Given the large cohort size and comprehensive assessment for potential bias, we concluded that our cohort is likely representative of the disease spectrum.

**Fig. 1:**
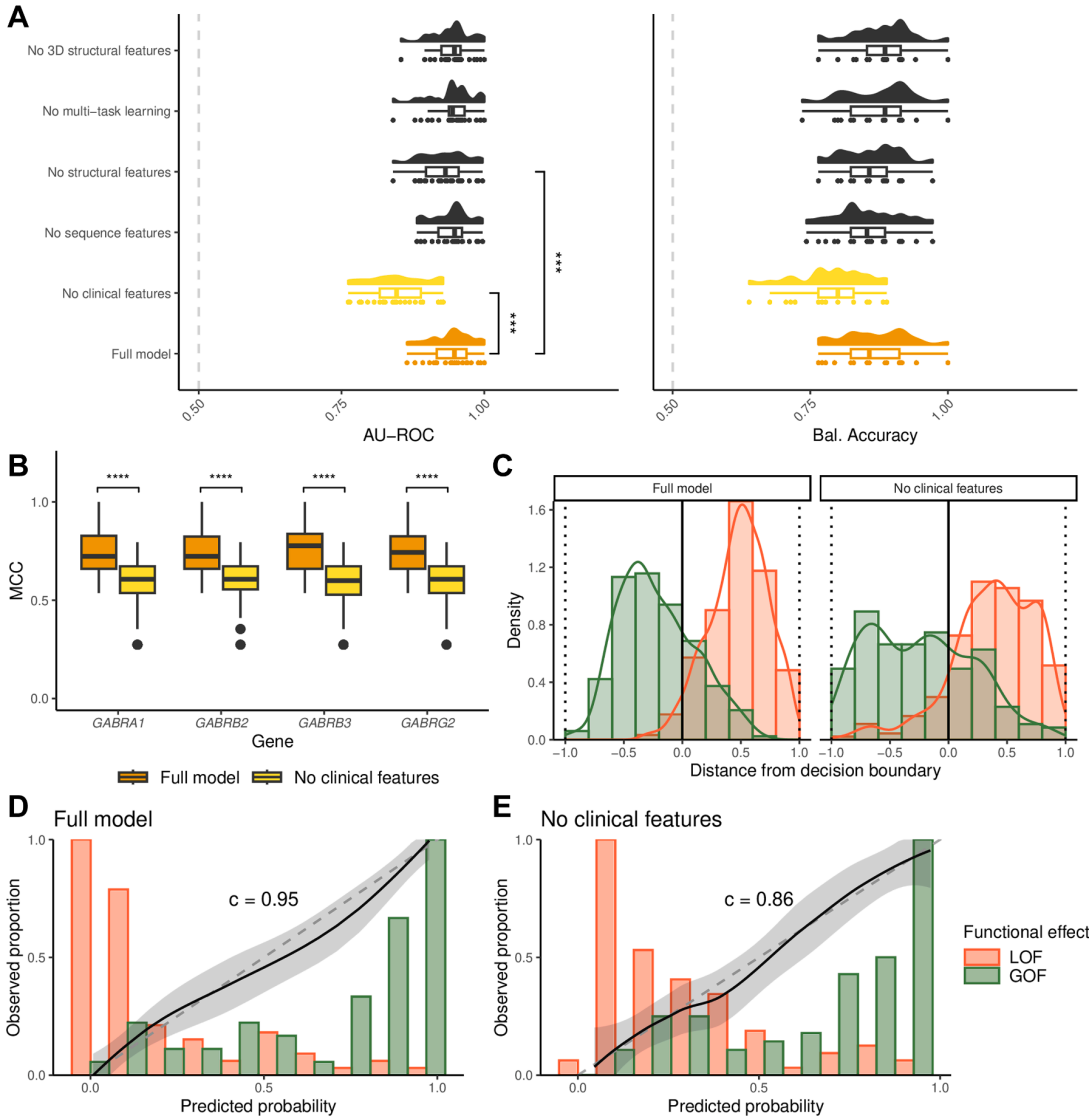
**Model performance. (A)** Model performance estimates (AU-ROC and balanced accuracy) from repeated k-fold cross-validation for the full model and after ablation, where sets of features are removed to investigate feature contribution and model stability. DeLong’s test to compare AUCs. **(B)** Model performance is stable across tasks (genes) for both the full model and the model with no clinical features. For each task, the full model offers significantly higher performance and consistently achieves a Matthews correlation coefficient (MCC) > 0.7, denoting very strong correlation to the experimentally observed effects. Two-sided Wilcoxon rank-sum test. **(C)** Cherkassky histogram of projections for the feature space used by the full model and the model with no clinical features. The bold vertical line at zero distance denotes the SVM hyperplane. **(D and E)** Calibration analysis on cross-validation folds for each model. The dashed diagonal line denotes optimal calibration. Each model was able to discriminate between LOF and GOF variants (c-statistic). Significance: ∗∗∗*p* < 0.001, ∗∗∗∗*p* < 0.0001.

The full model achieved highly accurate predictions of binary variant effect (GOF/LOF) on internal cross-validation (AU-ROC 0.943 ± 0.035). We conducted ablation studies to gain a better understanding of the model’s behaviour. While removing clinical information deteriorates performance, the overall performance was still competitive (AU-ROC 0.849 ± 0.052). The model without clinical features represents a common use case, e.g., when predicting a variant where clinical information is not yet available or when investigating synthetic variants in scanning mutagenesis. Predictive performance of the full model remained generally robust as other components were removed in turn (Fig. 1A; Table 2; Supplementary Table S4). Interestingly, a model trained exclusively on 3D structural features (pairwise residue distances) was independently predictive (AU-ROC 0.744 ± 0.065) but ablation of the same information from the full model did not deteriorate performance, suggesting that curated structural features (e.g., domains, motifs, ligand binding sites, local disorder) are sufficiently informative.

**Table 2:** Model performance estimates.

Model	Accuracy	Sensitivity	Specificity	MCC	AU-PRC	AU-ROC
Full model	0.867 ± 0.060	0.887 ± 0.082	0.846 ± 0.103	0.741 ± 0.117	0.946 ± 0.033	0.943 ± 0.035
No clinical features	0.789 ± 0.062	0.871 ± 0.090	0.699 ± 0.075	0.585 ± 0.127	0.844 ± 0.062	0.849 ± 0.052
No sequence features	0.854 ± 0.058	0.872 ± 0.088	0.833 ± 0.101	0.714 ± 0.116	0.948 ± 0.028	0.942 ± 0.032
No structural features	0.859 ± 0.053	0.881 ± 0.063	0.836 ± 0.099	0.723 ± 0.104	0.929 ± 0.039	0.929 ± 0.039
No multi-task learning	0.874 ± 0.060	0.876 ± 0.084	0.872 ± 0.085	0.753 ± 0.120	0.947 ± 0.034	0.944 ± 0.038
No 3D structural features	0.878 ± 0.055	0.894 ± 0.080	0.860 ± 0.090	0.761 ± 0.107	0.945 ± 0.031	0.942 ± 0.034

Values are shown as mean ± standard deviation. A complete list of performance estimates is provided in Supplementary Table S4. Abbreviations: MCC—Matthews Correlation Coefficient; AU-PRC—area under the precision–recall curve; AU-ROC—area under receiver operating characteristic curve.

Model performance was consistent across each of the four genes (full model: MCC 0.741 ± 0.117, model without clinical features: MCC 0.585 ± 0.127; Fig. 1B). On visual inspection of the histogram of projections (Fig. 1C) and calibration plots (Fig. 1D and E) we found that both models separated well between GOF and LOF variants (c-statistic 0.86–0.95). Lastly, we conducted a sensitivity analysis to test the hypothesis that model performance may be positively inflated by paralogous variants which strongly inform pathogenicity, phenotype and function and are independently predictive. We repeated the internal cross-validation but removed each variant from the test fold located at the same position on the channel family multiple-sequence alignment as any variant in the training fold. This did not significantly impact the performance estimates for our full model (without paralogous variants: AU-ROC 0.939 ± 0.091, two-sided Wilcoxon rank-sum test: *p* = 0.75) and the model without clinical features (without paralogous variants: AU-ROC 0.815 ± 0.054, two-sided Wilcoxon rank-sum test: *p* = 0.42). Further leave-one-gene-out and leave-one-domain-out evaluations are shown in Supplementary Fig. S2. Thus, our sensitivity analyses demonstrated that our model was not simply memorising position-, domain-, or gene-specific effects.

**Fig. 2:**
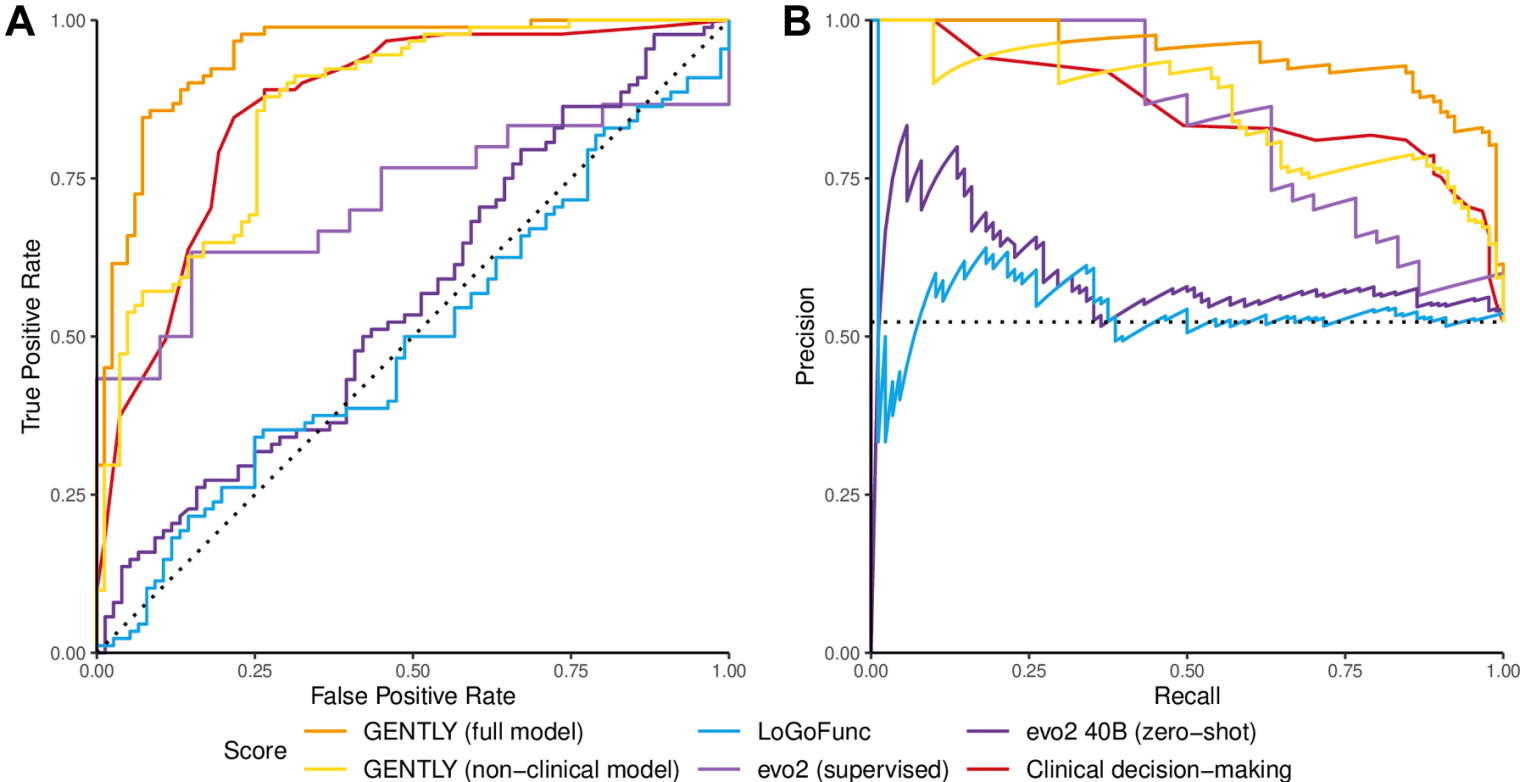
**GENTLY outperforms state-of-the-art genome-wide models and clinical decision-making. (A and B)** ROC and precision–recall curves for the full and non-clinical models compared to other functional effect prediction models (LoGoFunc, evo2) and clinical decision-making. Note that comparisons to our own method were made on out-of-fold predictions only.

Next, we compared model performance to three state-of-the-art genome-wide models that predict variant functional effect: LoGoFunc, evo2-1B, and evo2-40B. For internal validation, we compared prediction scores from *k*-fold cross-validation across the 174 unique variants in our training dataset (Fig. 2, Supplementary Table S5). For evo2, the intended use case is zero-shot variant effect prediction. To enable a fairer comparison, we also used evo2 scores to train a supervised model on the same cross-validation folds (Supplementary Methods). This approach to evo2 resulted in moderately improved performance (AU-ROC: 0.756 ± 0.191, accuracy 0.667 ± 0.170; Supplementary Fig. S3) which was still inferior to that of our model (AU-ROC 0.943 ± 0.035, accuracy 0.867 ± 0.060). As an additional baseline, we compared our model to clinical decision-making. We fit a decision tree model trained on age at onset and clinical characteristics, mirroring the approach of a clinician experienced in GABA_A_ receptor-related disorders. This resulted in a simple six-step model that was surprisingly accurate (sensitivity 0.711 ± 0.112, specificity 0.857 ± 0.084, accuracy 0.787 ± 0.052, AU-ROC 0.823 ± 0.072, AU-PRC 0.848 ± 0.066). The model found a cut-off for age at seizure onset (6 months) and the presence or absence of known genotype-phenotype correlations such as microcephaly, febrile seizures, abnormalities of movement (incl. myoclonus and hyperkinetic movement disorders), focal or generalised seizures (Supplementary Fig. S4). Both of our models still perform better, but this demonstrates that expert clinical judgement remains highly informative. The primary advantage of our full model including clinical characteristics is ease of use for clinicians who may not be experts in GABA_A_ receptor-related disorders. A complete overview of performance estimates is provided in Supplementary Table S6.

For external validation, we collected data from 197 individuals with 138 GABA_A_ receptor variants, of which 27 unique variants resulted in GOF or LOF. This small external validation set is not as statistically robust as the internal cross-validation procedure shown above but may serve as an example. Overall, 25 variants were GOF and two variants were LOF. Perhaps most interestingly, this external validation dataset included 23/27 (85%) variants in genes that were not seen by the model during training, so-called zero-shot learning (*GABRA3, GABRA5, GABRB1, GABRD*)—a highly challenging prediction problem for the model. Both of our models correctly ranked all four variants in the training distribution and still performed well even on variants in genes not seen during training (full model: AU-ROC 0.860 [95% CI: 0.692–1.000], AU-PRC 0.988 [95% CI: 0.959–1.000], balanced accuracy 0.820 [95% CI: 0.719–0.904]; model with no clinical features: AU-ROC 0.800 [95% CI: 0.615–0.931], AU-PRC 0.983 [95% CI: 0.943–1.000], balanced accuracy 0.880 [95% CI: 0.791–0.958]; confidence intervals calculated across *n* = 1000 bootstraps). Errors were primarily made on variants within two genes in the zero-shot learning group (*GABRA3, GABRD*; Supplementary Table S7). A feature-level error analysis is shown in Supplementary Fig. S5, highlighting that residues with a high accessible surface area are harder to predict. We note that 24/27 (89%) of variants in the external validation dataset were paralogous to variants in our training data. However, the sensitivity analysis we conducted above did not suggest that this would bias the performance estimate. Thus, we demonstrated proof-of-principle that information is transferable across the larger heterogeneous gene family, enabling predictions for new and emerging GABA_A_ receptor-related disorders.

Some clinical characteristics in GABA_A_ receptor-related disorders may not be seen until a certain age. For example, febrile seizures would be seen around nine months, and autism screening is usually conducted at 18–24 months of age. Therefore, not all clinical information may be available when the model is used, and clinicians may wonder how this impacts expected performance. We simulated incomplete observations by removing each HPO term in turn from variants in the training dataset and noting how the prediction confidence changed. For example, not observing microcephaly biased observations towards LOF (mean prediction −0.096) while not observing fever-related HPO terms biased observations towards GOF (mean prediction +0.052). Full results are shown in Supplementary Fig. S6. Overall, these biases were minor given the full score range (−2.32 to 1.74). Thus, our model may be used for variant interpretation in early life.

### Model predictions are biologically plausible

**Fig. 3:**
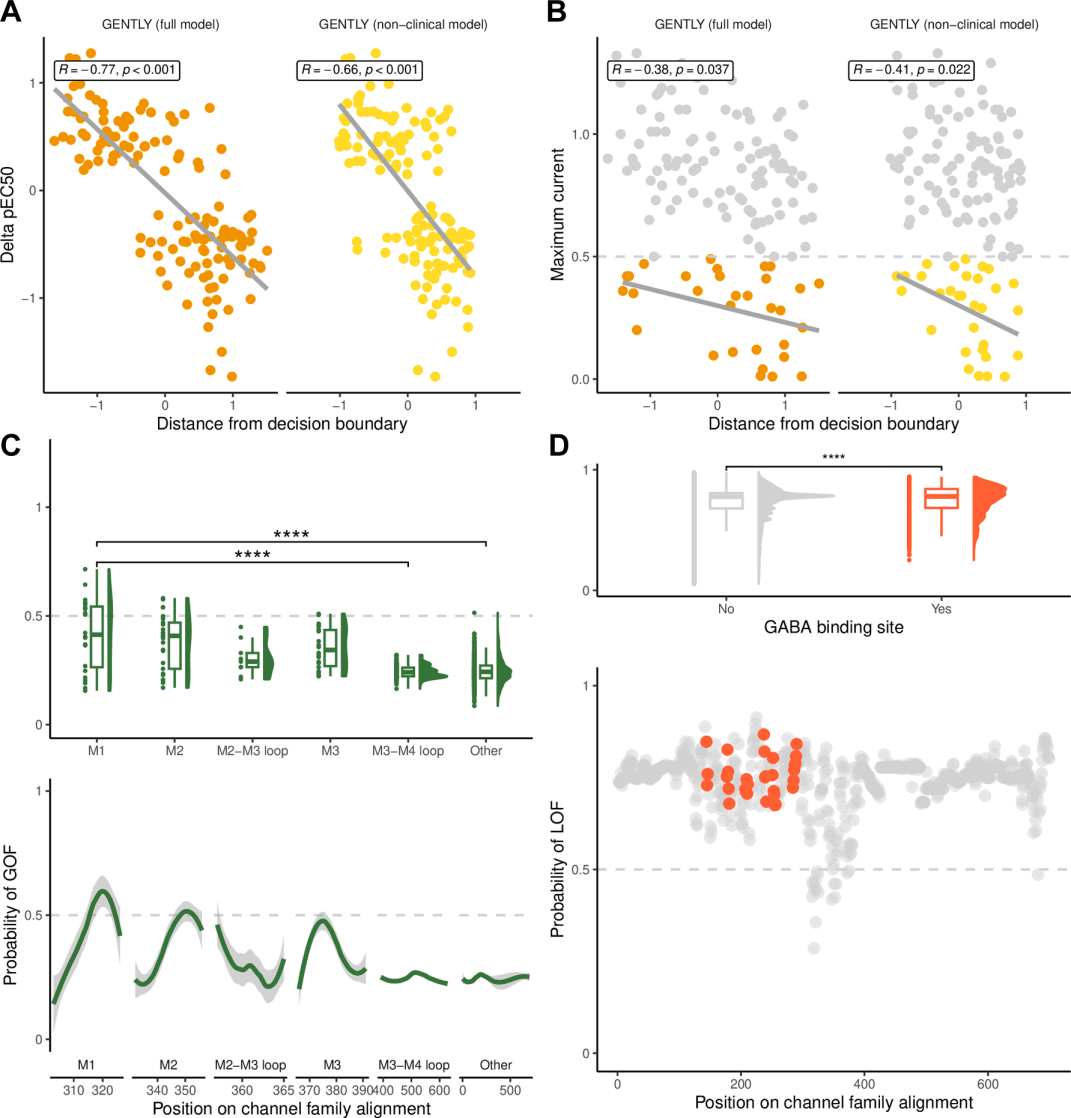
**Model predictions are biologically plausible. (A and B)** Predicted scores from the cross-validation procedure, shown as distance from the decision boundary (i.e., the raw classification used by the SVM model), correlate with continuous electrophysiological measurements (normalised sensitivity to GABA, ΔpEC50; normalised maximum current) that were not directly used during model training. Pearson correlation coefficients for both the full model and the model with no clinical features are shown separately. Normalised maximum current values above >0.5 (dashed line) are not meaningfully different from wild-type. **(C)** Model predictions correspond to expert-based hypotheses on structure-function relationships. Prediction scores for all possible GABA_A_ missense variants, averaged for each residue position, were compared across functionally relevant domains. Two-sided t-test. **(D)** Variants at GABA binding sites are significantly more likely to be predicted as LOF by the model. Two-sided t-test. Significance: ∗∗∗∗*p* < 0.0001.

For further validation, we compared our model predictions to continuous experimental labels: the change in GABA sensitivity compared to wild-type receptors (ΔpEC50) and the normalised maximum current. Information from these measurements was used to assign the label (GOF or LOF) during experimental characterisation (Methods). However, we hypothesised that prediction confidence would further correlate with the severity of change in sensitivity to GABA or maximum current beyond simple cut-offs. This would also allow for semi-quantitative assessment of variant functional effect, beyond the binary classification provided by previous models. Indeed, we found that scores correlated strongly with sensitivity to GABA (Fig. 3A; Pearson’s correlation coefficient *r* = 0.66–0.77; *p* < 0.001) and moderately with maximum current (Fig. 3B; *r* = 0.38–0.41; *p* < 0.05) in the test dataset from cross-validation. Thus, variants that are more confidently predicted as GOF or LOF cause more severe electrophysiological changes.

Next, we investigated whether our model conforms to expected structure-function relationships. This would offer additional evidence that our model has learnt biologically plausible information. For this, expert biophysicists (PKA, NLA) prespecified two hypotheses based on literature. The first hypothesis was that variants at GABA binding sites are more likely to be LOF. Second, variants within the transmembrane segments M1 and M2 are more likely to be GOF than those in extracellular coupling loops, ∼25% of which would be expected to be GOF. For this analysis, we calculated predictions for all possible missense variants in GABA_A_ receptor subunits averaged for each position on the channel family alignment, to allow for comprehensive assessment beyond the positions covered by the training data. Variants within the training data and all paralogous variants were removed for this analysis. We found that variants at GABA binding sites were more likely to have a higher LOF score in our model (two-tailed t-test; mean 0.740 vs. 0.753, *t*(10715): −16.20, *p* < 0.001; Fig. 3C), and accordingly were enriched for variants predicted as LOF (Fisher’s exact test; OR 1.50, 95% CI: 1.36–1.66, *p* < 0.001). Similarly, variants within M1 and M2 were enriched for variants predicted as GOF compared to coupling loops (Fisher’s exact test; OR 6.73, 95% CI: 6.09–7.45, *p* < 0.001; Fig. 3D). Thus, our model successfully learnt expert-level structure–function knowledge.

### Model predictions in cases and controls

**Fig. 4:**
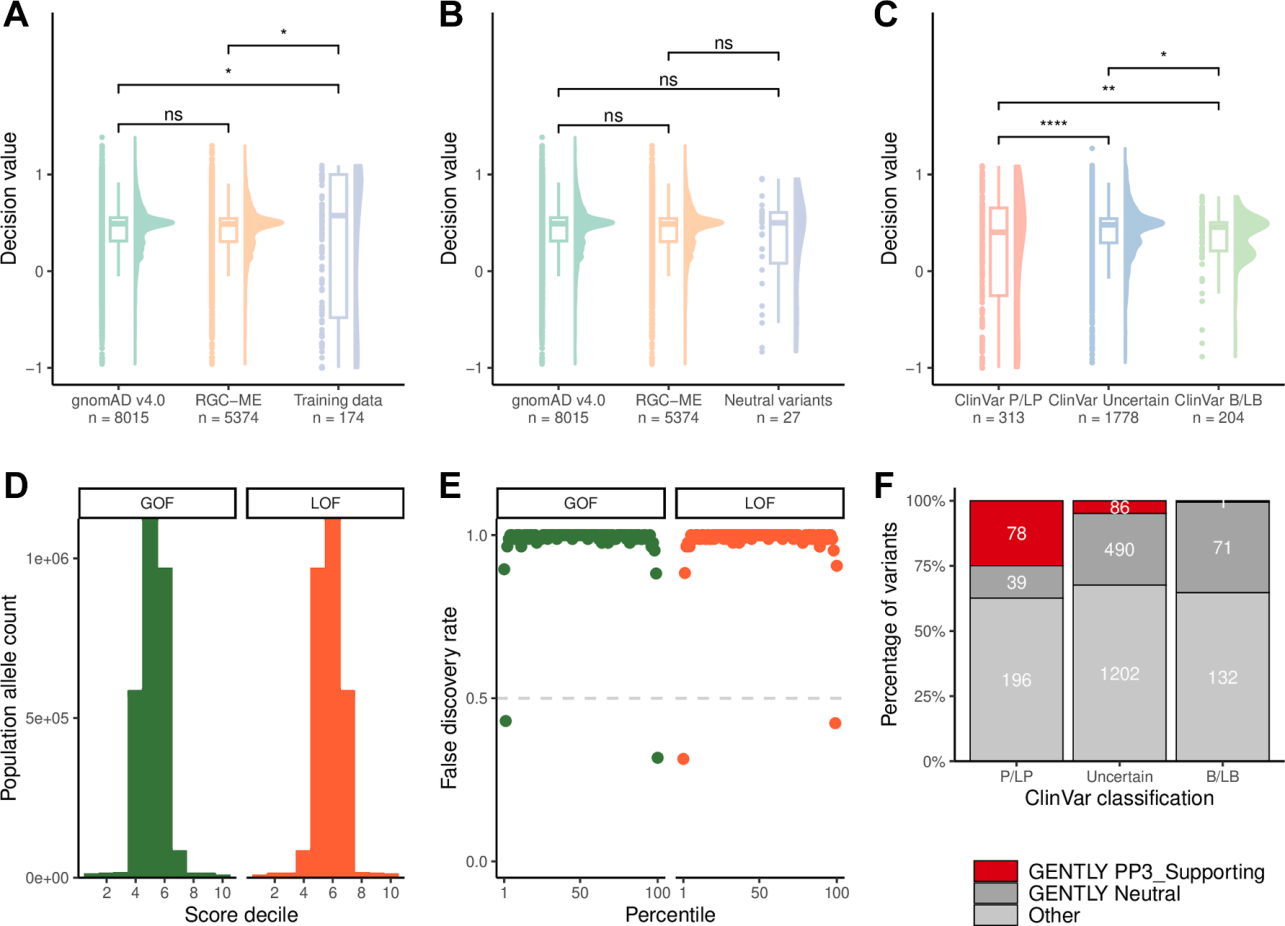
**Model predictions in cases and controls. (A)** Model predictions, shown as distance from the decision boundary, are similar across two sets of population variants (gnomAD V4.1.0, RGC Million Exome Variant Browser) but distinct from training variants with functional effects (GOF/LOF). Two-sided t-test. **(B)** Model predictions are not significantly different for population variants and variants with no functional effect (neutral). Two-sided t-test. **(C)** Pathogenic or likely pathogenic (P/LP) variants from ClinVar have scores similar to functional variants, while benign or likely benign (B/LB) variants fall within the score interval of population variants. Two-sided t-test. **(D)** The majority of population variants fall within clearly defined score deciles. **(E)** Variants within the top score percentile can be separated from population variants. **(F)** Variant annotations with score ranges may provide additional evidence for ClinVar variants. Variants observed in the training data were removed for this analysis. Significance: ns—not significant, ∗*p* < 0.05, ∗∗*p* < 0.01.

Future clinical implementation of functional prediction algorithms requires a better understanding of how these models perform on variants outside of the training distribution. Previous models work under the assumption that the variant of interest has already been classified as (likely) pathogenic according to ACMG criteria, but variant interpretation in rare genetic epilepsies is challenging and prone to misclassification errors. Thus, users may try to predict functional effects of variants that are not disease-related in the individual. Therefore, we investigated how our model behaves when provided with presumably neutral variants from large population databases (gnomAD v4.0, RGC Million Exome Variant Browser). We found that mean scores were different for population variants compared to (likely) pathogenic variants from our training dataset (Fig. 4A) but were similar when compared to 27 variants with neutral functional effect (Fig. 4B). This finding was independently validated using ClinVar, where we found that the score distribution of (likely) pathogenic variant was similar to that of variants from our training data, while the score distribution for variants of uncertain significance and (likely) benign variants was more similar to that of population variants (Fig. 4C). Most population variants fall within the 4th to 7th scores deciles (probability of GOF: 0.208 < x < 0.283; probability of LOF: 0.717 < x < 0.792) which provides narrow bounds within which predicted variants should be flagged as potential population or neutral variants, prompting variant reassessment (Fig. 4D).

This naturally raises the question whether predictions from our model can be used to distinguish population from pathogenic variants, a task broadly similar to that of pathogenicity prediction algorithms. Variants within the top score percentile (probability of LOF: >0.928; probability of GOF >0.850) were much more likely to be pathogenic variants (Fisher’s exact test; OR 153, 95% CI: 92–260, *p* < 0.001) while variants within the top two score percentiles (probability of LOF: >0.920; probability of GOF >0.729) were still strongly enriched for pathogenic variants (Fisher’s exact test; OR 6.74, 95% CI: 3.05–13.41, *p* < 0.001). Due to the high number of population variants (*n* = 13,389), the false-discovery rate (FDR) for the top score percentile was still 31.4% (Fig. 4E). However, these calculations were done under strict assumptions where any variant irrespective of population allele count (AC) was counted. When this assumption was relaxed (e.g., AC > 5), given the role of GABA_A_ receptor variants in common epilepsies and mild phenotypes, the expected performance improved (FDR 4.8%; Supplementary Table S8). Thus, our model can provide complementary evidence when assessing variant pathogenicity but should not be the only source of evidence.

Given the high accuracy of our functional predictions and separability from population variants, our model may be able to provide additional information for variant assessment. To facilitate use in weighted evidence frameworks, we calculated score thresholds calibrated to ACMG evidence strengths using a likelihood ratio-based approach (Supplementary Methods, Fig. S7, and Table S9). We assessed the applicability of these calibrated thresholds by obtaining predictions for 2380 variants from ClinVar, of which 2295 variants were not observed in our training data. Under conservative estimates, our model can provide additional *in silico* evidence (ACMG PP3) for 87/1778 (5%) to 78/313 (25%) of variants (Fig. 4). This additional evidence may prompt early variant reassessment and reclassification, potentially resulting in more timely and accurate genetic diagnoses. However, this line of evidence does not replace or supersede other methods for pathogenicity prediction, which we therefore benchmarked across our cohort.

We have previously raised the issue that variants in rare genetic epilepsies are often misclassified and highlighted how our model output can be interpreted using careful thresholds to reduce the risk of misclassification and thus the consequences of model misuse. This naturally raises the question of why we chose to directly design a functional prediction model, without gene-specific pathogenicity prediction. Pathogenicity classification goes far beyond the direct use of *in silico* scores, which play only a limited role in the ACMG classification framework. Further, there already are highly accurate proteome-wide pathogenicity predictors which we have recently benchmarked in ion channel and transporter disorders. We investigated their accuracy in our dataset, comparing 174 (likely) pathogenic variants from our training data to 2591 variants from population databases (gnomAD v.4.10, RGC-ME; AC>3). We found that AlphaMissense (AU-ROC 0.905) and REVEL (AU-ROC 0.864) performed well, and REVEL correctly classified variants in our training data according to calibrated ACMG evidence strengths. Variants from ClinVar were not used to avoid circular reasoning from training data of each pathogenicity prediction algorithm. Thus, currently available pathogenicity prediction models are sufficiently useful for variants in genes encoding GABA_A_ receptor subunits (Supplementary Fig. S8), while our model further allows for LOF/GOF prediction.

So far, we have investigated the score distribution and potential utility across our own cohort, an external validation dataset, and large population databases. It remains unclear whether the same findings hold across variants with smaller effect sizes from large epilepsy sequencing studies, which would broaden the applicability of our model predictions. For this, we calculated model predictions for 1483 GABA_A_ receptor subunit missense variants from years 1–5 of the Epi25 Collaborative, representing 20,979 individuals with epilepsy (Supplementary Methods). We observed variants within the top score percentile only for the subgroup with DEE. Functional effect scores correlated with pathogenicity prediction scores (AlphaMissense *p* = 0.032, EVE *p* < 0.001, REVEL *p* = 0.004; Pearson’s correlation coefficient) and variant enrichment in cases vs. controls for the DEE and epilepsy subgroups (*p* = 0.015 and *p* = 0.017, respectively). Further, prediction scores could separate variants across phenotypic subgroups (Supplementary Fig. S9). Thus, we found that our score has broad utility in large cohort studies. Future studies may investigate whether these annotations can improve risk estimates from polygenic scores.

### Predicting clinical characteristics

**Fig. 5:**
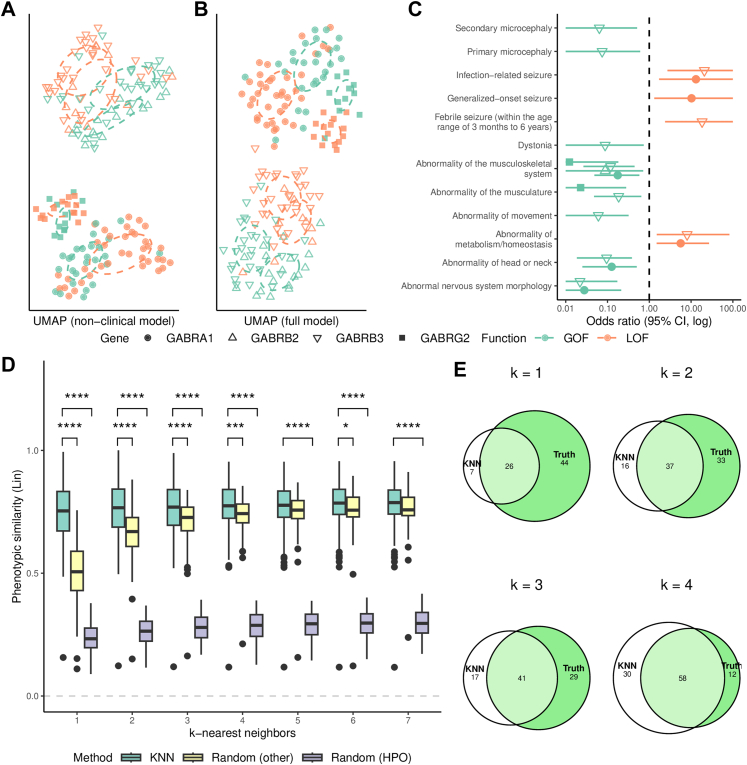
**GENTLY may provide further clinical insight into GABA_A_ receptor-related disorders. (A)** UMAP (Uniform Manifold Approximation and Projection) plot of the feature space of the model with no clinical features. Note that genes and functional effects are visually separable. Normal distributions are shown as dashed circles. **(B)** UMAP plot of the full model, where the inclusion of clinical features improves separability and thus prediction of functional effects. **(C)** Clinical spectrum of GABA_A_ variants by functional effect. Forest plot of relative enrichment (Fisher’s exact test) of HPO terms across functional subgroups and genes. **(D)** The feature space of the model with no clinical features (A) can be used to estimate the likely phenotype with a k-nearest neighbour approach. Two-sided t-test. Random (other) corresponds to phenotypes based on any non-neighbour variants. Random (HPO) corresponds to phenotypes based on random human phenotype ontology (HPO) terms sampled from the ontology. Baseline estimates were repeated 10-fold. Reconstructed phenotypes are compared to the clinically observed phenotype using the Lin similarity measure (range 0–1, with higher values denoting higher phenotypic similarity). **(E)** Venn diagram demonstrating the overlap of reconstructed vs. observed HPO terms for different values of k in k-nearest neighbours. The variant *GABRA1* p.V270I is shown as an example. Significance: ∗*p* < 0.05, ∗∗*p* < 0.01, ∗∗∗*p* < 0.001, ∗∗∗∗*p* < 0.0001.

It would be clinically useful to directly predict clinical characteristics from variant information, similar to the *SCN1A*-Epilepsy Prediction Model which accurately distinguishes between mild and severe *SCN1A*-associated phenotypes and thus informs prognostic counselling and early-life decisions.^61^ We noted that our model representation already included the same information used for this clinical prediction approach: Clinical characteristics were weighted by age at seizure onset and integrated with variant-level structural information to learn a shared representation which separates between functional subgroups (Fig. 5A and B). These functional subgroups were enriched for distinct clinical characteristics including febrile seizures (*GABRB3* LOF; Fisher’s exact test; OR 18.37, 95% CI: 2.39–838, *p* < 0.001), microcephaly (*GABRB3* GOF; Fisher’s exact test; OR 0.06, 95% CI: 0.01–0.51, *p* < 0.001), and dystonia (*GABRB3* GOF; Fisher’s exact test; OR 0.09, 95% CI: 0.01–0.73, *p* < 0.05), as well as other higher-order HPO terms (Fig. 5C). These findings both recapitulate and extend the phenotypic spectrum of associated neurodevelopmental disorders. An overview across 1072 HPO terms, of which 108 terms had nominally significant gene-function-phenotype associations, is provided in Supplementary Table S10.

To explore the feasibility of phenotype prediction from variant-level structural information, we employed a *k*-nearest neighbour approach: For each variant, we found the *k* nearest variants in the feature space of the model with no clinical features (Fig. 5A) and compared the set of their HPO terms to the variant of interest. Using this approach, meaningful variant phenotypes could be predicted (median Lin similarity 0.754 IQR 0.161 vs. random baseline median 0.506 IQR 0.160; two-sided t-test: *p* < 0.0001; Lin similarity range 0–1, with higher values denoting higher phenotypic similarity; Fig. 5D). For example, 26/70 (37.1%) to 58/70 (82.9%) of clinical characteristics of the variant *GABRA1* p.V270I could be reconstructed using *k* = 1 to 4 nearest variants in feature space (Fig. 5E). Thus, the variant representation of our model may be used to infer likely clinical characteristics, pending further validation.

## Discussion

In this study, we used comprehensive clinical and electrophysiological data from 505 affected individuals with 272 GABA_A_ receptor variants to train a machine learning model to predict variant functional effects (GOF or LOF). This model was then externally validated on a further 197 affected individuals with 138 GABA_A_ receptor variants. We found that our model outperforms current state-of-the-art genome-wide functional effect prediction algorithms by a wide margin. This was expected: First, previous studies have demonstrated that gene-specific model design can make efficient use of expert knowledge by explicit representation of underlying mechanisms (e.g., gene family taxonomy, specific binding sites and motifs, pore and membrane geometry) which the genome-wide deep learning models may not represent well.^29^, ^30^, ^31^, ^32^, ^33^ Further, this biologically grounded design makes our model more plausible and robust as demonstrated by conformation to expert-based structure–function hypotheses. Second, genome-wide predictors work on more general ‘genomic’ definitions of GOF (e.g., hypermorphs, where protein activity is increased, and neomorphs, where novel functions are acquired) and LOF (e.g., destabilisation of protein folding).^62^^,^^63^ These assumptions do not seem to reflect the very specific GOF/LOF mechanisms observed in the electrophysiological characterisation of ion channels and transporters. Thus, specialised prediction models are needed.

Previous models were limited by data availability as electrophysiological data is time-consuming and expensive to acquire, which has resulted in the use of data from non-human channels or inference of labels from observed phenotypes.^29^^,^^32^ Accordingly, validation datasets were small and actual model performance was largely uncertain. Here, we utilised a large family-specific clinical and electrophysiological dataset. We aimed to minimise noise or uncertainty in our dataset, improving predictive power and reliability: First, clinical data were harmonised by independent assessment from experienced clinicians and further standardised using ontological reasoning with HPO terms.^40^ Second, all electrophysiological data was recorded at the same laboratory using receptor assemblies that directly reflect heteropentamer composition in heterozygous carriers.^64^^,^^65^ Together, these steps ensured data quality and resulted in a highly calibrated model where prediction confidence directly correlated with quantitative measurements for the severity of the electrophysiological changes.

Functional prediction algorithms are designed to address a key translational gap in neurodevelopmental disorders: While the increasing utilisation of next-generation sequencing has outpaced the availability of direct experimental evidence, ongoing natural history and precision medicine trials (including both specific agonists/antagonists, and antisense oligonucleotide or gene therapies) in rare disease require functional evidence for patient identification and stratification (e.g., ClinicalTrials.gov IDs NCT06872125, NCT05419492). Currently, *in-vitro* electrophysiology remains the gold standard and no functional prediction algorithms have been approved for clinical use. However, if we want to pave the way for their implementation, we must address two questions: First, how does the model respond when provided with a (likely) benign variant from the general population? This represents a common use case where the variant of interest has initially been misclassified. Here, we provide a score range common for population variants that should prompt variant reassessment (probability of GOF: 0.208 < x < 0.283; probability of LOF: 0.717 < x < 0.792), thus minimising risk. Further, we provide top score percentiles for high-confidence predictions (probability of LOF: >0.928; probability of GOF >0.850), and ACMG-calibrated evidence thresholds. Second, does our model offer additional insight into case variants which may prompt reclassification? We note that the ACMG criterion PP3 (computational and predictive data) currently only applies to a set of well-calibrated predictors (e.g., REVEL).^66^^,^^67^ Similarly, ACMG criterion PS3 (functional data) is reserved for well-established functional assays.^68^ Yet, our model may provide complementary evidence. Future use will require additional prospective validation and integration in multimodal evidence frameworks.

Regarding limitations, we note that the performance of our full model deteriorated on external validation in two specific genes not observed during training (e.g., *GABRA3, GABRD*). This was not unexpected and rather reflects that the model learnt valid information from the gene family: First, LOF in either gene has not yet been clearly linked to epilepsy and thus does not align with the established genotype-phenotype correlations.^18^^,^^69^ Second, the genotype of *GABRA3* is an outlier as it is the only disease-associated X-linked gene in the family.^12^ Third, *GABRD* has rather different criteria for functional changes.^18^ Our recommendation would be to first establish gene-disease validity (i.e., not assuming causality for genes not yet linked to human disease), then assessing variant pathogenicity using ACMG criteria, before using the model. We would suggest using the structural model with the score thresholds provided above, complemented by expert assessment based on known genotype-phenotype correlations, optionally including the clinical decision tree we have provided (Supplementary Fig. S4).^14^^,^^15^ A combined workflow and proposed evidence framework for variant assessment using the provided annotation files and online resources is shown in Supplementary Fig. S10.

Some potential bias applies: *(i)* clinical assessment of the individuals in our study may have been biased by previously observed genotype-phenotype correlations; *(ii)* continuous labels (e.g., ΔpEC50) were partially used to assign categorical labels (GOF/LOF). However, these were only part of the full electrophysiological characterisation and were done independently while the added value lies in the correlation with prediction confidence beyond a simple cut-off value; *(iii)* previous experimental data used during training indirectly informed knowledge on structure-function relationships. We consider it to still be useful that the model conforms to these observations, making expert biophysicist knowledge available to clinical users. In summary, we consider these possible biases to be acceptable as they represent current expert knowledge on GABA_A_ receptor subunits. Importantly, the model still performed well on external validation on a set of variants that were recorded after the training data. Lastly, we consider our approach to phenotypic learning and prediction to be exploratory pending larger cohorts and methodological refinement similar to that of the *SCN1A*-Dravet model.^61^

Functional prediction of GABA_A_ receptor subunit variants is feasible, highly accurate, biologically plausible, and useful across large clinical cohorts. Our approach brings together methodological advances in machine learning with recent studies on the expanding spectrum of GABA_A_ receptor-related disorders. The model is fully integrated within the Gene Portals framework, details of which are available in a recent preprint, allowing for retraining as new data become available.^70^ Pending prospective clinical validation, variant functional effect prediction algorithms represent a hypothesis-generating tool that may inform timely diagnosis and genetic counselling. Their potential role in patient identification and stratification for precision therapy trials remains observational and requires validation in interventional settings.

## Contributors

CMB and RSM conceptualised the study. CMB carried out formal analysis, visualisation, and wrote the manuscript. VWYL, SE, SXNL, ASHK, NLA, and PKA designed, collected, analysed and interpreted data from electrophysiological experiments. SO and RSM recruited and phenotyped participants. RSD, TB and DL carried out model calibration and provided the web portal. JK and NP calculated scores for model benchmarking. PKA, MC, HL and RSM supervised the project. PKA and RSM accessed and verified data. All authors contributed to drafting and reviewing the manuscript. All authors read and approved the final version of the manuscript for submission.

## Data sharing statement

Model predictions for all possible missense variants are openly available on Zenodo at 10.5281/zenodo.17800749, including annotations of the score ranges and evidence calibration thresholds shown below. An interactive website including our model and cohort summary statistics is openly available (https://gaba-portal.lalresearchgroup.org/). Raw clinical and experimental data that support the findings of this study are available from the corresponding author upon reasonable request.

## Declaration of interests

None of the authors have interests to declare.
