# Supplementary material for: Accurate prediction of gain- and loss-of-function missense variants in GABAA receptors
Source: eBioMedicine. 2026 Aug 3;131:106417. doi: 10.1016/j.ebiom.2026.106417 (PMC13470315; doi:10.1016/j.ebiom.2026.106417)
Supplement: Supplementary file 1 [file mmc1.pdf]

Supplementary material for "Accurate prediction of gain- and loss-of-function missense variants in GABAA receptors"

Christian M. Boßelmann[1*], Sebastian Ortiz[2,3], Rebekka Dahl[2-4], Vivian W. Y. Liao[5], Serene El-Kamand[5], Susan X. N. Lin[5,6], Anthony Sze Hon Kan[5], Tobias Brünger[4], Dennis Lal[4,7-9], Holger Lerche[1], Jules Kreuer[10], Nico Pfeifer[10], Mary Chebib[5], Nathan L. Absalom[5,11], Philip K. Ahring[5], Rikke S. Møller[2,3*]

Table of Contents

Supplementary Methods	2
Supplementary Figure 1	5
Supplementary Figure 2	6
Supplementary Figure 3	7
Supplementary Figure 4	8
Supplementary Figure 5	9
Supplementary Figure 6	10
Supplementary Figure 7	11
Supplementary Figure 8	12
Supplementary Figure 9	13
Supplementary Figure 10	14
Supplementary Table 1	16
Supplementary Table 2	31
Supplementary Table 3	32
Supplementary Table 4	33
Supplementary Table 5	40
Supplementary Table 6	41
Supplementary Table 7	43
Supplementary Table 8	51
Supplementary Table 9	69
Supplementary Table 10	76
Supplementary References	77

Supplementary Methods

Automated ACMG classification

Variants were initially classified by the submitter. We repeated automated ACMG classification in line with the Gene Portals framework.[1] The following criteria were assigned: *(i)* BA1 (very strong) if max gnomAD AF > 1e-04; *(ii)* BS1 (strong) if max gnomAD AF > 2e-06; *(iii)* PM2 (supporting) if max gnomAD AF equal to or less than 0; *(iv)* PS1 (strong) if another (likely) pathogenic case with the same amino acid exchange has been reported on ClinVar (not counting cases from our own database); *(v)* PM5 if another (likely) pathogenic case with a different exchange at the same position has been reported; *(vi)* PP3/BP4 based on ACMG-calibrated REVEL score thresholds[2]; *(v)* PM1 (moderate) if the variant is located in a PER[3]; *(vi)* PS3 strong if variant has been functionally tested to be LOF/GOF.

We did not include information from paralogs, since there are no established guidelines for GABAA receptor-related disorders yet.[4] Further, automated variant-level assessment did not include any criteria related to inheritance, segregation analysis, or phenotype specificity. Therefore, actual classifications are likely to be higher. Additionally, only variants with a known functional effect (LOF/GOF; criterion PS3) were included in the final model. Overall, automated classification was feasible for 98% of variants (323/329). A single variant was classified as (likely) benign: *GABRB3* Glu357Lys, reported in an individual who had onset of bilateral tonic-clonic seizures at age 14 years with no other phenotypic abnormalities. The variant was found to have a neutral functional effect and was not used during analysis.

Supervised learning on evo2 embeddings

For all GABA receptor SNVs, we extracted embeddings for the reference sequence and the SNV using block 20 of the Evo 2 7B model. The mean value of these embeddings was then calculated to yield vectors of length 4,096 for both the reference and the variant sequences. The concatenation of these embeddings was then used as inputs to train a classification model which is composed of a feed-forward neural network comprising two hidden layers. The neural network takes an input of dimension D = 8,192 and processes it through fully connected layers of sizes 128 and 64 neurons, respectively, before outputting a probability that the given SNV is Loss-of-Function. Each hidden layer is followed by ReLU activation, batch normalisation,

and dropout (p = 0.2). The final layer uses sigmoid activation to produce binary classification probabilities. The complete architecture is as follows:

Input(8,192) → Linear(128) → ReLU → BatchNorm → Dropout(0.2) → Linear(64) → ReLU → BatchNorm → Dropout(0.2) → Linear(1) → Sigmoid

The dataset was evaluated using a 25-fold stratified cross-validation procedure. For each fold, the model was trained using Adam optimization (learning rate: 3×10^{-4}, batch size: 32). The training employed early stopping (patience: 50), learning rate reduction on plateau (factor: 0.5, patience: 15, min_lr: 1×10^{-6}), and gradient clipping (max norm: 1.0). The model was trained for up to 300 epochs using binary cross-entropy loss, with the model that demonstrated the strongest performance on the validation fold selected for final evaluation. The model that was trained with the embeddings from block 20 achieved a mean AUROC of 0.755 ± 0.19 across the 25 folds.

Computational evidence calibration

We calibrated the functional scores for American College of Medical Genetics and Genomics (ACMG) computational evidence using the likelihood ratio-based framework adapted from Pejaver et al. 2022. ClinVar variants classified as benign or likely benign were grouped as benign, and pathogenic or likely pathogenic variants were grouped as pathogenic. All variants used for model training were excluded from calibration. For each score value, an adaptive local neighborhood was defined until the region contained a minimum of three benign and three pathogenic variants and at least ten variants in total. Local likelihood ratios were calculated as the probability of observing a variant with a score in that region among pathogenic variants divided by the corresponding probability among benign variants. Uncertainty was assessed by 10,000 bootstrap resamplings, and confidence bounds were taken from the fifth and 95th percentiles of the bootstrap distribution. Evidence levels were assigned when the lower confidence bound of the likelihood ratio exceeded the Pejaver et al. 2022 thresholds for computational PP3 evidence. Supporting, moderate and strong pathogenicity correspond to likelihood ratios of 2.406, 5.790 and 33.53. Benign evidence (BP4) was assessed by the upper confidence bound of the likelihood ratio meeting the inverse thresholds. Score cutoffs were defined as the lowest score that consistently met the required evidence strength.

Annotation of splice-altering variants

In principle, missense variants that affect splicing behavior (e.g., creating a cryptic splice site) may lead to variant effects that are not reflected by the experimental assay used in this study, as the cDNA construct will not undergo the same splicing change. To investigate this, we annotated all variants with SpliceAI, a state-of-the-art 32-layer deep neural network that predicts splicing events.[5] Next, we filtered variants using the recommended high-recall threshold of $P > 0.2$ for any splicing event (Acceptor Loss, AL; Donor Loss, DL; Acceptor Gain, AG; Donor Gain, DG). Variants above this threshold were further investigated with Pangolin[6] and SpliceAI-visual via MobiDetails.[7] Variants were classified as potentially splice-altering if they exceeded the standard thresholds of SpliceAI and Pangolin.

Common and rare variants from case-control studies

To investigate the utility of our score across large cohorts from epilepsy sequencing studies, we obtained data from the Epi25 Collaborative. For this, we downloaded variant-level results across all genes encoding GABAA receptor subunits from the Epi25 WES Browser (https://epi25.broadinstitute.org; data release January 23, 2023). Technical details on sequencing, variant calling and quality control can be found in the source publication.[8] Variants that were present in our training dataset were removed to avoid circularity. We used Fisher's exact tests on case versus control allele counts to calculate relative enrichment.

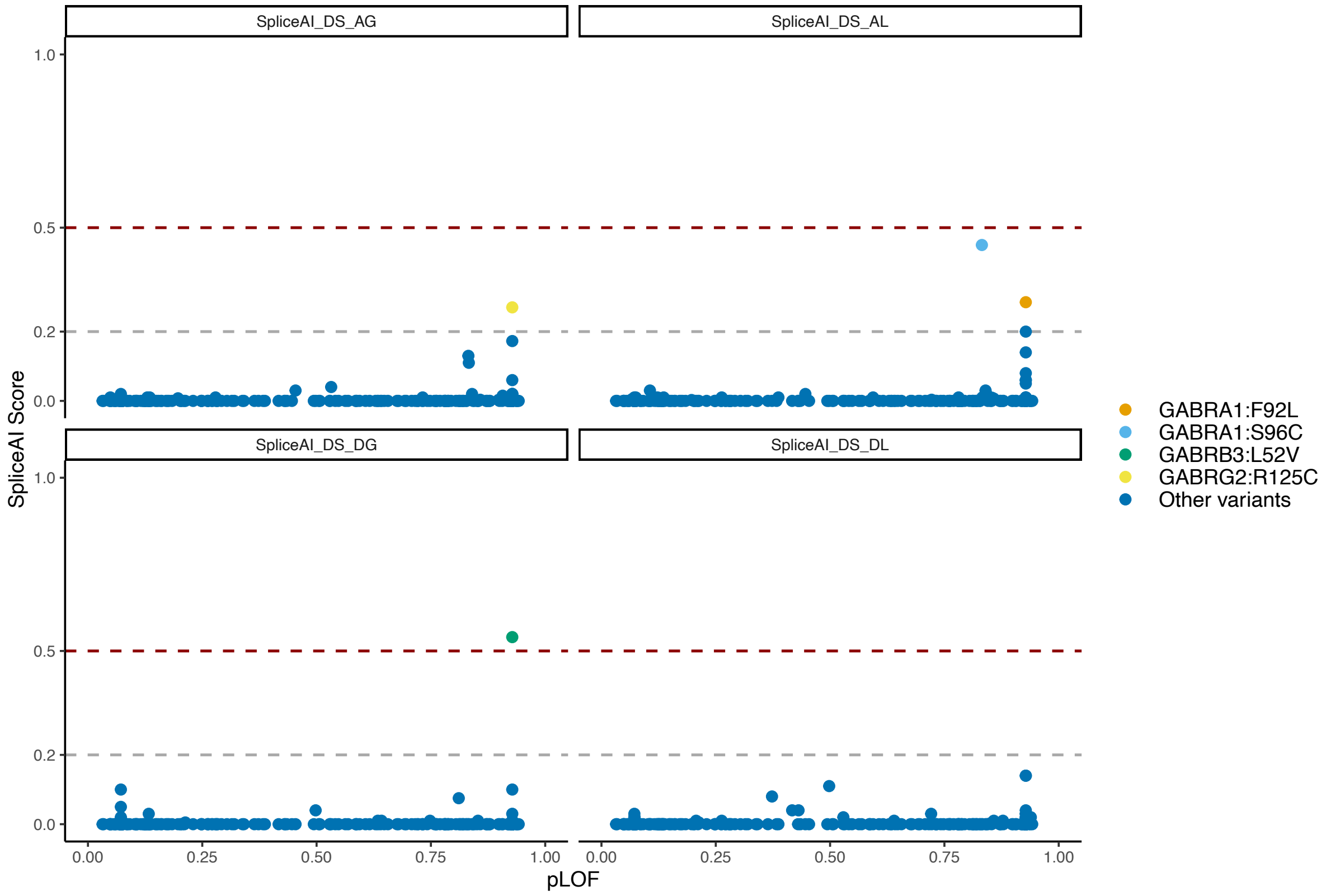

Supplementary Figure 1. Predicted splicing effects of missense variants in the study cohort. The predicted impact on splicing is shown for each splicing impact separately (Acceptor Loss, AL; Donor Loss, DL; Acceptor Gain, AG; Donor Gain, DG) and across the predicted functional effect score from our model (pLOF). SpliceAI thresholds are shown in grey (high-recall threshold, $P > 0.2$) and red (standard threshold, $P > 0.5$). The high-impact threshold, $P > 0.8$, is not shown. Variants above the high-recall threshold are shown in colour.

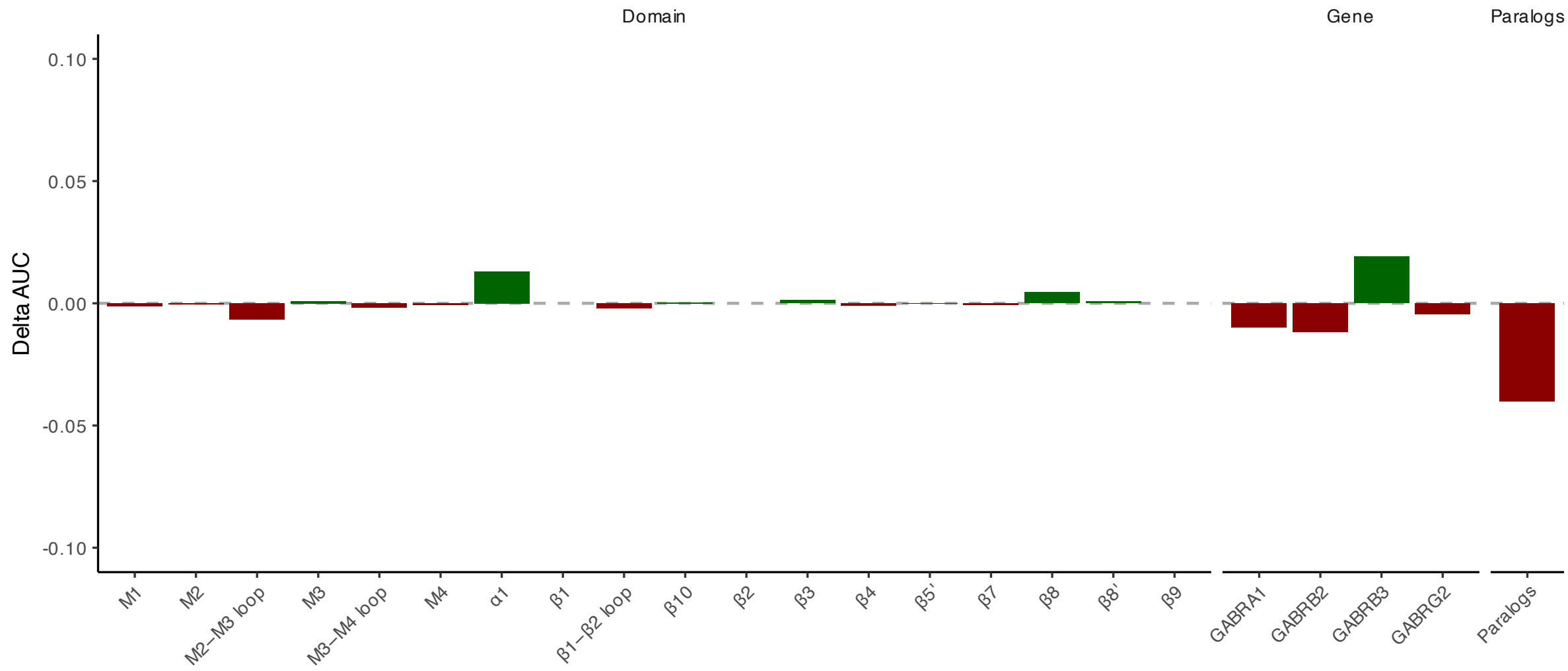

Supplementary Figure 2. Leave-one-domain-out and leave-one-gene-out evaluations. The results of the paralog sensitivity analysis are shown for comparison. For each evaluation, the difference to the baseline AUC is shown. There were no significant differences between AUCs (DeLong's test).

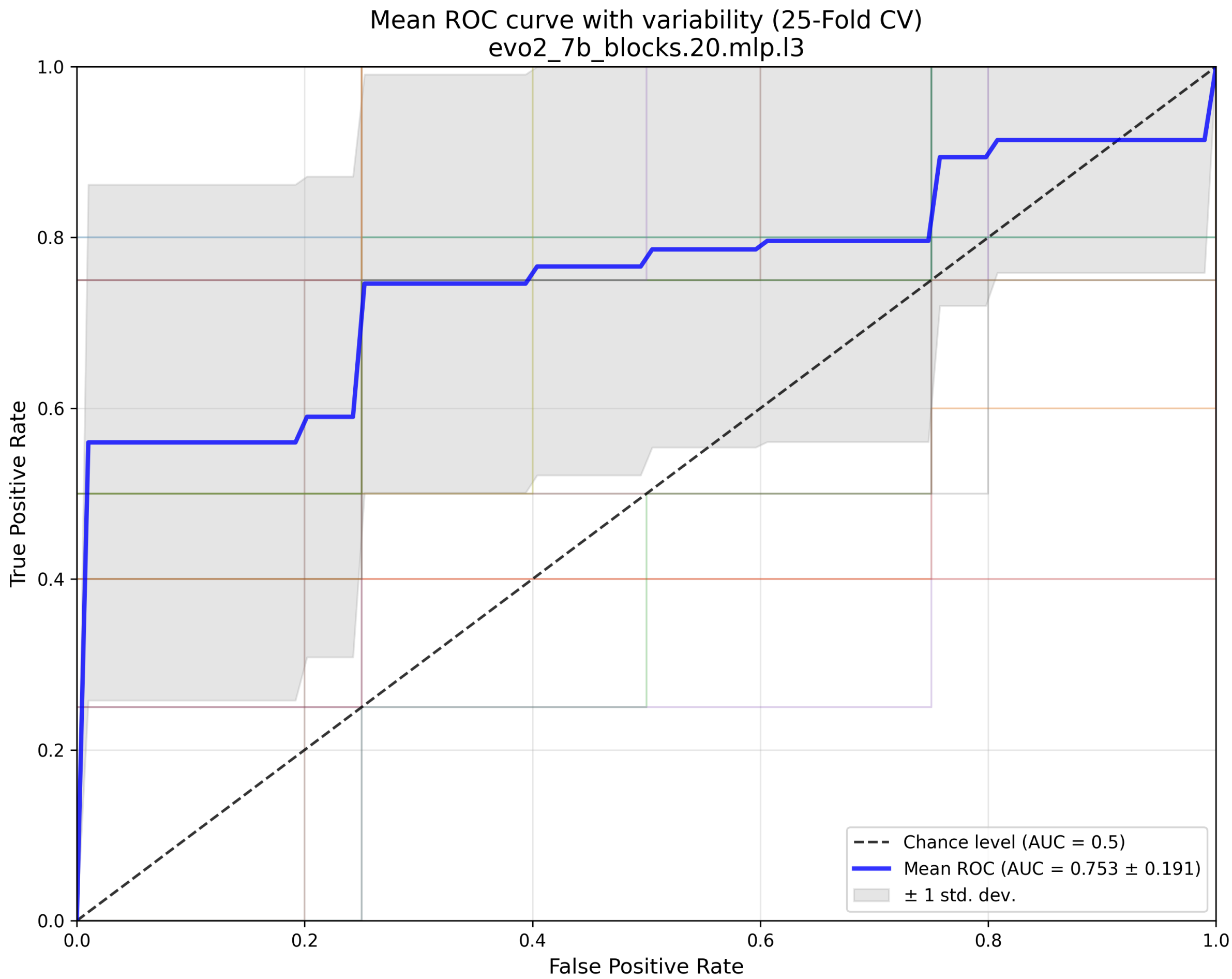

Supplementary Figure 3. Performance of a supervised evo2 classifier for functional effect prediction (GOF/LOF). Complete performance metrics were: AUROC: 0.755 ± 0.191, accuracy: 0.667 ± 0.170, precision: 0.738 ± 0.263, recall: 0.564 ± 0.218, F1 score: 0.617 ± 0.206. For details, see Supplementary Methods.

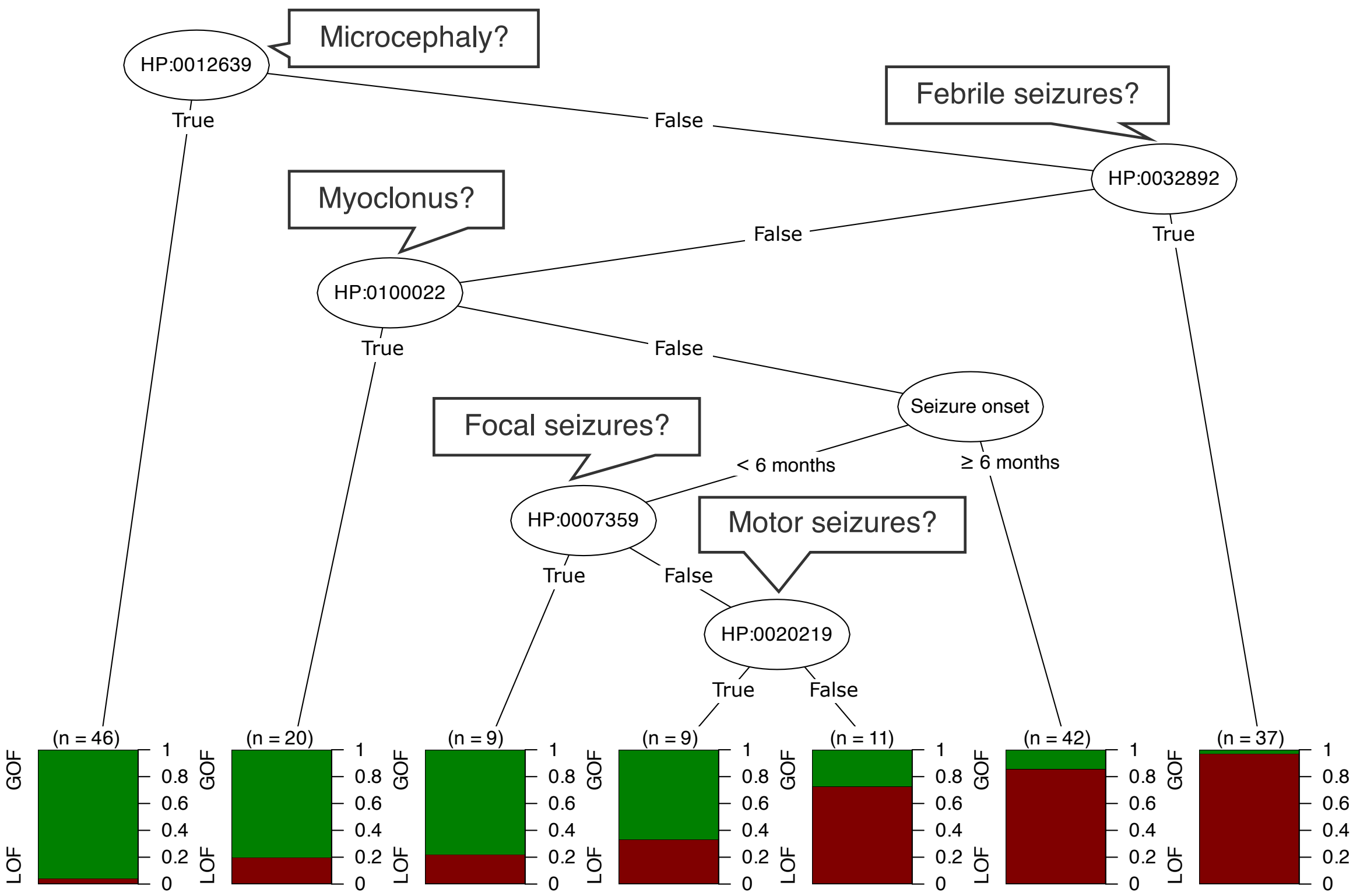

Supplementary Figure 4. Clinical decision tree. We fit a decision tree model using the `rpart` package and the same internal *k*-fold cross-validation procedure. Terms included by the model were HP:0012639: morphological CNS abnormality incl. microcephaly; HP:0032892: infection-related seizure incl. febrile seizure; HP:0100022: abnormality of movement incl. myoclonus; HP:0007359: focal seizure; HP:0020219: motor seizure.

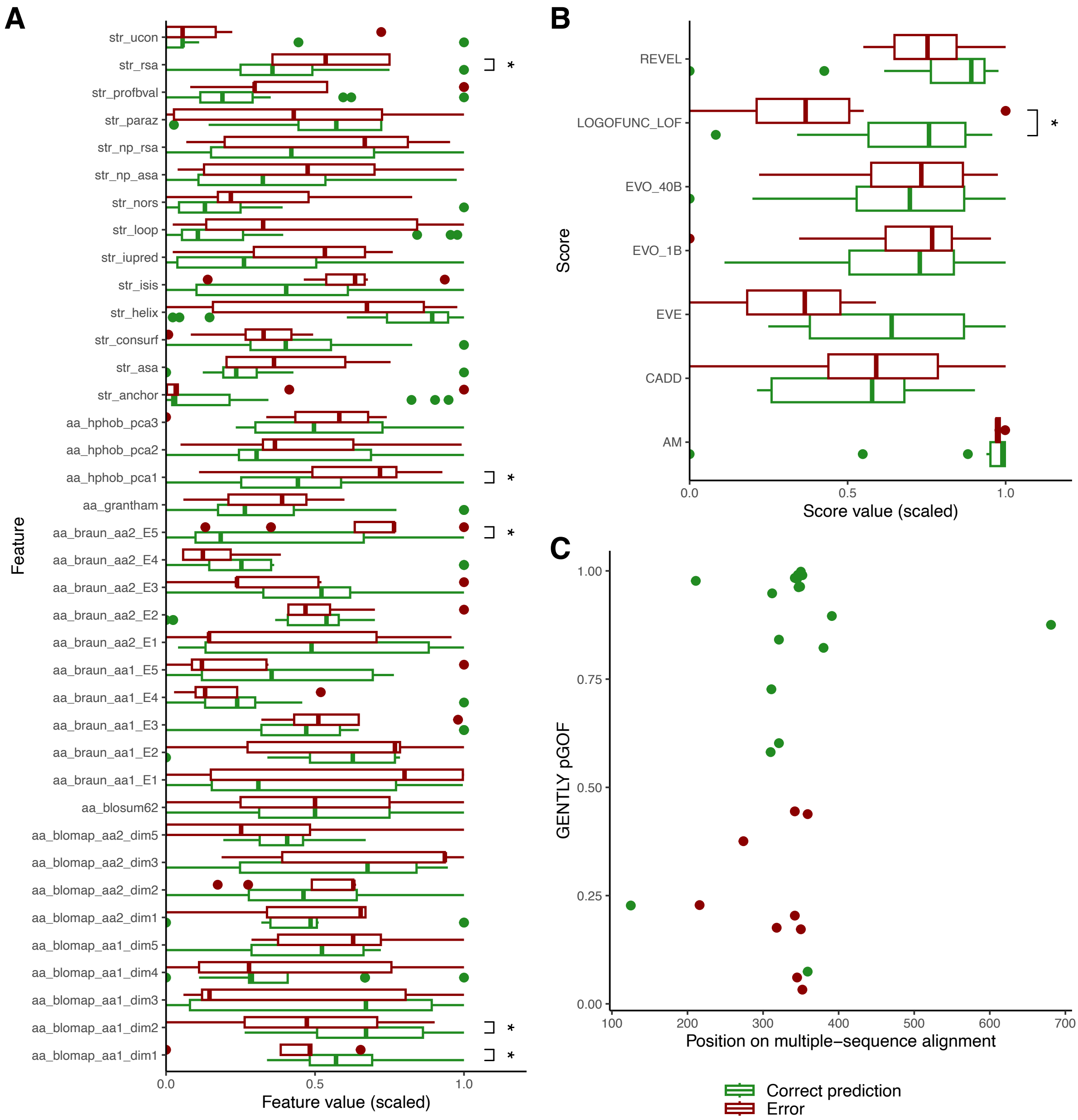

Supplementary Figure 5. Error analysis. Variants from the external validation set were annotated as errors if the full model prediction did not match the observed experimental effect, and as correct predictions otherwise. Variants were then mapped back to the feature dictionary used for kernel construction. For better visual comparison, raw feature and score values are shown after min-max scaling. **(A)** Sequence and structure-based features. **(B)** Pathogenicity and functional prediction scores, for comparison. **(C)** Distribution of errors across the receptor family multiple-sequence alignment. Two-sided Wilcoxon rank-sum test. Significance: *p < 0.05.

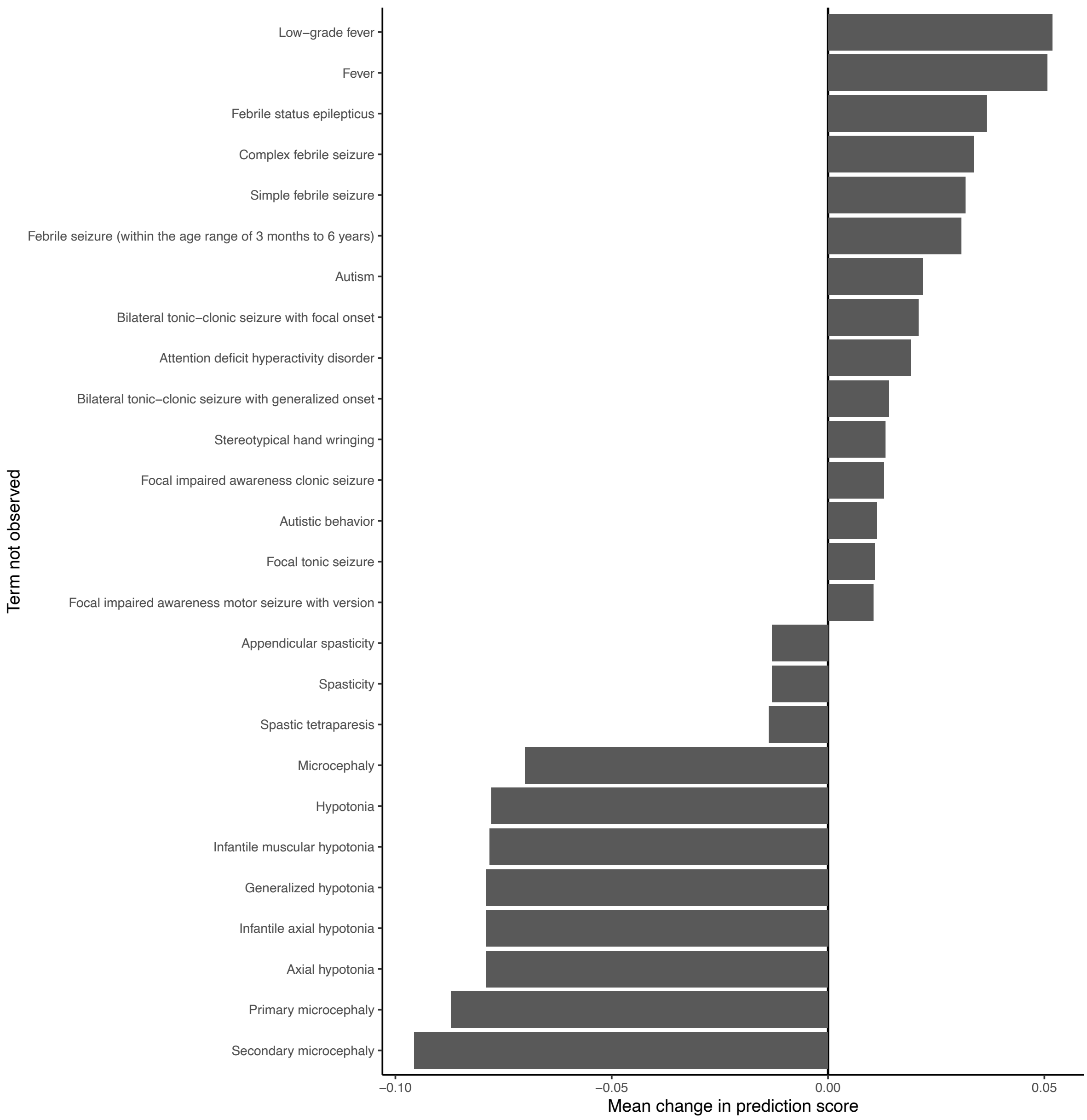

Supplementary Figure 6. Ablation of clinical characteristics to investigate age-related predictive performance. For each clinical characteristic (HPO term), we simulated absence (non-observation, e.g. due to incomplete data or because the phenotype could not manifest yet) by removing the term and all related ancestors from the training dataset and observing how the model prediction changes. A negative mean change in prediction score denotes that removing the phenotype biases the prediction towards loss-of-function (LOF), and vice-versa. Note that the prediction score ranges from -2.39 to 1.74, making any changes seen here comparatively small. Some redundancy (e.g. showing both primary and secondary microcephaly) is expected from the similarity-based ontological reasoning, where similar concepts are grouped.

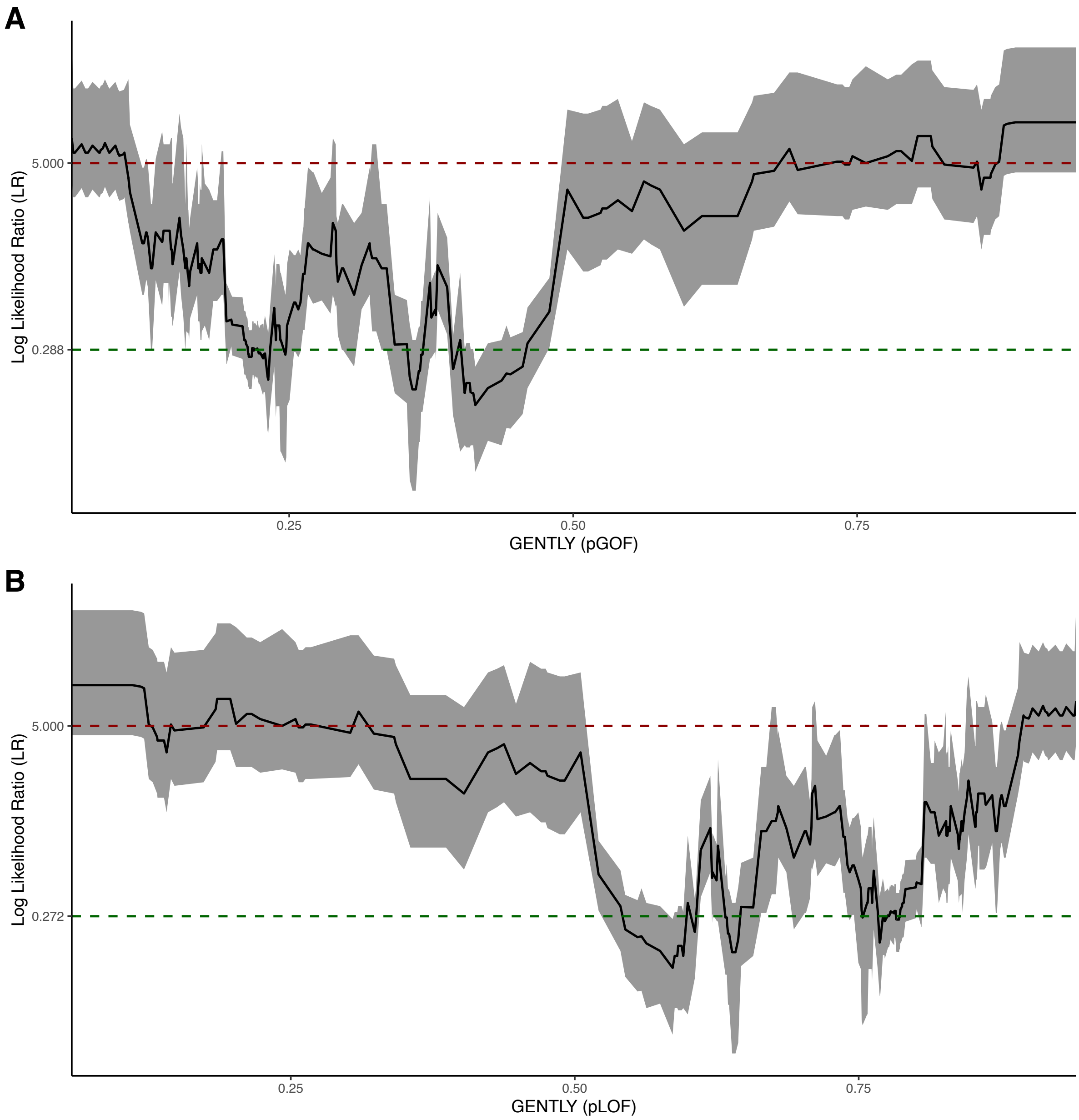

Supplementary Figure 7. Computational evidence calibration according to the ACMG likelihood ratio-based framework. (A-B) Likelihood ratios are shown separately for each class probability (pGOF, pLOF). Note that unlike pathogenicity prediction algorithms, functional prediction algorithms are bi-directional, with low pLOF corresponding to high pGOF and vice versa. Likelihood ratio estimates are shown as lines, with shaded areas denoting the lower and upper bounds. Dashed lines denote the lower bound for supporting evidence of pathogenicity (PP3_Supporting) and the upper bound for supporting evidence of benign variation (BP4_Supporting), respectively.

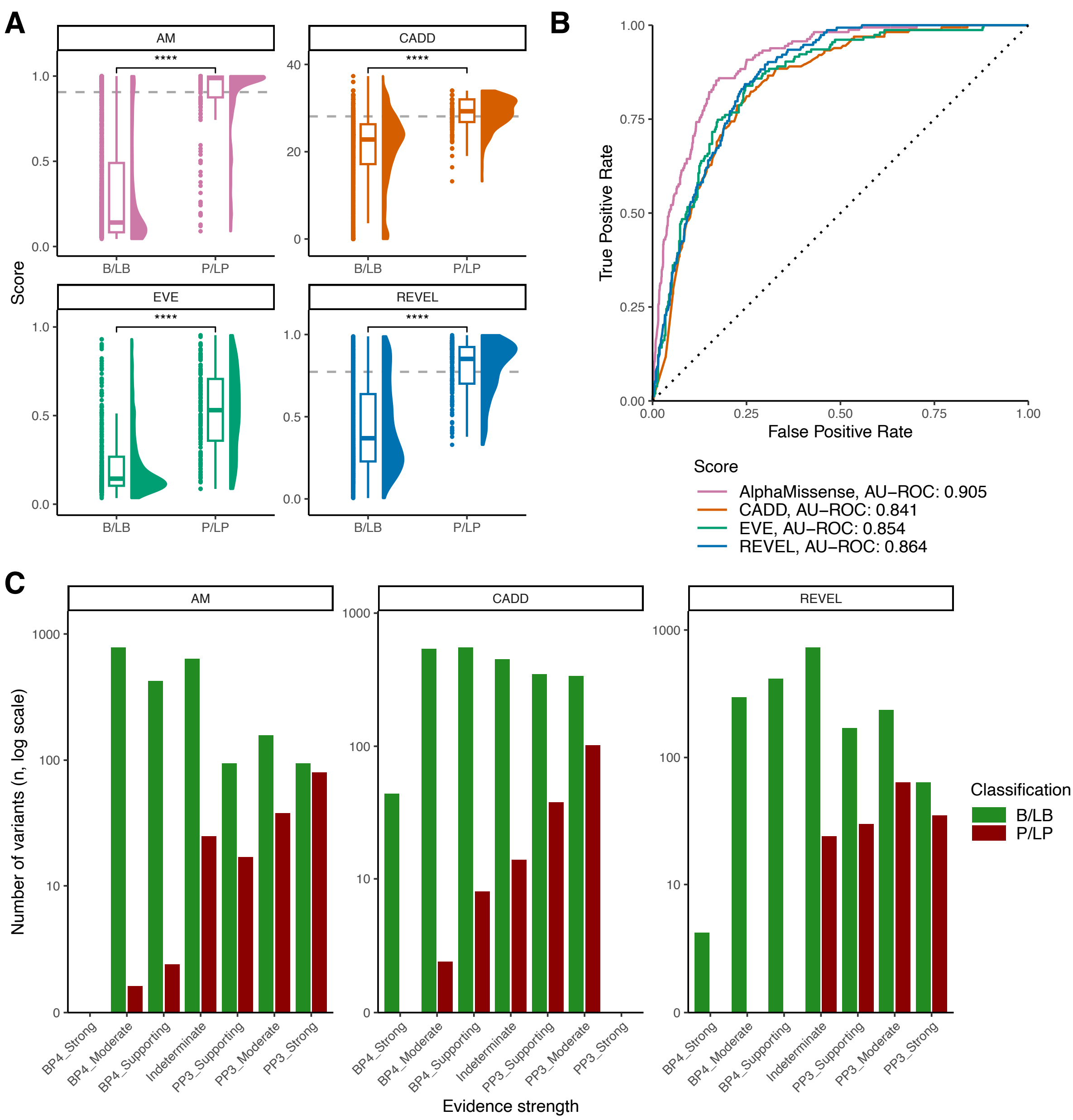

Supplementary Figure 8. Performance of pathogenicity prediction models on variants in genes encoding GABA$_A$ receptor subunits. (Likely) pathogenic variants (P/LP, n = 174) were variants in our training dataset. (Likely) benign variants (B/LB, n = 2,591 variants) were obtained from population databases under an arbitrary AC threshold (AC>3). **(A)** Four common pathogenicity prediction models (AlphaMissense, AM; CADD; EVE; REVEL) all separate between P/LP and B/LB variants. Two-sided t-test. Significance: ****p<0.0001. Dashed lines show score thresholds above which scores can be used to assign the ACMG criterion PP3_Moderate. **(B)** ROC curves for each pathogenicity prediction model. **(C)** Distribution of P/LP and B/LB variants according to calibrated ACMG evidence strengths for each score.

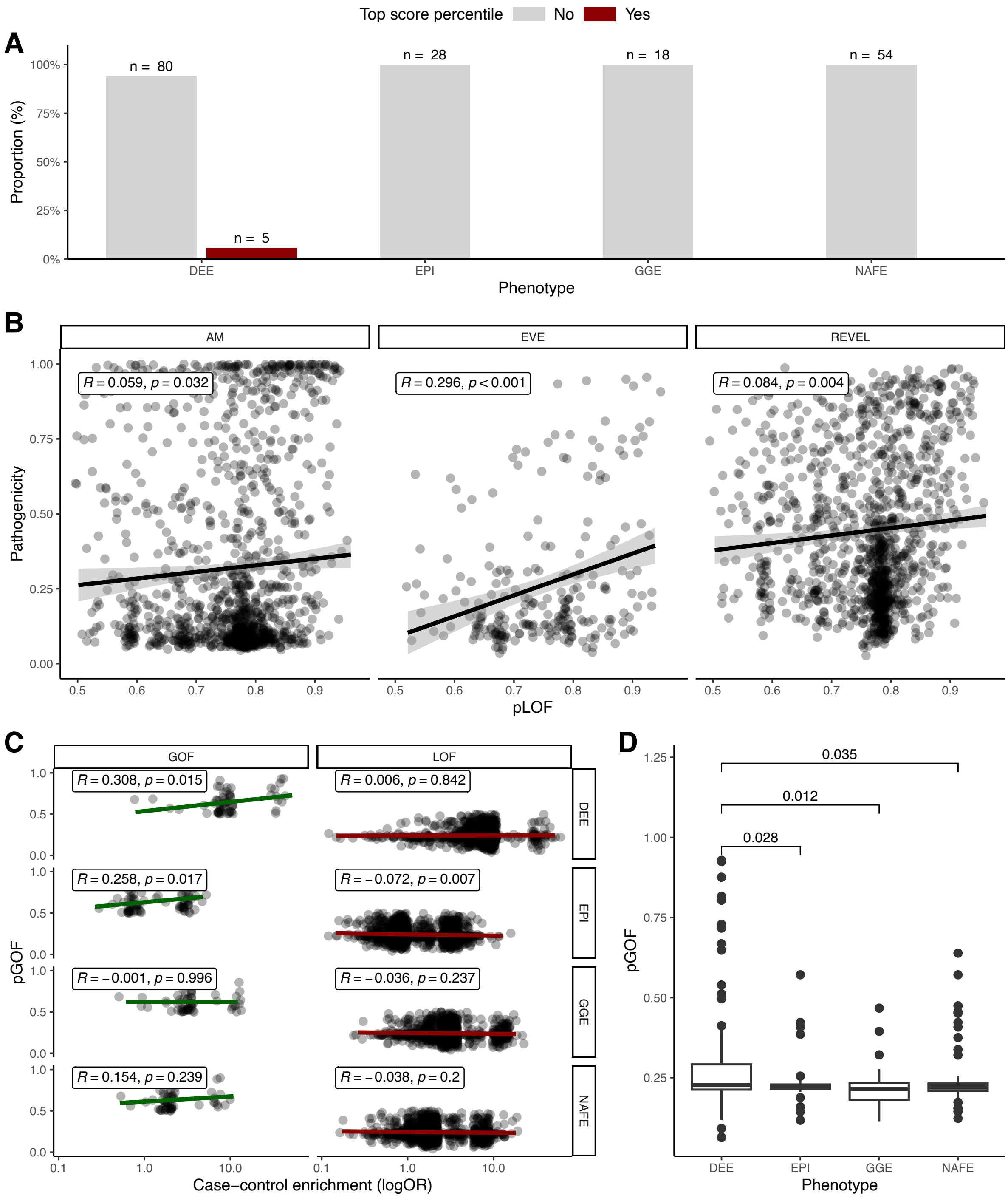

Supplementary Figure 9. Functional effect predictions across GABA$_A$ receptor subunit missense variants from 20,979 individuals with epilepsy. **(A)** Variants with enrichment in cases versus controls (Fisher's exact test, $p < 0.05$) are shown across phenotypic subgroups. Note that variants within the top score percentile were only observed in the DEE group. **(B)** Functional effect predictions correlate with pathogenicity predictions across three pathogenicity prediction models. Pearson's correlation coefficient. **(C)** Model predictions, shown separately across predicted class (GOF vs. LOF) and phenotypic subgroups, correlate with case-control enrichment. Pearson's correlation coefficient. **(D)** Across variants with enriched in cases versus controls, predictions can separate across phenotypic subgroups (two-sided t-test). Abbreviations: DEE – developmental and epileptic encephalopathy; EPI – epilepsy; GGE – genetic generalized epilepsy; NAFE – non-acquired focal epilepsy.

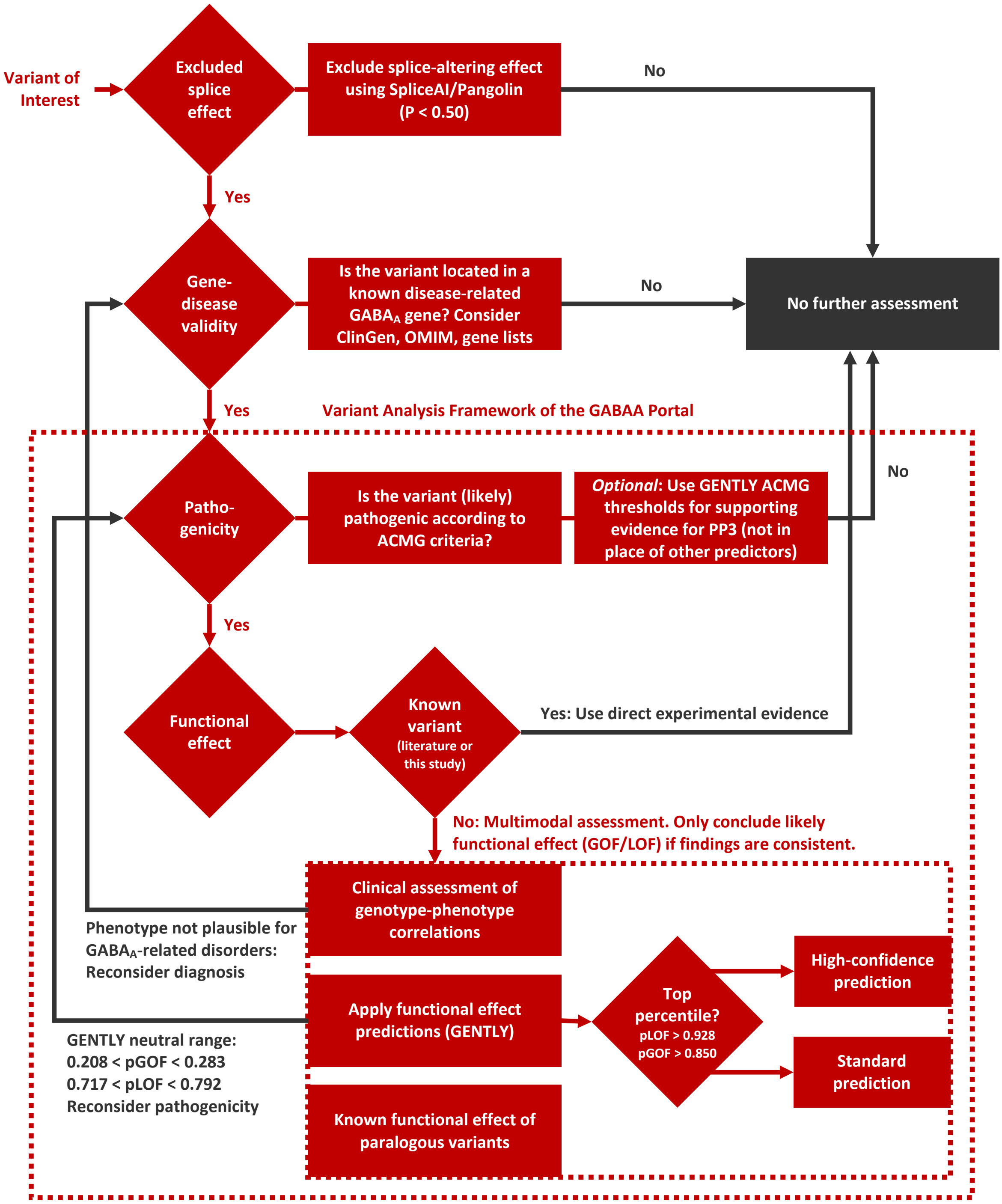

Supplementary Figure 10. Proposed workflow for variant assessment. Given a missense variant of interest in any of the genes encoding for GABA$_A$ receptor subunits, optionally first exclude a possible splice-altering effect using SpliceAI/Pangolin (standard threshold, P > 0.50).[6] Next, establish gene-disease validity using ClinGen curations, alternatively OMIM curations or epilepsy gene lists.[9–11] If gene-disease validity is established, interpret variant pathogenicity using the standards and guidelines of the American College of Medical Genetics and Genomics (ACMG).[12] Here, GENTLY scores may optionally be used as supporting evidence for criterion PP3 given the provided likelihood-ratio calibrated score ranges. However, preference should be given to established pathogenicity predictors that can provide stronger PP3 evidence, e.g. REVEL.[2,13] If the variant can be classified as (likely) pathogenic, check if the variant has already been functionally assessed in literature, including this study. If

so, use direct experimental evidence of function. If not, assess: (i) Phenotype, using gene-specific genotype-phenotype-function correlations from cohort studies.[14] If the phenotype is not plausible, reconsider if there is another possible aetiology; *(ii)* In silico predictions. Variants within the top score percentiles can be considered with high confidence, while variants within the neutral score ranges shown should prompt reassessment of pathogenicity; *(iii)* Known effects of paralogous variants in other related genes (e.g. from PER Viewer or Gene Portals).[1,3] Only if all three components of the assessment are concordant should a likely functional effect (GOF/LOF) be concluded. Otherwise, the overall functional effect remains uncertain until experimental evidence can be obtained.

UniProt accession	UniProt name	Gene name (HUGO)	Transcript ID (MANE)	ClinGen: Gene-disease validity	OMIM: Associations (Inheritance)
**O00591**	GBRP_HUMAN	*GABRP*	ENST00000265294.9	Not curated	Not curated
**O14764**	GBRD_HUMAN	*GABRD*	ENST00000378585.7	Complex neurodevelopmental disorder (moderate); epilepsy (limited)	Susceptibility: Epilepsy (AD)
**P14867**	GBRA1_HUMAN	*GABRA1*	ENST00000635880.1	Epilepsy (definitive)	Established: DEE (AD); Susceptibility: Epilepsy
**P18505**	GBRB1_HUMAN	*GABRB1*	ENST00000295454.8	Developmental and epileptic encephalopathy (limited)	Established: DEE (AD)
**P18507**	GBRG2_HUMAN	*GABRG2*	ENST00000639213.1	Epilepsy (definitive)	Established: DEE, FS, GEFS+ (AD)
**P28472**	GBRB3_HUMAN	*GABRB3*	ENST00000311550.10	Developmental and epileptic encephalopathy (definitive)	Established: DEE (AD); Susceptibility: Epilepsy
**P31644**	GBRA5_HUMAN	*GABRA5*	ENST00000400081.7	Not curated	Established: DEE (AD)
**P34903**	GBRA3_HUMAN	*GABRA3*	ENST00000535043.1	Not curated	Established: EPILX2 (XL)
**P47869**	GBRA2_HUMAN	*GABRA2*	ENST00000356504.5	Not curated	Established: DEE (AD); Susceptibility: Alcohol dependence
**P47870**	GBRB2_HUMAN	*GABRB2*	ENST00000274547.6	Complex neurodevelopmental disorder (definitive)	Established: DEE (AD)
**P48169**	GBRA4_HUMAN	*GABRA4*	ENST00000264318.4	Not curated	Not curated
**P78334**	GBRE_HUMAN	*GABRE*	ENST00000370328.4	Not curated	Not curated
**Q16445**	GBRA6_HUMAN	*GABRA6*	ENST00000274545.10	Not curated	Not curated
**Q8N1C3**	GBRG1_HUMAN	*GABRG1*	ENST00000295452.5	Not curated	Not curated
**Q99928**	GBRG3_HUMAN	*GABRG3*	ENST00000615808.5	Not curated	Not curated
**Q9UN88**	GBRT_HUMAN	*GABRQ*	ENST00000598523.3	Not curated	Not curated

Supplementary Table 1. List of genes included in this study along with their gene-disease associations, accession IDs, and reference transcript IDs. Further information on gene-disease associations was curated from ClinGen and OMIM, last accessed 02/04/2026.[9,10] For each OMIM curation, the mode of inheritance is shown (AD – autosomal-dominant; XL – X-linked). Abbreviations: DEE – developmental and epileptic encephalopathy; EPILX2 – Epilepsy, X-linked 2, with or without impaired intellectual development and dysmorphic features; FS – febrile seizures; GEFS+ – Generalized epilepsy with febrile seizures plus; HUGO – HUGO Gene Nomenclature Committee (HGNC) of the Human Genome Organisation (HUGO); MANE – Matched Annotation from NCBI and EMBL-EBI.

Gene	aa1	pos	aa2	Set	BA1	BS1	PM2	PS1	PM5	PP3	BP4	PM1	PS3	Final Score	Classification
GABRA1	Met	263	Thr	training			Supporting	Strong	Moderate	Moderate		Moderate		11	Pathogenic
GABRB2	Arg	240	Thr	training			Supporting	Strong		Strong				9	Likely-pathogenic
GABRA1	Pro	260	Leu	training			Supporting	Strong	Moderate	Strong		Moderate		13	Pathogenic
GABRA1	Arg	112	Gln	training			Supporting	Strong	Moderate	Moderate			Strong	13	Pathogenic
GABRA1	Met	263	Ile	training			Supporting	Strong	Moderate	Moderate		Moderate		11	Pathogenic
GABRA1	Val	287	Leu	training			Supporting	Strong	Moderate	Supporting		Moderate		10	Pathogenic
GABRG2	Thr	90	Met	training				Strong	Moderate	Supporting				7	Likely-pathogenic
GABRG2	Arg	323	Gln	training			Supporting	Strong	Moderate	Moderate				9	Likely-pathogenic
GABRB3	Gln	210	His	training			Supporting	Strong	Moderate	Moderate			Strong	13	Pathogenic
GABRG2	Ala	106	Thr	training			Supporting	Strong	Moderate				Strong	11	Pathogenic
GABRB3	Phe	318	Ser	training			Supporting	Strong	Moderate	Strong				11	Pathogenic
GABRB3	Leu	260	Met	training			Supporting	Strong	Moderate	Supporting		Moderate		10	Pathogenic
GABRB3	Ile	257	Met	training			Supporting	Strong	Moderate	Supporting		Moderate		10	Pathogenic
GABRA1	Thr	294	Lys	training			Supporting	Strong	Moderate	Strong		Moderate		13	Pathogenic
GABRA1	Ala	332	Val	training			Supporting	Strong	Moderate	Moderate				9	Likely-pathogenic
GABRA1	Arg	214	Cys	training			Supporting	Strong	Moderate	Supporting				8	Likely-pathogenic
GABRB3	Asn	328	Asp	training			Supporting	Strong	Moderate	Moderate			Strong	13	Pathogenic
GABRB3	Glu	357	Lys	training		Strong		Strong						0	Uncertain
GABRA1	Val	74	Ile	training				Strong	Moderate					6	Likely-pathogenic
GABRA1	Ser	76	Arg	training			Supporting	Strong	Moderate	Strong				11	Pathogenic
GABRA1	Phe	104	Cys	training			Supporting	Strong	Moderate	Strong				11	Pathogenic

GABRA1	Asn	115	Asp	training			Supporting	Strong	Moderate	Supporting				8	Likely-pathogenic
GABRA1	Leu	146	Met	training			Supporting	Strong						5	Uncertain
GABRA1	Arg	214	His	training			Supporting	Strong	Moderate					7	Likely-pathogenic
GABRA1	Gly	251	Asp	training			Supporting	Strong	Moderate	Strong				11	Pathogenic
GABRA1	Thr	289	Pro	training			Supporting	Strong	Moderate	Strong		Moderate		13	Pathogenic
GABRA1	Lys	306	Thr	training			Supporting	Strong	Moderate	Strong		Moderate		13	Pathogenic
GABRA1	Leu	215	Val	training			Supporting	Strong	Moderate	Supporting				8	Likely-pathogenic
GABRB3	Glu	180	Gly	training			Supporting	Strong		Moderate			Strong	11	Pathogenic
GABRB3	Asp	120	Asn	training			Supporting	Strong	Moderate	Moderate			Strong	13	Pathogenic
GABRB3	Tyr	302	Cys	training			Supporting	Strong	Moderate	Strong		Moderate	Strong	17	Pathogenic
GABRA1	Gly	251	Ser	training			Supporting	Strong	Moderate	Strong				11	Pathogenic
GABRA1	Thr	43	Ile	training			Supporting	Strong	Moderate	Moderate				9	Likely-pathogenic
GABRA1	Glu	63	Lys	training			Supporting	Strong	Moderate					7	Likely-pathogenic
GABRA1	Ser	96	Cys	training			Supporting	Strong	Moderate	Supporting				8	Likely-pathogenic
GABRA1	Ser	213	Thr	training			Supporting	Strong		Supporting				6	Likely-pathogenic
GABRA1	Val	270	Ala	training			Supporting	Strong	Moderate	Strong				11	Pathogenic
GABRA1	Asn	275	Lys	training			Supporting	Strong	Moderate					7	Likely-pathogenic
GABRA1	Thr	295	Ile	training			Supporting	Strong	Moderate	Moderate		Moderate		11	Pathogenic
GABRA1	Ser	299	Asn	training			Supporting	Strong	Moderate			Moderate		9	Likely-pathogenic
GABRA1	Pro	305	Leu	training			Supporting	Strong	Moderate	Strong				11	Pathogenic
GABRA1	Ile	317	Leu	training			Supporting	Strong	Moderate					7	Likely-pathogenic
GABRA1	Ala	332	Thr	training			Supporting	Strong	Moderate	Moderate				9	Likely-pathogenic
GABRA1	Arg	112	Trp	training			Supporting	Strong	Moderate	Supporting				8	Likely-pathogenic

GABRA1	Asn	114	Asp	training			Supporting	Strong						5	Uncertain
GABRA1	Tyr	189	Phe	training			Supporting	Strong		Moderate				7	Likely-pathogenic
GABRA1	Pro	260	Ser	training			Supporting	Strong	Moderate	Strong		Moderate		13	Pathogenic
GABRA1	Pro	181	Ser	training			Supporting	Strong	Moderate	Supporting				8	Likely-pathogenic
GABRB3	Ser	254	Phe	training			Supporting	Strong	Moderate	Moderate		Moderate	Strong	15	Pathogenic
GABRA1	Asp	219	Asn	training			Supporting	Strong	Moderate					7	Likely-pathogenic
GABRG2	Pro	83	Ser	training				Strong	Moderate	Strong				10	Pathogenic
GABRA1	Thr	292	Ile	training			Supporting	Strong	Moderate	Strong		Moderate		13	Pathogenic
GABRA1	Ala	322	Asp	training			Supporting	Strong	Moderate	Moderate				9	Likely-pathogenic
GABRA1	Pro	280	Leu	training			Supporting	Strong	Moderate	Moderate		Moderate		11	Pathogenic
GABRA1	Leu	267	Ile	training			Supporting	Strong	Moderate	Supporting		Moderate		10	Pathogenic
GABRA1	Leu	267	Val	training			Supporting	Strong	Moderate	Moderate		Moderate		11	Pathogenic
GABRA1	Thr	289	Ser	training			Supporting	Strong	Moderate	Moderate		Moderate		11	Pathogenic
GABRB3	Lys	127	Arg	training			Supporting	Strong	Moderate	Supporting			Strong	12	Pathogenic
GABRG2	Arg	82	Gln	training				Strong	Moderate	Moderate				8	Likely-pathogenic
GABRG2	Arg	177	Gly	training			Supporting	Strong	Moderate	Supporting				8	Likely-pathogenic
GABRG2	Asn	79	Ser	training		Strong		Strong	Moderate					2	Uncertain
GABRG2	Gly	257	Arg	training				Strong	Moderate	Moderate				8	Likely-pathogenic
GABRG2	Ile	107	Thr	training			Supporting	Strong						5	Uncertain
GABRG2	Pro	282	Ser	training			Supporting	Strong	Moderate	Strong		Moderate		13	Pathogenic
GABRG2	Arg	323	Trp	training			Supporting	Strong	Moderate	Moderate				9	Likely-pathogenic
GABRG2	Phe	343	Leu	training			Supporting	Strong		Moderate				7	Likely-pathogenic
GABRG2	Met	199	Val	training				Strong	Moderate	Moderate				8	Likely-pathogenic

GABRG2	Ile	218	Ser	training				Strong	Moderate	Moderate				8	Likely-pathogenic
GABRG2	Pro	282	Thr	training			Supporting	Strong	Moderate	Strong		Moderate		13	Pathogenic
GABRG2	Ser	306	Phe	training			Supporting	Strong	Moderate	Moderate		Moderate		11	Pathogenic
GABRG2	Leu	313	Gly	training											
GABRG2	Arg	383	Cys	training				Strong						4	Uncertain
GABRG2	Ser	325	Leu	training			Supporting	Strong		Supporting				6	Likely-pathogenic
GABRG2	Val	459	Phe	training			Supporting	Strong		Moderate				7	Likely-pathogenic
GABRG2	Pro	302	Leu	training			Supporting	Strong	Moderate	Moderate		Moderate		11	Pathogenic
GABRB3	Val	37	Gly	training			Supporting	Strong		Moderate				7	Likely-pathogenic
GABRB3	Pro	301	Leu	training			Supporting	Strong	Moderate	Moderate		Moderate	Strong	15	Pathogenic
GABRB3	Arg	232	Gln	training			Supporting	Strong	Moderate	Moderate			Strong	13	Pathogenic
GABRB3	Ser	76	Cys	training			Supporting	Strong	Moderate	Moderate			Strong	13	Pathogenic
GABRB3	Arg	142	Leu	training			Supporting	Strong	Moderate	Moderate				9	Likely-pathogenic
GABRB3	Tyr	184	His	training			Supporting	Strong	Moderate	Strong			Strong	15	Pathogenic
GABRB3	Thr	157	Met	training				Strong		Supporting			Strong	9	Likely-pathogenic
GABRB3	Leu	256	Gln	training			Supporting	Strong	Moderate	Moderate		Moderate	Strong	15	Pathogenic
GABRB3	Leu	124	Phe	training			Supporting	Strong	Moderate				Strong	11	Pathogenic
GABRB3	Thr	185	Ile	training			Supporting	Strong	Moderate	Moderate			Strong	13	Pathogenic
GABRB3	Pro	253	Leu	training			Supporting	Strong	Moderate	Strong		Moderate	Strong	17	Pathogenic
GABRB3	Glu	77	Lys	training			Supporting	Strong	Moderate	Moderate			Strong	13	Pathogenic
GABRB3	Val	78	Phe	training			Supporting	Strong	Moderate	Moderate			Strong	13	Pathogenic
GABRB3	Met	80	Lys	training			Supporting	Strong	Moderate	Moderate			Strong	13	Pathogenic
GABRB3	Thr	287	Ile	training			Supporting	Strong	Moderate	Strong		Moderate	Strong	17	Pathogenic

GABRB3	Asn	110	Asp	training			Supporting	Strong	Moderate	Moderate			Strong	13	Pathogenic
GABRB3	Leu	284	Met	training			Supporting	Strong	Moderate	Moderate		Moderate	Strong	15	Pathogenic
GABRB3	Leu	165	Gln	training											
GABRB3	Leu	170	Arg	training			Supporting	Strong		Moderate			Strong	11	Pathogenic
GABRB3	Glu	178	Gly	training			Supporting	Strong	Moderate	Moderate			Strong	13	Pathogenic
GABRB3	Tyr	182	Phe	training			Supporting	Strong		Supporting			Strong	10	Pathogenic
GABRG2	Thr	90	Arg	training			Supporting	Strong	Moderate	Moderate				9	Likely-pathogenic
GABRG2	Pro	83	Thr	training			Supporting	Strong	Moderate	Strong				11	Pathogenic
GABRB3	Phe	225	Cys	training			Supporting	Strong	Moderate	Moderate			Strong	13	Pathogenic
GABRB3	Tyr	230	His	training			Supporting	Strong		Moderate			Strong	11	Pathogenic
GABRB2	Asp	125	Asn	training			Supporting	Strong	Moderate				Strong	11	Pathogenic
GABRB2	Arg	293	Pro	training			Supporting	Strong	Moderate	Strong			Strong	15	Pathogenic
GABRA1	Val	162	Met	training			Supporting	Strong	Moderate	Supporting				8	Likely-pathogenic
GABRA1	Arg	147	Gln	training				Strong	Moderate	Moderate				8	Likely-pathogenic
GABRB3	Leu	310	Ile	training			Supporting	Strong	Moderate	Supporting				8	Likely-pathogenic
GABRB3	Pro	54	Leu	training			Supporting	Strong		Strong				9	Likely-pathogenic
GABRB3	Arg	217	Cys	training				Strong						4	Uncertain
GABRB3	Arg	232	Pro	training			Supporting	Strong	Moderate	Moderate			Strong	13	Pathogenic
GABRA1	Arg	204	Cys	training				Strong						4	Uncertain
GABRB3	Gln	249	Lys	training			Supporting	Strong	Moderate	Moderate		Moderate	Strong	15	Pathogenic
GABRB3	Thr	281	Ala	training			Supporting	Strong	Moderate	Strong		Moderate	Strong	17	Pathogenic
GABRA1	Tyr	438	Cys	training			Supporting	Strong		Strong				9	Likely-pathogenic
GABRB3	Thr	281	Ile	training			Supporting	Strong	Moderate	Strong		Moderate	Strong	17	Pathogenic

GABRB3	Leu	284	Arg	training			Supporting	Strong	Moderate	Strong		Moderate	Strong	17	Pathogenic
GABRB3	Leu	284	Pro	training			Supporting	Strong	Moderate	Strong		Moderate	Strong	17	Pathogenic
GABRB3	Thr	288	Asn	training			Supporting	Strong	Moderate	Moderate		Moderate	Strong	15	Pathogenic
GABRB3	Leu	293	His	training			Supporting	Strong	Moderate	Moderate			Strong	13	Pathogenic
GABRB3	Ile	300	Thr	training			Supporting	Strong	Moderate	Moderate		Moderate	Strong	15	Pathogenic
GABRB3	Ala	305	Thr	training			Supporting	Strong	Moderate	Strong		Moderate	Strong	17	Pathogenic
GABRB3	Ala	305	Val	training			Supporting	Strong	Moderate	Strong		Moderate	Strong	17	Pathogenic
GABRB3	Ser	433	Leu	training			Supporting	Strong						5	Uncertain
GABRB3	Pro	253	Ser	training			Supporting	Strong	Moderate	Strong		Moderate	Strong	17	Pathogenic
GABRB3	Met	311	Thr	training			Supporting	Strong	Moderate	Supporting				8	Likely-pathogenic
GABRB3	Ile	280	Phe	training			Supporting	Strong	Moderate	Strong		Moderate		13	Pathogenic
GABRA1	Glu	403	Gln	training				Strong	Moderate		Supporting			5	Uncertain
GABRB3	Met	80	Thr	training			Supporting	Strong	Moderate	Moderate				9	Likely-pathogenic
GABRB3	Tyr	245	His	training			Supporting	Strong	Moderate	Moderate			Strong	13	Pathogenic
GABRB2	Tyr	244	His	training			Supporting	Strong	Moderate	Moderate			Strong	13	Pathogenic
GABRB2	Leu	277	Ser	training			Supporting	Strong	Moderate	Strong		Moderate		13	Pathogenic
GABRB2	Thr	284	Lys	training			Supporting	Strong	Moderate	Strong		Moderate	Strong	17	Pathogenic
GABRB2	Val	316	Ile	training				Strong	Moderate	Moderate			Strong	12	Pathogenic
GABRB2	Ala	159	Ser	training			Supporting	Strong					Strong	9	Likely-pathogenic
GABRB2	Met	161	Leu	training			Supporting	Strong	Moderate	Supporting			Strong	12	Pathogenic
GABRB2	Tyr	181	Phe	training			Supporting	Strong	Moderate	Supporting			Strong	12	Pathogenic
GABRB2	Tyr	183	His	training			Supporting	Strong	Moderate	Strong			Strong	15	Pathogenic
GABRB2	Phe	245	Leu	training			Supporting	Strong	Moderate	Moderate			Strong	13	Pathogenic

GABRB2	Ile	246	Thr	training			Supporting	Strong	Moderate	Moderate			Strong	13	Pathogenic
GABRB2	Pro	252	Ala	training			Supporting	Strong	Moderate	Moderate		Moderate	Strong	15	Pathogenic
GABRB2	Pro	252	Leu	training			Supporting	Strong	Moderate	Moderate		Moderate	Strong	15	Pathogenic
GABRB2	Leu	255	Val	training			Supporting	Strong	Moderate	Moderate		Moderate	Strong	15	Pathogenic
GABRG2	Tyr	274	Cys	training			Supporting	Strong		Strong				9	Likely-pathogenic
GABRB2	Val	282	Ala	training			Supporting	Strong	Moderate	Moderate		Moderate	Strong	15	Pathogenic
GABRB2	Ile	288	Ser	training			Supporting	Strong	Moderate	Strong		Moderate		13	Pathogenic
GABRB2	Ile	288	Thr	training			Supporting	Strong	Moderate	Strong		Moderate	Strong	17	Pathogenic
GABRB2	Arg	293	Trp	training				Strong	Moderate	Moderate				8	Likely-pathogenic
GABRB2	Ile	299	Leu	training			Supporting	Strong	Moderate	Moderate		Moderate	Strong	15	Pathogenic
GABRB2	Ile	299	Ser	training			Supporting	Strong	Moderate	Strong		Moderate	Strong	17	Pathogenic
GABRB2	Pro	300	Leu	training			Supporting	Strong	Moderate	Moderate		Moderate	Strong	15	Pathogenic
GABRB2	Val	302	Met	training			Supporting	Strong	Moderate	Moderate		Moderate	Strong	15	Pathogenic
GABRA1	Tyr	252	His	training			Supporting	Strong	Moderate	Strong				11	Pathogenic
GABRB2	Lys	303	Arg	training			Supporting	Strong	Moderate	Moderate		Moderate	Strong	15	Pathogenic
GABRB2	Ala	304	Thr	training			Supporting	Strong	Moderate	Strong		Moderate		13	Pathogenic
GABRB2	Asp	108	Tyr	training			Supporting	Strong		Moderate				7	Likely-pathogenic
GABRB2	Ala	112	Glu	training			Supporting	Strong	Moderate	Moderate				9	Likely-pathogenic
GABRB2	Val	133	Met	training			Supporting	Strong		Supporting				6	Likely-pathogenic
GABRB2	Thr	184	Ile	training			Supporting	Strong	Moderate	Moderate				9	Likely-pathogenic
GABRB2	Phe	224	Cys	training			Supporting	Strong	Moderate	Moderate				9	Likely-pathogenic
GABRB2	Phe	245	Ser	training			Supporting	Strong	Moderate	Strong				11	Pathogenic
GABRB2	Lys	298	Gly	training											

GABRB2	Lys	303	Asn	training			Supporting	Strong	Moderate	Supporting		Moderate	Strong	14	Pathogenic
GABRB2	Phe	331	Ser	training				Strong	Moderate	Supporting				7	Likely-pathogenic
GABRB2	Leu	17	Ser	training			Supporting	Strong						5	Uncertain
GABRB2	Gln	172	His	training			Supporting	Strong		Supporting			Strong	10	Pathogenic
GABRB2	Tyr	301	Cys	training			Supporting	Strong	Moderate	Strong		Moderate	Strong	17	Pathogenic
GABRA1	Tyr	252	Cys	training			Supporting	Strong	Moderate	Moderate				9	Likely-pathogenic
GABRA1	Leu	215	Pro	training			Supporting	Strong	Moderate	Strong				11	Pathogenic
GABRA1	Val	287	Ile	training			Supporting	Strong	Moderate			Moderate		9	Likely-pathogenic
GABRA1	Pro	280	Gln	training			Supporting	Strong	Moderate	Moderate		Moderate		11	Pathogenic
GABRA1	Arg	94	His	training			Supporting	Strong	Moderate	Moderate				9	Likely-pathogenic
GABRA1	Lys	364	Thr	training				Strong						4	Uncertain
GABRB3	Gln	210	Glu	training			Supporting	Strong	Moderate	Supporting				8	Likely-pathogenic
GABRB2	Thr	249	Ala	training			Supporting	Strong	Moderate	Moderate		Moderate		11	Pathogenic
GABRB3	Arg	360	Trp	training		Strong		Strong						0	Uncertain
GABRA1	Leu	145	Phe	training			Supporting	Strong	Moderate					7	Likely-pathogenic
GABRB2	Asn	289	His	training			Supporting	Strong	Moderate	Supporting		Moderate		10	Pathogenic
GABRB3	Val	41	Met	training				Strong						4	Uncertain
GABRA1	Val	287	Gly	training			Supporting	Strong	Moderate	Strong		Moderate		13	Pathogenic
GABRB3	Leu	52	Val	training			Supporting	Strong	Moderate				Strong	11	Pathogenic
GABRB3	Val	78	Leu	training			Supporting	Strong	Moderate				Strong	11	Pathogenic
GABRB3	Met	80	Leu	training			Supporting	Strong	Moderate	Supporting			Strong	12	Pathogenic
GABRB3	Met	163	Val	training			Supporting	Strong	Moderate	Moderate				9	Likely-pathogenic
GABRB3	Gln	249	His	training			Supporting	Strong	Moderate	Supporting		Moderate	Strong	14	Pathogenic

GABRB3	Leu	278	Phe	training			Supporting	Strong	Moderate	Supporting		Moderate	Strong	14	Pathogenic
GABRB3	Thr	288	Ile	training			Supporting	Strong	Moderate	Moderate		Moderate		11	Pathogenic
GABRB3	Leu	321	Pro	training			Supporting	Strong	Moderate	Moderate				9	Likely-pathogenic
GABRB3	Leu	278	Pro	training			Supporting	Strong	Moderate	Strong		Moderate		13	Pathogenic
GABRG2	Ile	85	Lys	training				Strong	Moderate	Moderate				8	Likely-pathogenic
GABRB3	Lys	304	Arg	training			Supporting	Strong	Moderate	Moderate		Moderate		11	Pathogenic
GABRA1	Asn	216	Ile	training			Supporting	Strong		Supporting				6	Likely-pathogenic
GABRA1	Lys	306	Asn	training			Supporting	Strong	Moderate	Supporting		Moderate		10	Pathogenic
GABRA1	Ala	281	Val	training			Supporting	Strong	Moderate	Moderate		Moderate		11	Pathogenic
GABRB2	Met	79	Thr	training			Supporting	Strong	Moderate	Strong			Strong	15	Pathogenic
GABRA1	Thr	292	Ser	training			Supporting	Strong	Moderate	Moderate		Moderate		11	Pathogenic
GABRA1	Phe	92	Leu	training			Supporting	Strong	Moderate	Moderate				9	Likely-pathogenic
GABRA1	Met	263	Val	training			Supporting	Strong	Moderate	Moderate		Moderate		11	Pathogenic
GABRA1	Val	284	Ala	training			Supporting	Strong	Moderate	Supporting		Moderate		10	Pathogenic
GABRA1	Leu	296	Phe	training			Supporting	Strong	Moderate	Supporting		Moderate		10	Pathogenic
GABRA1	Thr	257	Arg	training			Supporting	Strong	Moderate	Moderate		Moderate		11	Pathogenic
GABRA1	Gln	32	Lys	training		Strong		Strong			Supporting			-1	Likely-benign
GABRA1	Thr	289	Ala	training			Supporting	Strong	Moderate	Moderate		Moderate		11	Pathogenic
GABRG2	Ala	322	Asp	training			Supporting	Strong	Moderate	Moderate				9	Likely-pathogenic
GABRG2	Ala	322	Thr	training			Supporting	Strong	Moderate	Supporting				8	Likely-pathogenic
GABRG2	Leu	326	His	training			Supporting	Strong		Moderate				7	Likely-pathogenic
GABRG2	Ile	344	Leu	training			Supporting	Strong	Moderate		Supporting			6	Likely-pathogenic
GABRG2	Ser	139	Asn	training			Supporting	Strong						5	Uncertain

GABRG2	Arg	125	Cys	training				Strong	Moderate	Moderate				8	Likely-pathogenic
GABRG2	Asn	457	Ser	training		Strong		Strong		Moderate				2	Uncertain
GABRG2	Met	169	Thr	training			Supporting	Strong		Moderate				7	Likely-pathogenic
GABRG2	Gly	405	Ser	training				Strong						4	Uncertain
GABRG2	Val	104	Gly	training			Supporting	Strong	Moderate	Moderate				9	Likely-pathogenic
GABRG2	Arg	183	Gln	training											
GABRG2	Pro	282	Ala	training			Supporting	Strong	Moderate	Strong		Moderate		13	Pathogenic
GABRG2	Tyr	331	Cys	training				Strong	Moderate	Strong		Moderate		12	Pathogenic
GABRA1	Ser	299	Arg	training			Supporting	Strong	Moderate	Moderate		Moderate		11	Pathogenic
GABRB2	Ala	304	Val	training			Supporting	Strong	Moderate	Strong		Moderate	Strong	17	Pathogenic
GABRB2	Leu	247	Arg	training			Supporting	Strong	Moderate	Moderate			Strong	13	Pathogenic
GABRB3	Thr	285	Pro	training			Supporting	Strong	Moderate	Strong		Moderate		13	Pathogenic
GABRB2	Ala	272	Gly	training			Supporting	Strong	Moderate	Supporting		Moderate		10	Pathogenic
GABRB2	Leu	283	Ile	training			Supporting	Strong	Moderate	Moderate		Moderate	Strong	15	Pathogenic
GABRB2	Lys	298	Glu	training			Supporting	Strong	Moderate	Moderate		Moderate		11	Pathogenic
GABRB2	Gln	248	Lys	training			Supporting	Strong	Moderate	Strong		Moderate		13	Pathogenic
GABRB3	Met	162	Ile	training			Supporting	Strong	Moderate	Supporting				8	Likely-pathogenic
GABRG2	Leu	135	Phe	training				Strong		Moderate				6	Likely-pathogenic
GABRB2	Ile	299	Val	training			Supporting	Strong	Moderate			Moderate	Strong	13	Pathogenic
GABRB3	Tyr	122	Cys	training			Supporting	Strong	Moderate	Moderate				9	Likely-pathogenic
GABRA1	Thr	295	Ala	training			Supporting	Strong	Moderate	Moderate		Moderate		11	Pathogenic
GABRA1	Ala	308	Val	training			Supporting	Strong	Moderate	Moderate		Moderate		11	Pathogenic
GABRA1	Tyr	187	Asn	training			Supporting	Strong	Moderate	Moderate				9	Likely-pathogenic

GABRB3	Phe	314	Cys	training			Supporting	Strong	Moderate	Moderate				9	Likely-pathogenic
GABRB3	Phe	246	Val	training			Supporting	Strong	Moderate	Moderate				9	Likely-pathogenic
GABRA1	Ile	148	Phe	training			Supporting	Strong		Moderate				7	Likely-pathogenic
GABRB3	Asn	290	His	training			Supporting	Strong	Moderate	Supporting		Moderate		10	Pathogenic
GABRB3	Leu	248	Pro	training			Supporting	Strong	Moderate	Moderate				9	Likely-pathogenic
GABRB3	Thr	156	Asn	training			Supporting	Strong	Moderate	Moderate				9	Likely-pathogenic
GABRB2	Leu	259	Phe	training			Supporting	Strong		Moderate		Moderate		9	Likely-pathogenic
GABRB3	Ile	289	Thr	training			Supporting	Strong	Moderate	Moderate		Moderate		11	Pathogenic
GABRG2	Thr	316	Asn	training			Supporting	Strong	Moderate	Moderate		Moderate		11	Pathogenic
GABRB3	Ile	306	Thr	training			Supporting	Strong	Moderate	Strong		Moderate	Strong	17	Pathogenic
GABRB2	Thr	286	Ile	training			Supporting	Strong	Moderate	Strong		Moderate		13	Pathogenic
GABRA1	Ala	188	Asp	training			Supporting	Strong		Strong				9	Likely-pathogenic
GABRA1	Val	270	Ile	training			Supporting	Strong	Moderate					7	Likely-pathogenic
GABRG2	Cys	342	Tyr	training			Supporting	Strong	Moderate	Moderate				9	Likely-pathogenic
GABRG2	Ala	334	Thr	training				Strong	Moderate	Moderate		Moderate		10	Pathogenic
GABRG2	Pro	327	Leu	training			Supporting	Strong	Moderate	Strong				11	Pathogenic
GABRA1	Phe	285	Leu	training			Supporting	Strong	Moderate			Moderate		9	Likely-pathogenic
GABRA1	Phe	325	Leu	training			Supporting	Strong		Strong				9	Likely-pathogenic
GABRB3	Thr	250	Asn	training			Supporting	Strong		Supporting		Moderate		8	Likely-pathogenic
GABRB2	Ala	272	Val	training			Supporting	Strong	Moderate	Moderate		Moderate		11	Pathogenic
GABRG2	Leu	285	Ile	training			Supporting	Strong	Moderate			Moderate		9	Likely-pathogenic
GABRA1	Met	158	Val	training			Supporting	Strong		Moderate				7	Likely-pathogenic
GABRA1	His	83	Asn	training			Supporting	Strong	Moderate					7	Likely-pathogenic

GABRA1	Val	207	Phe	training			Supporting	Strong		Supporting				6	Likely-pathogenic
GABRG2	Arg	183	Met	training			Supporting	Strong	Moderate	Moderate				9	Likely-pathogenic
GABRB2	Leu	296	Pro	training			Supporting	Strong	Moderate	Strong				11	Pathogenic
GABRB3	Lys	127	Glu	training			Supporting	Strong	Moderate	Moderate				9	Likely-pathogenic
GABRB3	Arg	294	Pro	training			Supporting	Strong	Moderate	Strong				11	Pathogenic
GABRB3	Ala	273	Thr	training			Supporting	Strong		Supporting		Moderate		8	Likely-pathogenic
GABRG2	Met	315	Val	training			Supporting	Strong		Moderate		Moderate		9	Likely-pathogenic
GABRA1	Val	110	Phe	training			Supporting	Strong	Moderate					7	Likely-pathogenic
GABRG2	Arg	82	Trp	training				Strong	Moderate	Moderate				8	Likely-pathogenic
GABRB3	Met	80	Val	training				Strong	Moderate	Moderate			Strong	12	Pathogenic
GABRB3	Phe	225	Ser	training			Supporting	Strong	Moderate	Supporting			Strong	12	Pathogenic
GABRB2	His	291	Pro	training			Supporting	Strong	Moderate	Strong		Moderate		13	Pathogenic
GABRB3	Val	283	Ala	training			Supporting	Strong	Moderate	Strong		Moderate		13	Pathogenic
GABRB3	Tyr	184	Cys	training			Supporting	Strong	Moderate	Strong				11	Pathogenic
GABRB2	Met	85	Ile	training			Supporting	Strong	Moderate					7	Likely-pathogenic
GABRB3	Arg	142	His	training				Strong	Moderate	Moderate				8	Likely-pathogenic
GABRB2	Thr	249	Arg	training			Supporting	Strong	Moderate	Moderate		Moderate		11	Pathogenic
GABRA1	Leu	215	Ile	training			Supporting	Strong	Moderate					7	Likely-pathogenic
GABRA1	Phe	173	Leu	training			Supporting	Strong		Moderate				7	Likely-pathogenic
GABRB3	Ile	300	Val	training			Supporting	Strong	Moderate			Moderate		9	Likely-pathogenic
GABRG2	Pro	282	His	training			Supporting	Strong	Moderate	Strong		Moderate		13	Pathogenic
GABRA1	Met	263	Lys	testing			Supporting	Strong	Moderate	Strong		Moderate		13	Pathogenic
GABRA1	Pro	280	Ala	testing			Supporting	Strong	Moderate	Supporting		Moderate		10	Pathogenic

GABRA1	Cys	261	Arg	testing			Supporting	Strong		Strong		Moderate		11	Pathogenic
GABRA1	Arg	301	Val	testing											
GABRA2	Met	117	Val	testing			Supporting	Strong						5	Uncertain
GABRA2	Leu	291	Val	testing			Supporting	Strong	Moderate	Moderate		Moderate		11	Pathogenic
GABRA2	Asn	335	His	testing			Supporting	Strong		Supporting				6	Likely-pathogenic
GABRA2	Thr	292	Lys	testing			Supporting	Strong	Moderate	Strong		Moderate		13	Pathogenic
GABRA2	Ala	358	Pro	testing				Strong						4	Uncertain
GABRA2	Val	284	Met	testing			Supporting	Strong	Moderate	Supporting		Moderate		10	Pathogenic
GABRA2	Thr	294	Ile	testing			Supporting	Strong	Moderate	Strong		Moderate		13	Pathogenic
GABRA2	Thr	292	Ala	testing			Supporting	Strong	Moderate	Strong		Moderate		13	Pathogenic
GABRA2	Ala	308	Val	testing			Supporting	Strong	Moderate	Moderate		Moderate		11	Pathogenic
GABRA3	Gln	242	Leu	testing			Supporting	Strong		Moderate			Strong	11	Pathogenic
GABRA3	Tyr	474	Cys	testing			Supporting	Strong		Strong			Strong	13	Pathogenic
GABRA3	Val	309	Ala	testing			Supporting	Strong	Moderate	Supporting		Moderate		10	Pathogenic
GABRA3	Leu	179	Val	testing			Supporting	Strong						5	Uncertain
GABRA3	Tyr	346	Cys	testing			Supporting	Strong		Moderate				7	Likely-pathogenic
GABRA3	Val	309	Leu	testing			Supporting	Strong	Moderate	Supporting		Moderate	Strong	14	Pathogenic
GABRA3	Thr	166	Met	testing				Strong	Moderate					6	Likely-pathogenic
GABRA3	Thr	314	Pro	testing			Supporting	Strong	Moderate	Moderate		Moderate		11	Pathogenic
GABRA3	Tyr	277	Cys	testing			Supporting	Strong	Moderate	Moderate				9	Likely-pathogenic
GABRA3	Val	344	Ile	testing		Strong		Strong	Moderate					2	Uncertain
GABRA3	Leu	316	Phe	testing			Supporting	Strong	Moderate	Strong		Moderate		13	Pathogenic
GABRA5	Val	294	Phe	testing			Supporting	Strong	Moderate	Moderate		Moderate		11	Pathogenic

GABRA5	Ser	413	Phe	testing			Supporting	Strong			Moderate			3	Uncertain
GABRA5	Val	291	Leu	testing			Supporting	Strong	Moderate	Moderate		Moderate		11	Pathogenic
GABRA5	Thr	301	Arg	testing			Supporting	Strong	Moderate	Moderate		Moderate		11	Pathogenic
GABRA5	Thr	95	Ser	testing			Supporting	Strong	Moderate	Moderate				9	Likely-pathogenic
GABRA5	Arg	166	Gly	testing			Supporting	Strong	Moderate	Moderate				9	Likely-pathogenic
GABRA5	Ala	339	Val	testing			Supporting	Strong	Moderate	Moderate				9	Likely-pathogenic
GABRA5	Asn	223	Ile	testing			Supporting	Strong						5	Uncertain
GABRA5	Trp	322	Arg	testing			Supporting	Strong	Moderate	Moderate				9	Likely-pathogenic
GABRA5	Thr	301	Met	testing				Strong	Moderate	Moderate		Moderate		10	Pathogenic
GABRB1	Phe	246	Ser	testing			Supporting	Strong	Moderate	Strong				11	Pathogenic
GABRB1	Ile	247	Thr	testing			Supporting	Strong	Moderate	Supporting				8	Likely-pathogenic
GABRB1	Val	219	Gly	testing				Strong	Moderate					6	Likely-pathogenic
GABRB1	Val	63	Ala	testing			Supporting	Strong	Moderate	Moderate				9	Likely-pathogenic
GABRB1	Thr	285	Arg	testing			Supporting	Strong	Moderate	Strong		Moderate		13	Pathogenic
GABRB1	Thr	281	Ser	testing			Supporting	Strong	Moderate	Moderate		Moderate		11	Pathogenic
GABRB1	Thr	285	Lys	testing			Supporting	Strong	Moderate	Strong		Moderate		13	Pathogenic
GABRB1	His	144	Tyr	testing				Strong		Supporting				5	Uncertain
GABRB1	Val	359	Ile	testing			Supporting	Strong						5	Uncertain
GABRB2	His	291	Asp	testing			Supporting	Strong	Moderate	Moderate		Moderate		11	Pathogenic
GABRB2	Pro	252	Thr	testing			Supporting	Strong	Moderate	Strong		Moderate		13	Pathogenic
GABRB3	Phe	332	Ser	testing			Supporting	Strong	Moderate	Supporting				8	Likely-pathogenic
GABRB3	Thr	285	Ala	testing			Supporting	Strong	Moderate	Strong		Moderate		13	Pathogenic
GABRB3	Tyr	302	Phe	testing			Supporting	Strong	Moderate	Moderate		Moderate		11	Pathogenic

GABRB3	Ile	247	Thr	testing			Supporting	Strong	Moderate	Moderate				9	Likely-pathogenic
GABRD	Pro	257	Leu	testing				Strong	Moderate	Strong		Moderate	Strong	16	Pathogenic
GABRD	Leu	260	Val	testing			Supporting	Strong	Moderate	Moderate		Moderate		11	Pathogenic

Supplementary Table 2. Results of the automated ACMG Classification. For details, see Supplementary Methods.

Variant	Effect	**Pathogenicity prediction** (REVEL/<br>EVE/<br>AM/<br>CADD)	**Functional prediction** (pLOF LoGoFunc/<br>EVO2/<br>pLOF GENTLY)	**SpliceAI** (AG/<br>AL/<br>DG/<br>DL)	**Pangolin** (Splice Loss/<br>Splice Gain)
GABRA1:p.Ser96Cys	Neutral	0.696/<br>0.574/<br>0.314/<br>27.7	0.378/<br>-0.002/<br>0.832	0.13/<br>**0.45**/<br>0/<br>0	**0.21**/<br>0.13
GABRB3:p.Lys52Val	LOF	0.585/<br>0.474/<br>0.905/<br>33	0.347/<br>-0.003/<br>0.928	0/<br>0/<br>**0.54**/<br>0.02	0.02/<br>0.14
GABRA1:p.Phe92Leu	LOF	0.853/<br>0.497/<br>0.999/<br>26.4	0.595/<br>-0.002/<br>0.928	0.17/<br>**0.28**/<br>0/<br>0	0.16/<br>0.17
GABRG2:p.Arg125Cys	LOF	0.839/<br>0.956/<br>0.998/<br>34	0.768/<br>-0.002/<br>0.928	**0.27**/<br>0.08/<br>0/<br>0	0.19/<br>**0.30**

Supplementary Table 3. Annotations for variants above the high-recall threshold of SpliceAI. For each variant, the experimentally determined functional effect, pathogenicity predictions, functional predictions, and splicing predictions (SpliceAI, Pangolin) are shown. Abbreviations: AM – AlphaMissense; CADD – Combined Annotation Dependent Depletion; EVE – evolutionary model of variant effect; GOF – gain-of-function; LOF – loss-of-function; REVEL – Rare Exome Variant Ensemble Learner.

Model	Accuracy	Balanced Accuracy	F1 Score	MCC	AU-PRC	AU-ROC	Sensitivity	Specificity
**Full model**	**0.867 ± 0.060**	**0.867 ± 0.060**	**0.875 ± 0.056**	**0.741 ± 0.117**	**0.946 ± 0.033**	**0.943 ± 0.035**	**0.887 ± 0.082**	**0.846 ± 0.103**
No clinical features	0.789 ± 0.062	0.785 ± 0.062	0.810 ± 0.060	0.585 ± 0.127	0.844 ± 0.062	0.849 ± 0.052	0.871 ± 0.090	0.699 ± 0.075
No sequence features	0.854 ± 0.058	0.853 ± 0.059	0.861 ± 0.057	0.714 ± 0.116	0.948 ± 0.028	0.942 ± 0.032	0.872 ± 0.088	0.833 ± 0.101
No structural features	0.859 ± 0.053	0.858 ± 0.054	0.868 ± 0.048	0.723 ± 0.104	0.929 ± 0.039	0.929 ± 0.039	0.881 ± 0.063	0.836 ± 0.099
No multi-task learning	0.874 ± 0.060	0.874 ± 0.060	0.879 ± 0.059	0.753 ± 0.120	0.947 ± 0.034	0.944 ± 0.038	0.876 ± 0.084	0.872 ± 0.085
No 3D structural features	0.878 ± 0.055	0.877 ± 0.055	0.884 ± 0.054	0.761 ± 0.107	0.945 ± 0.031	0.942 ± 0.034	0.894 ± 0.080	0.860 ± 0.090
No clinical and no sequence features	0.752 ± 0.062	0.746 ± 0.063	0.788 ± 0.051	0.518 ± 0.126	0.812 ± 0.079	0.821 ± 0.070	0.879 ± 0.078	0.612 ± 0.111
No clinical and no structural features	0.782 ± 0.051	0.777 ± 0.052	0.808 ± 0.048	0.573 ± 0.106	0.838 ± 0.073	0.846 ± 0.048	0.882 ± 0.077	0.672 ± 0.080
No clinical features and no multi-task learning	0.778 ± 0.061	0.776 ± 0.060	0.795 ± 0.062	0.562 ± 0.123	0.859 ± 0.061	0.852 ± 0.056	0.831 ± 0.103	0.721 ± 0.079
No clinical and no 3D structural features	0.774 ± 0.072	0.772 ± 0.071	0.786 ± 0.074	0.551 ± 0.142	0.857 ± 0.062	0.855 ± 0.052	0.805 ± 0.110	0.739 ± 0.091

Supplementary Table 4. Full results of the internal *k*-fold cross-validation procedure. Values are shown as mean ± standard deviation. Abbreviations: MCC – Matthews Correlation Coefficient; AU-PRC – area under the precision-recall curve; AU-ROC – area under receiver operating characteristic curve.

Gene	Ref AA	Pos	Mut AA	Functional effect	GENTLY FULL CLASS	GENTLY FULL LOF	GENTLY FULL GOF	GENTLY NOPHENO CLASS	GENTLY NOPHENO LOF	GENTLY NOPHENO GOF	LOGOFUNC GOF	LOGOFUNC LOF	EVO_1B	EVO_40B
GABRA1	R	112	Q	LOF	LOF	0.916	0.084	LOF	0.664	0.336	0.231	0.563	0.001	-0.002
GABRA1	F	104	C	LOF	LOF	0.972	0.028	LOF	0.908	0.092	0.160	0.811	-0.001	-0.003
GABRA1	L	215	V	GOF	GOF	0.007	0.993	GOF	0.112	0.888	0.317	0.656	-0.001	-0.001
GABRA1	G	251	S	GOF	GOF	0.207	0.793	GOF	0.096	0.904	0.241	0.698	-0.002	-0.002
GABRA1	E	63	K	GOF	GOF	0.011	0.989	GOF	0.100	0.900	NA	NA	NA	NA
GABRA1	S	96	C	LOF	LOF	0.974	0.026	LOF	0.914	0.086	0.321	0.378	-0.001	-0.002
GABRA1	S	299	N	GOF	GOF	0.005	0.995	GOF	0.140	0.860	0.313	0.589	-0.001	-0.001
GABRG2	P	83	S	LOF	LOF	0.841	0.159	LOF	0.762	0.238	0.147	0.836	-0.003	-0.004
GABRA1	T	292	I	LOF	LOF	0.943	0.057	LOF	0.795	0.205	0.201	0.766	-0.003	-0.003
GABRB3	R	232	Q	LOF	LOF	0.965	0.035	LOF	0.786	0.214	0.377	0.500	-0.001	-0.002
GABRB3	Y	184	H	GOF	GOF	0.094	0.906	GOF	0.084	0.916	0.516	0.397	-0.002	-0.002
GABRB3	L	165	Q	GOF	GOF	0.028	0.972	GOF	0.133	0.867	NA	NA	NA	NA
GABRB3	Y	230	H	GOF	GOF	0.007	0.993	GOF	0.154	0.846	0.405	0.575	-0.001	-0.002
GABRB3	Q	249	K	GOF	GOF	0.030	0.970	GOF	0.446	0.554	0.608	0.354	-0.002	-0.003
GABRB2	Y	244	H	GOF	GOF	0.017	0.983	GOF	0.224	0.776	0.515	0.470	-0.003	-0.002
GABRB2	M	161	L	LOF	GOF	0.471	0.529	GOF	0.361	0.639	0.402	0.188	-0.001	-0.003
GABRB2	Y	181	F	LOF	LOF	0.991	0.009	LOF	0.833	0.167	0.363	0.206	-0.001	-0.002
GABRG2	Y	274	C	LOF	LOF	0.944	0.056	LOF	0.751	0.249	0.274	0.692	-0.003	-0.001
GABRB2	V	282	A	GOF	LOF	0.604	0.396	LOF	0.606	0.394	0.577	0.378	-0.002	-0.003
GABRB2	I	299	S	LOF	LOF	0.521	0.479	LOF	0.886	0.114	0.603	0.383	-0.002	-0.002
GABRA1	Y	252	C	GOF	GOF	0.013	0.987	GOF	0.337	0.663	0.254	0.693	-0.002	-0.002
GABRA1	V	287	I	GOF	GOF	0.230	0.770	GOF	0.371	0.629	NA	NA	NA	NA
GABRB3	V	41	M	LOF	LOF	0.957	0.043	LOF	0.913	0.087	0.223	0.266	-0.001	-0.002
GABRA1	V	287	G	LOF	LOF	0.684	0.316	LOF	0.888	0.112	0.094	0.752	-0.003	-0.003
GABRB3	L	52	V	LOF	LOF	0.850	0.150	LOF	0.916	0.084	0.188	0.347	-0.001	-0.003
GABRG2	S	139	N	GOF	GOF	0.090	0.910	LOF	0.691	0.309	0.131	0.358	-0.001	-0.001

GABRG2	N	457	S	GOF	GOF	0.043	0.957	LOF	0.824	0.176	0.415	0.463	-0.001	-0.010
GABRB2	L	247	R	GOF	GOF	0.011	0.989	GOF	0.180	0.820	0.445	0.540	-0.004	-0.005
GABRB3	M	162	I	LOF	LOF	0.918	0.082	LOF	0.682	0.318	0.424	0.292	-0.001	-0.002
GABRB2	I	299	V	LOF	LOF	0.809	0.191	LOF	0.725	0.275	0.376	0.132	-0.001	-0.001
GABRA1	A	308	V	LOF	LOF	0.913	0.087	LOF	0.807	0.193	0.231	0.731	-0.002	-0.002
GABRA1	A	188	D	LOF	LOF	0.870	0.130	LOF	0.583	0.417	0.193	0.763	-0.001	-0.002
GABRA1	H	83	N	GOF	GOF	0.034	0.966	GOF	0.126	0.874	0.185	0.308	-0.001	-0.003
GABRG2	R	82	W	LOF	LOF	0.894	0.106	LOF	0.861	0.139	0.201	0.788	-0.006	-0.009
GABRB2	H	291	P	GOF	GOF	0.193	0.807	LOF	0.717	0.283	0.715	0.246	-0.003	-0.004
GABRA1	T	294	K	GOF	GOF	0.112	0.888	LOF	0.624	0.376	0.169	0.804	-0.005	-0.006
GABRA1	A	332	V	LOF	LOF	0.982	0.018	LOF	0.856	0.144	0.194	0.761	-0.001	-0.002
GABRB3	E	180	G	GOF	GOF	0.016	0.984	GOF	0.177	0.823	0.445	0.508	0.000	-0.002
GABRA1	S	213	T	LOF	LOF	0.985	0.015	LOF	0.791	0.209	0.230	0.422	-0.001	-0.001
GABRA1	V	270	A	GOF	GOF	0.412	0.588	LOF	0.732	0.268	0.270	0.659	0.000	-0.002
GABRA1	N	275	K	LOF	LOF	0.987	0.013	LOF	0.655	0.345	0.466	0.433	-0.001	-0.001
GABRA1	I	317	L	LOF	GOF	0.236	0.764	LOF	0.756	0.244	0.349	0.503	-0.001	-0.002
GABRA1	A	332	T	LOF	LOF	0.917	0.083	LOF	0.575	0.425	0.191	0.779	-0.001	-0.002
GABRA1	P	181	S	LOF	LOF	0.871	0.129	LOF	0.873	0.127	0.156	0.431	-0.001	-0.002
GABRA1	A	322	D	LOF	LOF	0.915	0.085	LOF	0.769	0.231	0.274	0.702	-0.007	-0.004
GABRG2	R	177	G	LOF	GOF	0.437	0.563	GOF	0.223	0.777	0.194	0.644	-0.002	-0.005
GABRG2	N	79	S	GOF	GOF	0.050	0.950	LOF	0.544	0.456	0.182	0.457	-0.001	-0.002
GABRB3	R	142	L	GOF	LOF	0.629	0.371	GOF	0.356	0.644	0.382	0.572	-0.006	-0.004
GABRB3	L	284	P	LOF	LOF	0.926	0.074	GOF	0.493	0.507	0.267	0.716	-0.003	-0.003
GABRB3	T	288	N	GOF	GOF	0.022	0.978	GOF	0.236	0.764	0.501	0.432	-0.002	-0.002
GABRB3	A	305	V	LOF	LOF	0.905	0.095	GOF	0.399	0.601	0.428	0.555	-0.002	-0.001
GABRB3	S	433	L	LOF	GOF	0.270	0.730	GOF	0.474	0.526	0.327	0.125	-0.003	-0.002
GABRB3	M	311	T	GOF	GOF	0.158	0.842	GOF	0.384	0.616	0.511	0.391	-0.002	-0.001
GABRB2	I	299	L	LOF	LOF	0.944	0.056	LOF	0.920	0.080	0.684	0.212	-0.001	-0.001

GABRB2	T	184	I	GOF	GOF	0.005	0.995	GOF	0.257	0.743	0.398	0.517	-0.001	-0.004
GABRB2	F	224	C	LOF	LOF	0.752	0.248	LOF	0.830	0.170	0.421	0.493	-0.004	-0.005
GABRA1	L	215	P	GOF	GOF	0.010	0.990	GOF	0.283	0.717	0.197	0.793	-0.004	-0.004
GABRA1	R	94	H	GOF	GOF	0.004	0.996	GOF	0.082	0.918	0.249	0.712	-0.001	-0.002
GABRB3	Q	249	H	LOF	GOF	0.288	0.712	LOF	0.593	0.407	0.586	0.381	-0.002	-0.003
GABRB3	T	288	I	LOF	LOF	0.938	0.062	GOF	0.450	0.550	0.517	0.466	-0.002	-0.003
GABRA1	V	284	A	LOF	LOF	0.834	0.166	LOF	0.573	0.427	0.273	0.555	-0.001	-0.001
GABRA1	L	296	F	LOF	LOF	0.886	0.114	LOF	0.575	0.425	0.241	0.652	-0.002	-0.002
GABRG2	A	322	D	GOF	GOF	0.006	0.994	GOF	0.372	0.628	0.338	0.619	-0.003	-0.003
GABRB2	A	304	V	GOF	GOF	0.075	0.925	GOF	0.149	0.851	0.556	0.435	-0.001	-0.002
GABRB3	T	156	N	GOF	GOF	0.077	0.923	LOF	0.756	0.244	0.405	0.507	-0.001	-0.002
GABRA1	M	158	V	GOF	GOF	0.125	0.875	GOF	0.110	0.890	0.281	0.507	-0.001	-0.001
GABRB3	V	283	A	GOF	GOF	0.059	0.941	LOF	0.694	0.306	0.406	0.566	-0.001	-0.002
GABRB3	Y	184	C	LOF	GOF	0.274	0.726	LOF	0.746	0.254	0.471	0.463	-0.002	-0.002
GABRB2	T	249	R	GOF	GOF	0.002	0.998	GOF	0.198	0.802	0.479	0.508	-0.004	-0.004
GABRA1	M	263	I	GOF	LOF	0.656	0.344	GOF	0.151	0.849	0.373	0.597	-0.001	-0.001
GABRG2	T	90	M	LOF	LOF	0.928	0.072	LOF	0.817	0.183	0.208	0.765	-0.001	-0.002
GABRB3	F	318	S	LOF	LOF	0.910	0.090	LOF	0.765	0.235	0.481	0.497	-0.002	-0.002
GABRB3	I	257	M	LOF	GOF	0.020	0.980	GOF	0.284	0.716	0.559	0.411	-0.001	-0.001
GABRA1	S	76	R	LOF	LOF	0.983	0.017	LOF	0.849	0.151	0.298	0.669	-0.003	-0.005
GABRA1	L	146	M	LOF	LOF	0.992	0.008	GOF	0.444	0.556	0.250	0.385	-0.001	-0.001
GABRA1	R	214	H	LOF	LOF	0.901	0.099	LOF	0.615	0.385	0.287	0.652	-0.001	-0.001
GABRB3	Y	302	C	LOF	LOF	0.563	0.437	LOF	0.753	0.247	0.473	0.502	-0.004	-0.002
GABRA1	T	43	I	GOF	GOF	0.001	0.999	GOF	0.139	0.861	0.223	0.373	-0.002	-0.002
GABRA1	P	260	S	LOF	LOF	0.960	0.040	LOF	0.850	0.150	0.212	0.771	-0.003	-0.004
GABRA1	T	289	S	GOF	LOF	0.645	0.355	GOF	0.171	0.829	0.208	0.653	-0.003	-0.002
GABRG2	R	82	Q	GOF	GOF	0.473	0.527	LOF	0.625	0.375	0.158	0.725	-0.002	-0.003
GABRG2	P	282	S	GOF	GOF	0.246	0.754	LOF	0.826	0.174	0.188	0.755	-0.004	-0.003

GABRG2	V	459	F	GOF	GOF	0.007	0.993	LOF	0.618	0.382	0.373	0.420	-0.001	-0.009
GABRB3	T	157	M	GOF	LOF	0.960	0.040	LOF	0.708	0.292	0.449	0.470	-0.001	-0.002
GABRG2	P	83	T	GOF	GOF	0.006	0.994	GOF	0.282	0.718	0.160	0.823	-0.003	-0.006
GABRA1	V	162	M	GOF	LOF	0.923	0.077	GOF	0.461	0.539	0.145	0.594	-0.001	-0.001
GABRB3	R	217	C	GOF	LOF	0.885	0.115	GOF	0.349	0.651	0.499	0.236	-0.005	-0.002
GABRB2	P	252	L	GOF	GOF	0.001	0.999	GOF	0.101	0.899	0.389	0.596	-0.008	-0.005
GABRB2	I	288	S	LOF	LOF	0.721	0.279	LOF	0.578	0.422	0.590	0.388	-0.002	-0.004
GABRB2	I	288	T	LOF	LOF	0.988	0.012	LOF	0.600	0.400	0.603	0.340	-0.002	-0.002
GABRB2	V	302	M	GOF	GOF	0.108	0.892	GOF	0.383	0.617	0.636	0.318	-0.001	-0.001
GABRB2	T	249	A	GOF	GOF	0.030	0.970	GOF	0.405	0.595	0.545	0.388	-0.001	-0.001
GABRB2	N	289	H	LOF	LOF	0.999	0.001	LOF	0.889	0.111	0.739	0.164	-0.001	-0.004
GABRB2	M	79	T	LOF	LOF	0.975	0.025	LOF	0.891	0.109	NA	NA	NA	NA
GABRG2	I	344	L	LOF	LOF	0.893	0.107	LOF	0.573	0.427	0.236	0.240	0.000	-0.001
GABRG2	M	169	T	LOF	LOF	0.938	0.062	GOF	0.141	0.859	0.209	0.593	0.000	-0.002
GABRA1	T	295	A	LOF	LOF	0.994	0.006	LOF	0.890	0.110	0.235	0.709	-0.001	-0.002
GABRA1	Y	187	N	GOF	LOF	0.884	0.116	GOF	0.409	0.591	NA	NA	NA	NA
GABRB3	F	246	V	GOF	GOF	0.375	0.625	GOF	0.413	0.587	0.511	0.347	-0.002	-0.003
GABRB2	A	272	V	GOF	GOF	0.004	0.996	GOF	0.127	0.873	0.490	0.448	-0.002	-0.002
GABRB2	L	296	P	LOF	LOF	0.968	0.032	LOF	0.620	0.380	0.463	0.529	-0.002	-0.002
GABRB3	R	294	P	LOF	LOF	0.992	0.008	LOF	0.605	0.395	0.498	0.387	-0.002	-0.004
GABRB3	I	300	V	LOF	GOF	0.171	0.829	GOF	0.349	0.651	0.271	0.108	-0.001	-0.001
GABRA1	M	263	T	LOF	LOF	0.785	0.215	LOF	0.759	0.241	0.311	0.636	-0.001	-0.002
GABRA1	P	260	L	GOF	GOF	0.095	0.905	LOF	0.608	0.392	0.205	0.779	-0.006	-0.004
GABRA1	V	287	L	GOF	GOF	0.011	0.989	GOF	0.152	0.848	NA	NA	NA	NA
GABRA1	R	214	C	LOF	LOF	0.981	0.019	LOF	0.914	0.086	0.270	0.650	-0.002	-0.002
GABRB3	E	357	K	GOF	GOF	0.470	0.530	LOF	0.897	0.103	0.221	0.218	-0.002	-0.003
GABRA1	K	306	T	LOF	LOF	0.831	0.169	LOF	0.902	0.098	0.309	0.651	-0.002	-0.002
GABRA1	R	112	W	LOF	LOF	0.918	0.082	LOF	0.895	0.105	0.215	0.672	-0.001	-0.003

GABRB3	S	254	F	LOF	LOF	0.975	0.025	LOF	0.739	0.261	0.541	0.439	-0.002	-0.002
GABRA1	L	267	I	LOF	LOF	0.969	0.031	LOF	0.753	0.247	0.336	0.569	-0.001	-0.002
GABRA1	L	267	V	GOF	GOF	0.080	0.920	GOF	0.177	0.823	0.321	0.640	-0.001	-0.002
GABRG2	M	199	V	LOF	LOF	0.993	0.007	LOF	0.846	0.154	0.266	0.693	-0.001	-0.002
GABRG2	S	306	F	LOF	LOF	0.935	0.065	LOF	0.883	0.117	0.336	0.641	-0.001	-0.004
GABRG2	R	383	C	LOF	LOF	0.671	0.329	LOF	0.580	0.420	0.137	0.382	-0.003	-0.006
GABRB3	L	124	F	LOF	LOF	0.845	0.155	LOF	0.602	0.398	0.537	0.359	-0.001	-0.002
GABRB3	V	78	F	GOF	GOF	0.008	0.992	GOF	0.098	0.902	0.251	0.604	-0.001	-0.005
GABRB3	F	225	C	GOF	GOF	0.064	0.936	GOF	0.165	0.835	0.433	0.496	-0.002	-0.006
GABRB3	L	310	I	GOF	GOF	0.006	0.994	GOF	0.106	0.894	0.481	0.310	-0.001	-0.001
GABRB3	T	281	A	GOF	GOF	0.009	0.991	GOF	0.348	0.652	0.396	0.581	-0.001	-0.002
GABRB2	A	304	T	GOF	GOF	0.036	0.964	GOF	0.313	0.687	0.557	0.431	-0.001	-0.002
GABRB2	A	112	E	GOF	GOF	0.015	0.985	GOF	0.353	0.647	0.440	0.443	-0.002	-0.002
GABRB2	F	245	S	LOF	LOF	0.969	0.031	LOF	0.913	0.087	0.342	0.648	-0.003	-0.004
GABRB2	K	303	N	LOF	LOF	0.619	0.381	LOF	0.779	0.221	0.604	0.378	-0.002	-0.002
GABRB2	L	17	S	LOF	LOF	0.986	0.014	LOF	0.639	0.361	0.242	0.094	-0.006	-0.003
GABRA1	K	364	T	GOF	GOF	0.025	0.975	GOF	0.139	0.861	0.156	0.236	-0.004	-0.004
GABRB3	Q	210	E	GOF	GOF	0.140	0.860	GOF	0.162	0.838	0.655	0.273	-0.002	-0.002
GABRG2	I	85	K	GOF	GOF	0.001	0.999	GOF	0.088	0.912	0.197	0.694	-0.002	-0.007
GABRA1	N	216	I	LOF	LOF	0.650	0.350	LOF	0.691	0.309	0.444	0.492	-0.004	-0.005
GABRA1	K	306	N	GOF	GOF	0.144	0.856	LOF	0.705	0.295	0.343	0.522	-0.001	-0.003
GABRA1	A	281	V	LOF	LOF	0.893	0.107	LOF	0.807	0.193	0.253	0.717	-0.001	-0.002
GABRG2	V	104	G	GOF	GOF	0.101	0.899	LOF	0.646	0.354	0.140	0.774	-0.004	-0.004
GABRG2	P	282	A	GOF	GOF	0.045	0.955	GOF	0.227	0.773	0.193	0.754	-0.005	-0.004
GABRB2	L	283	I	LOF	LOF	0.939	0.061	LOF	0.889	0.111	0.551	0.246	-0.001	-0.003
GABRB2	Q	248	K	LOF	LOF	0.996	0.004	LOF	0.879	0.121	0.634	0.325	-0.002	-0.003
GABRG2	L	135	F	GOF	GOF	0.051	0.949	GOF	0.484	0.516	0.162	0.599	-0.001	-0.001
GABRA1	V	270	I	LOF	LOF	0.590	0.410	LOF	0.647	0.353	0.245	0.436	0.000	-0.001

GABRB3	R	142	H	LOF	LOF	0.974	0.026	LOF	0.711	0.289	0.376	0.548	-0.002	-0.002
GABRB2	R	240	T	GOF	GOF	0.031	0.969	LOF	0.646	0.354	0.346	0.635	-0.003	-0.005
GABRG2	A	106	T	LOF	LOF	0.687	0.313	LOF	0.792	0.208	0.112	0.459	-0.001	-0.001
GABRA1	V	74	I	LOF	LOF	0.721	0.279	LOF	0.602	0.398	0.201	0.380	0.000	-0.001
GABRA1	T	289	P	LOF	LOF	0.976	0.024	LOF	0.897	0.103	0.187	0.755	-0.005	-0.005
GABRB3	D	120	N	GOF	LOF	0.626	0.374	LOF	0.631	0.369	0.365	0.627	-0.002	-0.002
GABRA1	P	305	L	GOF	GOF	0.131	0.869	GOF	0.085	0.915	0.157	0.830	-0.004	-0.003
GABRA1	Y	189	F	LOF	LOF	0.878	0.122	LOF	0.639	0.361	0.176	0.599	-0.001	-0.003
GABRG2	I	107	T	LOF	GOF	0.263	0.737	GOF	0.300	0.700	0.131	0.534	-0.001	-0.003
GABRG2	R	323	W	GOF	LOF	0.549	0.451	GOF	0.293	0.707	0.308	0.658	-0.003	-0.002
GABRG2	P	302	L	LOF	LOF	0.845	0.155	LOF	0.612	0.388	0.268	0.712	-0.002	-0.003
GABRB3	P	301	L	GOF	GOF	0.054	0.946	LOF	0.625	0.375	0.567	0.412	-0.003	-0.002
GABRB3	E	77	K	GOF	GOF	0.136	0.864	GOF	0.083	0.917	0.186	0.676	-0.002	-0.004
GABRB3	N	110	D	LOF	LOF	0.932	0.068	LOF	0.811	0.189	0.361	0.458	-0.001	-0.001
GABRB3	L	284	M	GOF	GOF	0.071	0.929	GOF	0.117	0.883	0.359	0.255	-0.001	-0.002
GABRB3	Y	182	F	GOF	GOF	0.020	0.980	GOF	0.293	0.707	NA	NA	NA	NA
GABRA1	R	147	Q	GOF	GOF	0.032	0.968	GOF	0.150	0.850	0.268	0.710	-0.001	-0.002
GABRB3	R	232	P	GOF	GOF	0.050	0.950	GOF	0.147	0.853	0.351	0.626	-0.002	-0.004
GABRB3	A	305	T	LOF	LOF	0.740	0.260	LOF	0.518	0.482	0.432	0.546	-0.002	-0.001
GABRB2	A	159	S	GOF	GOF	0.475	0.525	GOF	0.330	0.670	0.352	0.152	-0.001	-0.002
GABRB2	Y	183	H	LOF	LOF	0.700	0.300	LOF	0.785	0.215	0.436	0.533	-0.001	-0.003
GABRB2	I	246	T	LOF	LOF	0.796	0.204	LOF	0.604	0.396	0.492	0.420	-0.003	-0.003
GABRB2	L	255	V	GOF	GOF	0.021	0.979	GOF	0.076	0.924	0.573	0.354	-0.001	-0.002
GABRA1	Y	252	H	LOF	LOF	0.827	0.173	GOF	0.328	0.672	0.254	0.718	-0.002	-0.002
GABRB2	F	331	S	GOF	GOF	0.054	0.946	LOF	0.691	0.309	0.653	0.224	-0.003	-0.002
GABRB2	Q	172	H	GOF	LOF	0.849	0.151	GOF	0.491	0.509	0.487	0.493	-0.001	-0.002
GABRB3	L	321	P	LOF	LOF	0.937	0.063	LOF	0.567	0.433	0.366	0.607	-0.008	-0.003
GABRB3	L	278	P	GOF	LOF	0.813	0.187	GOF	0.096	0.904	NA	NA	NA	NA

GABRB3	K	304	R	LOF	LOF	0.898	0.102	LOF	0.576	0.424	0.515	0.465	-0.001	-0.001
GABRA1	T	289	A	LOF	LOF	0.990	0.010	LOF	0.606	0.394	0.224	0.738	0.000	-0.001
GABRB3	Y	122	C	GOF	GOF	0.333	0.667	LOF	0.678	0.322	0.357	0.621	-0.004	-0.003
GABRB3	I	306	T	GOF	GOF	0.010	0.990	GOF	0.418	0.582	0.406	0.581	-0.002	-0.001
GABRB3	T	250	N	LOF	LOF	0.920	0.080	LOF	0.709	0.291	0.515	0.442	-0.002	-0.003
GABRA1	V	207	F	LOF	LOF	0.989	0.011	LOF	0.746	0.254	0.180	0.542	-0.003	-0.001
GABRG2	R	183	M	LOF	LOF	0.941	0.059	LOF	0.790	0.210	NA	NA	NA	NA
GABRB3	M	80	V	LOF	LOF	0.990	0.010	LOF	0.876	0.124	NA	NA	NA	NA

Supplementary Table 5. Predictions from *k*-fold cross-validation across the 174 unique variants in our training dataset. Predictions are shown for the full model (GENTLY_FULL) and the model without clinical features (GENTLY_NOPHENO) separately and shown side-by-side to the experimentally observed functional effect. Abbreviations: GOF – gain-of-function; LOF – loss-of-function; NA – not available.

Model	Accuracy	Balanced Accuracy	F1 Score	MCC	AU-PRC	AU-ROC	Sensitivity	Specificity	DeLong test vs. full model	DeLong test vs. model without clinical features
GENTLY: Full model	**0.880 ± 0.024**	**0.878 ± 0.025**	**0.886 ± 0.025**	**0.758 ± 0.049**	**0.946 ± 0.019**	**0.946 ± 0.016**	**0.901 ± 0.031**	0.856 ± 0.039	–	–
GENTLY: Model without clinical features	0.804 ± 0.030	0.801 ± 0.030	0.822 ± 0.030	0.610 ± 0.060	0.863 ± 0.035	0.862 ± 0.028	0.868 ± 0.036	0.735 ± 0.048	p = 0.083	–
Clinical decision-making	0.810 ± 0.030	0.807 ± 0.030	0.828 ± 0.030	0.623 ± 0.059	0.863 ± 0.035	0.864 ± 0.028	0.879 ± 0.034	0.735 ± 0.049	p = 0.040	p = 0.588
evo2 (zero-shot)	0.543 ± 0.039	0.507 ± 0.007	0.700 ± 0.033	0.103 ± 0.026	0.601 ± 0.057	0.560 ± 0.045	1.000 ± 0	0.013 ± 0.013	p < 0.001	P = 0.002
evo2 (supervised)	0.619 ± 0.070	0.666 ± 0.057	0.571 ± 0.090	0.354 ± 0.116	0.841 ± 0.055	0.715 ± 0.074	0.431 ± 0.092	**0.901 ± 0.068**	p = 0.038	p = 0.208
LoGoFunc	0.488 ± 0.039	0.484 ± 0.039	0.527 ± 0.046	-0.032 ± 0.077	0.547 ± 0.055	0.495 ± 0.045	0.534 ± 0.053	0.434 ± 0.056	p < 0.001	p < 0.001

Supplementary Table 6. Baseline comparisons. Pre-trained models (evo2 zero-shot, LoGoFunc) and models trained and evaluated on genome-level variants (evo2 supervised) were not matched to the same cross-validation folds as GENTLY and the clinical decision-making baseline. Therefore, comparisons were made by sampling across 10,000 bootstrap iterations, reporting mean and standard deviation, to estimate the variance and bias for each performance metric. Therefore, values may deviate from those reported in the main text. For each performance metric, the highest mean value across models is shown in bold. Model performance was compared using DeLong's test, and the median p-value across bootstrap iterations is reported.

Group	Gene	Ref AA	Pos	Mut AA	Functional Effect	GENTLY FULL CLASS	GENTLY FULL GOF	GENTLY FULL LOF	GENTLY NOPHENO CLASS	GENTLY NOPHENO LOF	GENTLY NOPHENO GOF	LOGOFUNC NEUTRAL	LOGOFUNC GOF	LOGOFUNC LOF	EVO
In distribution	***GABRA1***	M	263	K	GOF	GOF	0.845	0.155	GOF	0.108	0.892	0.025	0.253	0.721	-0.004
In distribution	***GABRA1***	R	301	V	LOF	LOF	0.081	0.919	LOF	0.741	0.259	NA	NA	NA	NA
Zero-shot	***GABRA3***	L	179	V	GOF	GOF	0.974	0.026	LOF	0.874	0.126	0.428	0.042	0.530	-0.001
Zero-shot	***GABRA3***	L	316	F	GOF	GOF	0.962	0.038	GOF	0.279	0.721	0.082	0.032	0.886	-0.001
Zero-shot	***GABRA3***	Q	242	L	GOF	LOF	0.378	0.622	LOF	0.742	0.258	0.047	0.040	0.914	-0.007
Zero-shot	***GABRA3***	T	314	P	GOF	GOF	0.960	0.040	GOF	0.106	0.894	0.144	0.030	0.825	-0.002
Zero-shot	***GABRA3***	V	309	A	GOF	GOF	0.982	0.018	GOF	0.171	0.829	0.376	0.035	0.589	0.000
Zero-shot	***GABRA3***	V	309	L	GOF	LOF	0.455	0.545	GOF	0.249	0.751	0.344	0.034	0.622	0.000
Zero-shot	***GABRA3***	Y	277	C	GOF	GOF	0.600	0.400	GOF	0.341	0.659	0.068	0.046	0.886	-0.002
Zero-shot	***GABRA3***	Y	346	C	GOF	GOF	0.821	0.179	LOF	0.762	0.238	0.246	0.056	0.698	-0.002
Zero-shot	***GABRA3***	Y	474	C	GOF	GOF	0.877	0.123	GOF	0.133	0.867	0.066	0.050	0.884	-0.006
Zero-shot	***GABRA5***	A	339	V	GOF	GOF	0.890	0.110	GOF	0.133	0.867	0.063	0.193	0.744	-0.002
Zero-shot	***GABRA5***	R	166	G	GOF	LOF	0.242	0.758	LOF	0.690	0.310	NA	NA	NA	NA
Zero-shot	***GABRA5***	T	301	R	GOF	GOF	0.989	0.011	GOF	0.387	0.613	0.040	0.192	0.768	-0.004
Zero-shot	***GABRA5***	V	291	L	GOF	LOF	0.215	0.785	GOF	0.263	0.737	0.218	0.234	0.548	-0.001
Zero-shot	***GABRA5***	V	294	F	GOF	GOF	0.980	0.020	GOF	0.424	0.576	NA	NA	NA	NA
Zero-shot	***GABRB1***	F	246	S	GOF	GOF	0.719	0.281	GOF	0.184	0.816	0.034	0.120	0.846	-0.004
Zero-shot	***GABRB1***	I	247	T	GOF	GOF	0.948	0.052	GOF	0.138	0.862	0.425	0.090	0.485	-0.002
Zero-shot	***GABRB1***	T	281	S	GOF	GOF	0.990	0.010	GOF	0.283	0.717	0.229	0.125	0.646	-0.001
Zero-shot	***GABRB1***	T	285	K	GOF	GOF	0.998	0.002	GOF	0.211	0.789	0.081	0.146	0.772	-0.006
Zero-shot	***GABRB1***	V	63	A	LOF	LOF	0.249	0.751	LOF	0.548	0.452	0.142	0.039	0.820	-0.003
In distribution	***GABRB3***	R	294	P	GOF	LOF	0.445	0.555	GOF	0.248	0.752	0.115	0.498	0.387	-0.002
In distribution	***GABRB3***	T	285	A	GOF	LOF	0.186	0.814	GOF	0.067	0.933	0.410	0.326	0.264	-0.001
Zero-shot	***GABRD***	I	284	T	GOF	LOF	0.064	0.936	LOF	0.722	0.278	0.057	0.536	0.406	-0.001
Zero-shot	***GABRD***	L	260	V	GOF	GOF	0.622	0.378	GOF	0.148	0.852	0.296	0.386	0.318	-0.001
Zero-shot	***GABRD***	P	257	L	GOF	LOF	0.184	0.816	GOF	0.197	0.803	0.056	0.362	0.582	-0.005

Zero-shot	***GABRD***	T	2911	GOF	LOF	0.034	0.966	LOF	0.833	0.167	0.016	0.528	0.457	-0.002

Supplementary Table 7. Predictions on the external validation dataset. Predictions are shown for the full model (GENTLY_FULL) and the model without clinical features (GENTLY_NOPHENO) separately and shown side-by-side to the experimentally observed functional effect. Abbreviations: GOF – gain-of-function; LOF – loss-of-function; NA – not available. Note that misclassifications can occur even if area under the ROC curve (AU-ROC) is high, which motivates our population-based identification of score thresholds.

Percentile	TP	FP	TN	FN	pLOF lower bound	pLOF upper bound	pGOF lower bound	pGOF upper bound	Sensitivity	Specificity	Precision	FDR	F1 score	Accuracy	Subset
**1**	9	12	1866	165	0.928	0.972	0.028	0.072	0.052	0.994	0.429	0.571	0.092	0.914	GOF
**2**	49	8	1870	125	0.920	0.928	0.072	0.080	0.282	0.996	0.860	0.140	0.424	0.935	GOF
**3**	3	10	1868	171	0.907	0.920	0.080	0.093	0.017	0.995	0.231	0.769	0.032	0.912	GOF
**4**	1	13	1865	173	0.896	0.907	0.093	0.104	0.006	0.993	0.071	0.929	0.011	0.909	GOF
**5**	0	16	1862	174	0.888	0.896	0.104	0.112	0.000	0.991	0.000	1.000	NA	0.907	GOF
**6**	0	23	1855	174	0.882	0.888	0.112	0.118	0.000	0.988	0.000	1.000	NA	0.904	GOF
**7**	1	17	1861	173	0.876	0.882	0.118	0.124	0.006	0.991	0.056	0.944	0.010	0.907	GOF
**8**	1	18	1860	173	0.870	0.876	0.124	0.130	0.006	0.990	0.053	0.947	0.010	0.907	GOF
**9**	0	21	1857	174	0.865	0.870	0.130	0.135	0.000	0.989	0.000	1.000	NA	0.905	GOF
**10**	0	12	1866	174	0.860	0.865	0.135	0.140	0.000	0.994	0.000	1.000	NA	0.909	GOF
**11**	0	21	1857	174	0.855	0.860	0.140	0.145	0.000	0.989	0.000	1.000	NA	0.905	GOF
**12**	3	22	1856	171	0.849	0.855	0.145	0.151	0.017	0.988	0.120	0.880	0.030	0.906	GOF
**13**	1	16	1862	173	0.844	0.849	0.151	0.156	0.006	0.991	0.059	0.941	0.010	0.908	GOF
**14**	0	16	1862	174	0.841	0.844	0.156	0.159	0.000	0.991	0.000	1.000	NA	0.907	GOF
**15**	1	18	1860	173	0.838	0.841	0.159	0.162	0.006	0.990	0.053	0.947	0.010	0.907	GOF
**16**	0	18	1860	174	0.833	0.838	0.162	0.167	0.000	0.990	0.000	1.000	NA	0.906	GOF
**17**	1	25	1853	173	0.830	0.833	0.167	0.170	0.006	0.987	0.038	0.962	0.010	0.904	GOF
**18**	0	19	1859	174	0.827	0.830	0.170	0.173	0.000	0.990	0.000	1.000	NA	0.906	GOF
**19**	0	12	1866	174	0.824	0.827	0.173	0.176	0.000	0.994	0.000	1.000	NA	0.909	GOF
**20**	1	18	1860	173	0.820	0.824	0.176	0.180	0.006	0.990	0.053	0.947	0.010	0.907	GOF
**21**	0	12	1866	174	0.817	0.820	0.180	0.183	0.000	0.994	0.000	1.000	NA	0.909	GOF
**22**	2	22	1856	172	0.813	0.817	0.183	0.187	0.011	0.988	0.083	0.917	0.020	0.905	GOF
**23**	1	18	1860	173	0.809	0.813	0.187	0.191	0.006	0.990	0.053	0.947	0.010	0.907	GOF
**24**	1	17	1861	173	0.806	0.809	0.191	0.194	0.006	0.991	0.056	0.944	0.010	0.907	GOF
**25**	1	26	1852	173	0.804	0.806	0.194	0.196	0.006	0.986	0.037	0.963	0.010	0.903	GOF
**26**	0	22	1856	174	0.801	0.804	0.196	0.199	0.000	0.988	0.000	1.000	NA	0.904	GOF
**27**	0	17	1861	174	0.798	0.801	0.199	0.202	0.000	0.991	0.000	1.000	NA	0.907	GOF

**28**	0	22	1856	174	0.795	0.798	0.202	0.205	0.000	0.988	0.000	1.000	NA	0.904	GOF
**29**	1	23	1855	173	0.794	0.795	0.205	0.206	0.006	0.988	0.042	0.958	0.010	0.904	GOF
**30**	0	21	1857	174	0.792	0.794	0.206	0.208	0.000	0.989	0.000	1.000	NA	0.905	GOF
**31**	0	20	1858	174	0.790	0.792	0.208	0.210	0.000	0.989	0.000	1.000	NA	0.905	GOF
**32**	0	21	1857	174	0.790	0.790	0.210	0.210	0.000	0.989	0.000	1.000	NA	0.905	GOF
**33**	0	23	1855	174	0.790	0.790	0.210	0.210	0.000	0.988	0.000	1.000	NA	0.904	GOF
**34**	0	13	1865	174	0.789	0.790	0.210	0.211	0.000	0.993	0.000	1.000	NA	0.909	GOF
**35**	1	17	1861	173	0.788	0.789	0.211	0.212	0.006	0.991	0.056	0.944	0.010	0.907	GOF
**36**	0	16	1862	174	0.788	0.788	0.212	0.212	0.000	0.991	0.000	1.000	NA	0.907	GOF
**37**	0	24	1854	174	0.787	0.788	0.212	0.213	0.000	0.987	0.000	1.000	NA	0.904	GOF
**38**	0	25	1853	174	0.786	0.787	0.213	0.214	0.000	0.987	0.000	1.000	NA	0.903	GOF
**39**	0	28	1850	174	0.786	0.786	0.214	0.214	0.000	0.985	0.000	1.000	NA	0.902	GOF
**40**	0	18	1860	174	0.784	0.786	0.214	0.216	0.000	0.990	0.000	1.000	NA	0.906	GOF
**41**	0	28	1850	174	0.783	0.784	0.216	0.217	0.000	0.985	0.000	1.000	NA	0.902	GOF
**42**	0	17	1861	174	0.782	0.783	0.217	0.218	0.000	0.991	0.000	1.000	NA	0.907	GOF
**43**	0	25	1853	174	0.782	0.782	0.218	0.218	0.000	0.987	0.000	1.000	NA	0.903	GOF
**44**	0	15	1863	174	0.781	0.781	0.219	0.219	0.000	0.992	0.000	1.000	NA	0.908	GOF
**45**	1	17	1861	173	0.780	0.781	0.219	0.220	0.006	0.991	0.056	0.944	0.010	0.907	GOF
**46**	0	34	1844	174	0.780	0.780	0.220	0.220	0.000	0.982	0.000	1.000	NA	0.899	GOF
**47**	0	25	1853	174	0.778	0.780	0.220	0.222	0.000	0.987	0.000	1.000	NA	0.903	GOF
**48**	0	28	1850	174	0.778	0.778	0.222	0.222	0.000	0.985	0.000	1.000	NA	0.902	GOF
**49**	0	28	1850	174	0.777	0.778	0.222	0.223	0.000	0.985	0.000	1.000	NA	0.902	GOF
**50**	0	29	1849	174	0.776	0.777	0.223	0.224	0.000	0.985	0.000	1.000	NA	0.901	GOF
**51**	0	16	1862	174	0.776	0.776	0.224	0.224	0.000	0.991	0.000	1.000	NA	0.907	GOF
**52**	0	21	1857	174	0.773	0.776	0.224	0.227	0.000	0.989	0.000	1.000	NA	0.905	GOF
**53**	0	23	1855	174	0.773	0.773	0.227	0.227	0.000	0.988	0.000	1.000	NA	0.904	GOF
**54**	0	27	1851	174	0.772	0.773	0.227	0.228	0.000	0.986	0.000	1.000	NA	0.902	GOF
**55**	1	22	1856	173	0.769	0.772	0.228	0.231	0.006	0.988	0.043	0.957	0.010	0.905	GOF

**56**	0	16	1862	174	0.767	0.769	0.231	0.233	0.000	0.991	0.000	1.000	NA	0.907	GOF
**57**	0	24	1854	174	0.764	0.767	0.233	0.236	0.000	0.987	0.000	1.000	NA	0.904	GOF
**58**	0	26	1852	174	0.763	0.764	0.236	0.237	0.000	0.986	0.000	1.000	NA	0.903	GOF
**59**	0	22	1856	174	0.761	0.763	0.237	0.239	0.000	0.988	0.000	1.000	NA	0.904	GOF
**60**	0	19	1859	174	0.759	0.761	0.239	0.241	0.000	0.990	0.000	1.000	NA	0.906	GOF
**61**	0	27	1851	174	0.759	0.759	0.241	0.241	0.000	0.986	0.000	1.000	NA	0.902	GOF
**62**	1	22	1856	173	0.755	0.759	0.241	0.245	0.006	0.988	0.043	0.957	0.010	0.905	GOF
**63**	0	16	1862	174	0.752	0.755	0.245	0.248	0.000	0.991	0.000	1.000	NA	0.907	GOF
**64**	2	19	1859	172	0.745	0.751	0.249	0.255	0.011	0.990	0.095	0.905	0.021	0.907	GOF
**65**	0	16	1862	174	0.740	0.745	0.255	0.260	0.000	0.991	0.000	1.000	NA	0.907	GOF
**66**	0	16	1862	174	0.737	0.740	0.260	0.263	0.000	0.991	0.000	1.000	NA	0.907	GOF
**67**	0	14	1864	174	0.733	0.737	0.263	0.267	0.000	0.993	0.000	1.000	NA	0.908	GOF
**68**	2	18	1860	172	0.729	0.733	0.267	0.271	0.011	0.990	0.100	0.900	0.021	0.907	GOF
**69**	0	16	1862	174	0.722	0.729	0.271	0.278	0.000	0.991	0.000	1.000	NA	0.907	GOF
**70**	1	11	1867	173	0.717	0.722	0.278	0.283	0.006	0.994	0.083	0.917	0.011	0.910	GOF
**71**	0	17	1861	174	0.709	0.717	0.283	0.291	0.000	0.991	0.000	1.000	NA	0.907	GOF
**72**	1	13	1865	173	0.703	0.709	0.291	0.297	0.006	0.993	0.071	0.929	0.011	0.909	GOF
**73**	1	18	1860	173	0.695	0.703	0.297	0.305	0.006	0.990	0.053	0.947	0.010	0.907	GOF
**74**	0	13	1865	174	0.688	0.695	0.305	0.312	0.000	0.993	0.000	1.000	NA	0.909	GOF
**75**	0	23	1855	174	0.682	0.688	0.312	0.318	0.000	0.988	0.000	1.000	NA	0.904	GOF
**76**	0	21	1857	174	0.679	0.682	0.318	0.321	0.000	0.989	0.000	1.000	NA	0.905	GOF
**77**	0	26	1852	174	0.679	0.679	0.321	0.321	0.000	0.986	0.000	1.000	NA	0.903	GOF
**78**	0	27	1851	174	0.671	0.678	0.322	0.329	0.000	0.986	0.000	1.000	NA	0.902	GOF
**79**	0	14	1864	174	0.662	0.671	0.329	0.338	0.000	0.993	0.000	1.000	NA	0.908	GOF
**80**	1	20	1858	173	0.651	0.662	0.338	0.349	0.006	0.989	0.048	0.952	0.010	0.906	GOF
**81**	0	12	1866	174	0.643	0.651	0.349	0.357	0.000	0.994	0.000	1.000	NA	0.909	GOF
**82**	2	16	1862	172	0.640	0.643	0.357	0.360	0.011	0.991	0.111	0.889	0.021	0.908	GOF
**83**	0	18	1860	174	0.638	0.640	0.360	0.362	0.000	0.990	0.000	1.000	NA	0.906	GOF

**84**	1	21	1857	173	0.628	0.638	0.362	0.372	0.006	0.989	0.045	0.955	0.010	0.905	GOF
**85**	0	12	1866	174	0.617	0.628	0.372	0.383	0.000	0.994	0.000	1.000	NA	0.909	GOF
**86**	1	13	1865	173	0.604	0.616	0.384	0.396	0.006	0.993	0.071	0.929	0.011	0.909	GOF
**87**	0	19	1859	174	0.592	0.603	0.397	0.408	0.000	0.990	0.000	1.000	NA	0.906	GOF
**88**	0	17	1861	174	0.588	0.592	0.408	0.412	0.000	0.991	0.000	1.000	NA	0.907	GOF
**89**	1	17	1861	173	0.588	0.588	0.412	0.412	0.006	0.991	0.056	0.944	0.010	0.907	GOF
**90**	1	20	1858	173	0.579	0.588	0.412	0.421	0.006	0.989	0.048	0.952	0.010	0.906	GOF
**91**	0	18	1860	174	0.561	0.579	0.421	0.439	0.000	0.990	0.000	1.000	NA	0.906	GOF
**92**	0	19	1859	174	0.538	0.560	0.440	0.462	0.000	0.990	0.000	1.000	NA	0.906	GOF
**93**	1	23	1855	173	0.518	0.538	0.462	0.482	0.006	0.988	0.042	0.958	0.010	0.904	GOF
**94**	1	16	1862	173	0.491	0.517	0.483	0.509	0.006	0.991	0.059	0.941	0.010	0.908	GOF
**95**	0	16	1862	174	0.456	0.490	0.510	0.544	0.000	0.991	0.000	1.000	NA	0.907	GOF
**96**	3	16	1862	171	0.409	0.456	0.544	0.591	0.017	0.991	0.158	0.842	0.031	0.909	GOF
**97**	2	14	1864	172	0.346	0.409	0.591	0.654	0.011	0.993	0.125	0.875	0.021	0.909	GOF
**98**	4	10	1868	170	0.271	0.345	0.655	0.729	0.023	0.995	0.286	0.714	0.043	0.912	GOF
**99**	10	9	1869	164	0.150	0.271	0.729	0.850	0.057	0.995	0.526	0.474	0.104	0.916	GOF
**100**	58	3	1875	116	0.032	0.150	0.850	0.968	0.333	0.998	0.951	0.049	0.494	0.942	GOF
**1**	59	3	1875	115	0.032	0.150	0.850	0.968	0.339	0.998	0.952	0.048	0.500	0.942	LOF
**2**	10	9	1869	164	0.152	0.271	0.729	0.848	0.057	0.995	0.526	0.474	0.104	0.916	LOF
**3**	3	10	1868	171	0.271	0.347	0.653	0.729	0.017	0.995	0.231	0.769	0.032	0.912	LOF
**4**	2	15	1863	172	0.347	0.411	0.589	0.653	0.011	0.992	0.118	0.882	0.021	0.909	LOF
**5**	3	15	1863	171	0.412	0.458	0.542	0.588	0.017	0.992	0.167	0.833	0.031	0.909	LOF
**6**	0	18	1860	174	0.459	0.494	0.506	0.541	0.000	0.990	0.000	1.000	NA	0.906	LOF
**7**	1	15	1863	173	0.495	0.519	0.481	0.505	0.006	0.992	0.063	0.938	0.011	0.908	LOF
**8**	1	23	1855	173	0.519	0.540	0.460	0.481	0.006	0.988	0.042	0.958	0.010	0.904	LOF
**9**	0	19	1859	174	0.540	0.563	0.437	0.460	0.000	0.990	0.000	1.000	NA	0.906	LOF
**10**	0	18	1860	174	0.563	0.581	0.419	0.437	0.000	0.990	0.000	1.000	NA	0.906	LOF
**11**	1	21	1857	173	0.581	0.588	0.412	0.419	0.006	0.989	0.045	0.955	0.010	0.905	LOF

**12**	1	16	1862	173	0.588	0.588	0.412	0.412	0.006	0.991	0.059	0.941	0.010	0.908	LOF
**13**	0	21	1857	174	0.588	0.593	0.407	0.412	0.000	0.989	0.000	1.000	NA	0.905	LOF
**14**	0	19	1859	174	0.594	0.605	0.395	0.406	0.000	0.990	0.000	1.000	NA	0.906	LOF
**15**	1	11	1867	173	0.605	0.619	0.381	0.395	0.006	0.994	0.083	0.917	0.011	0.910	LOF
**16**	1	13	1865	173	0.619	0.630	0.370	0.381	0.006	0.993	0.071	0.929	0.011	0.909	LOF
**17**	0	19	1859	174	0.630	0.639	0.361	0.370	0.000	0.990	0.000	1.000	NA	0.906	LOF
**18**	1	18	1860	173	0.639	0.640	0.360	0.361	0.006	0.990	0.053	0.947	0.010	0.907	LOF
**19**	1	15	1863	173	0.640	0.644	0.356	0.360	0.006	0.992	0.063	0.938	0.011	0.908	LOF
**20**	0	14	1864	174	0.644	0.653	0.347	0.356	0.000	0.993	0.000	1.000	NA	0.908	LOF
**21**	1	19	1859	173	0.653	0.664	0.336	0.347	0.006	0.990	0.050	0.950	0.010	0.906	LOF
**22**	0	20	1858	174	0.664	0.673	0.327	0.336	0.000	0.989	0.000	1.000	NA	0.905	LOF
**23**	0	29	1849	174	0.674	0.679	0.321	0.326	0.000	0.985	0.000	1.000	NA	0.901	LOF
**24**	0	22	1856	174	0.679	0.679	0.321	0.321	0.000	0.988	0.000	1.000	NA	0.904	LOF
**25**	0	26	1852	174	0.679	0.683	0.317	0.321	0.000	0.986	0.000	1.000	NA	0.903	LOF
**26**	0	15	1863	174	0.683	0.690	0.310	0.317	0.000	0.992	0.000	1.000	NA	0.908	LOF
**27**	1	16	1862	173	0.690	0.698	0.302	0.310	0.006	0.991	0.059	0.941	0.010	0.908	LOF
**28**	0	14	1864	174	0.698	0.705	0.295	0.302	0.000	0.993	0.000	1.000	NA	0.908	LOF
**29**	1	13	1865	173	0.705	0.711	0.289	0.295	0.006	0.993	0.071	0.929	0.011	0.909	LOF
**30**	0	17	1861	174	0.712	0.718	0.282	0.288	0.000	0.991	0.000	1.000	NA	0.907	LOF
**31**	1	13	1865	173	0.718	0.724	0.276	0.282	0.006	0.993	0.071	0.929	0.011	0.909	LOF
**32**	0	18	1860	174	0.724	0.731	0.269	0.276	0.000	0.990	0.000	1.000	NA	0.906	LOF
**33**	2	19	1859	172	0.731	0.736	0.264	0.269	0.011	0.990	0.095	0.905	0.021	0.907	LOF
**34**	0	16	1862	174	0.736	0.738	0.262	0.264	0.000	0.991	0.000	1.000	NA	0.907	LOF
**35**	0	15	1863	174	0.738	0.742	0.258	0.262	0.000	0.992	0.000	1.000	NA	0.908	LOF
**36**	0	22	1856	174	0.742	0.747	0.253	0.258	0.000	0.988	0.000	1.000	NA	0.904	LOF
**37**	2	15	1863	172	0.747	0.753	0.247	0.253	0.011	0.992	0.118	0.882	0.021	0.909	LOF
**38**	0	13	1865	174	0.753	0.757	0.243	0.247	0.000	0.993	0.000	1.000	NA	0.909	LOF
**39**	1	31	1847	173	0.757	0.759	0.241	0.243	0.006	0.983	0.031	0.969	0.010	0.901	LOF

**40**	0	18	1860	174	0.759	0.760	0.240	0.241	0.000	0.990	0.000	1.000	NA	0.906	LOF
**41**	0	24	1854	174	0.760	0.763	0.237	0.240	0.000	0.987	0.000	1.000	NA	0.904	LOF
**42**	0	23	1855	174	0.763	0.763	0.237	0.237	0.000	0.988	0.000	1.000	NA	0.904	LOF
**43**	0	23	1855	174	0.763	0.765	0.235	0.237	0.000	0.988	0.000	1.000	NA	0.904	LOF
**44**	0	23	1855	174	0.765	0.767	0.233	0.235	0.000	0.988	0.000	1.000	NA	0.904	LOF
**45**	1	16	1862	173	0.767	0.771	0.229	0.233	0.006	0.991	0.059	0.941	0.010	0.908	LOF
**46**	0	25	1853	174	0.771	0.773	0.227	0.229	0.000	0.987	0.000	1.000	NA	0.903	LOF
**47**	0	27	1851	174	0.773	0.773	0.227	0.227	0.000	0.986	0.000	1.000	NA	0.902	LOF
**48**	0	19	1859	174	0.773	0.775	0.225	0.227	0.000	0.990	0.000	1.000	NA	0.906	LOF
**49**	0	23	1855	174	0.775	0.776	0.224	0.225	0.000	0.988	0.000	1.000	NA	0.904	LOF
**50**	0	18	1860	174	0.776	0.776	0.224	0.224	0.000	0.990	0.000	1.000	NA	0.906	LOF
**51**	0	30	1848	174	0.776	0.778	0.222	0.224	0.000	0.984	0.000	1.000	NA	0.901	LOF
**52**	0	30	1848	174	0.778	0.778	0.222	0.222	0.000	0.984	0.000	1.000	NA	0.901	LOF
**53**	0	25	1853	174	0.778	0.779	0.221	0.222	0.000	0.987	0.000	1.000	NA	0.903	LOF
**54**	0	28	1850	174	0.779	0.780	0.220	0.221	0.000	0.985	0.000	1.000	NA	0.902	LOF
**55**	0	30	1848	174	0.780	0.781	0.219	0.220	0.000	0.984	0.000	1.000	NA	0.901	LOF
**56**	1	18	1860	173	0.781	0.781	0.219	0.219	0.006	0.990	0.053	0.947	0.010	0.907	LOF
**57**	0	15	1863	174	0.781	0.782	0.218	0.219	0.000	0.992	0.000	1.000	NA	0.908	LOF
**58**	0	24	1854	174	0.782	0.782	0.218	0.218	0.000	0.987	0.000	1.000	NA	0.904	LOF
**59**	0	21	1857	174	0.782	0.783	0.217	0.218	0.000	0.989	0.000	1.000	NA	0.905	LOF
**60**	0	19	1859	174	0.783	0.785	0.215	0.217	0.000	0.990	0.000	1.000	NA	0.906	LOF
**61**	0	27	1851	174	0.785	0.786	0.214	0.215	0.000	0.986	0.000	1.000	NA	0.902	LOF
**62**	0	27	1851	174	0.786	0.786	0.214	0.214	0.000	0.986	0.000	1.000	NA	0.902	LOF
**63**	0	22	1856	174	0.786	0.787	0.213	0.214	0.000	0.988	0.000	1.000	NA	0.904	LOF
**64**	0	21	1857	174	0.787	0.788	0.212	0.213	0.000	0.989	0.000	1.000	NA	0.905	LOF
**65**	1	12	1866	173	0.788	0.788	0.212	0.212	0.006	0.994	0.077	0.923	0.011	0.910	LOF
**66**	0	17	1861	174	0.788	0.789	0.211	0.212	0.000	0.991	0.000	1.000	NA	0.907	LOF
**67**	0	16	1862	174	0.789	0.790	0.210	0.211	0.000	0.991	0.000	1.000	NA	0.907	LOF

**68**	0	27	1851	174	0.790	0.790	0.210	0.210	0.000	0.986	0.000	1.000	NA	0.902	LOF
**69**	0	16	1862	174	0.790	0.791	0.209	0.210	0.000	0.991	0.000	1.000	NA	0.907	LOF
**70**	0	24	1854	174	0.791	0.792	0.208	0.209	0.000	0.987	0.000	1.000	NA	0.904	LOF
**71**	0	18	1860	174	0.792	0.794	0.206	0.208	0.000	0.990	0.000	1.000	NA	0.906	LOF
**72**	1	26	1852	173	0.794	0.796	0.204	0.206	0.006	0.986	0.037	0.963	0.010	0.903	LOF
**73**	0	18	1860	174	0.796	0.799	0.201	0.204	0.000	0.990	0.000	1.000	NA	0.906	LOF
**74**	0	14	1864	174	0.799	0.801	0.199	0.201	0.000	0.993	0.000	1.000	NA	0.908	LOF
**75**	0	28	1850	174	0.802	0.804	0.196	0.198	0.000	0.985	0.000	1.000	NA	0.902	LOF
**76**	1	21	1857	173	0.804	0.807	0.193	0.196	0.006	0.989	0.045	0.955	0.010	0.905	LOF
**77**	1	19	1859	173	0.807	0.810	0.190	0.193	0.006	0.990	0.050	0.950	0.010	0.906	LOF
**78**	2	19	1859	172	0.810	0.814	0.186	0.190	0.011	0.990	0.095	0.905	0.021	0.907	LOF
**79**	1	17	1861	173	0.814	0.817	0.183	0.186	0.006	0.991	0.056	0.944	0.010	0.907	LOF
**80**	0	14	1864	174	0.817	0.821	0.179	0.183	0.000	0.993	0.000	1.000	NA	0.908	LOF
**81**	1	15	1863	173	0.821	0.824	0.176	0.179	0.006	0.992	0.063	0.938	0.011	0.908	LOF
**82**	0	14	1864	174	0.824	0.828	0.172	0.176	0.000	0.993	0.000	1.000	NA	0.908	LOF
**83**	0	21	1857	174	0.828	0.831	0.169	0.172	0.000	0.989	0.000	1.000	NA	0.905	LOF
**84**	1	24	1854	173	0.831	0.834	0.166	0.169	0.006	0.987	0.040	0.960	0.010	0.904	LOF
**85**	0	18	1860	174	0.834	0.839	0.161	0.166	0.000	0.990	0.000	1.000	NA	0.906	LOF
**86**	1	15	1863	173	0.839	0.842	0.158	0.161	0.006	0.992	0.063	0.938	0.011	0.908	LOF
**87**	1	18	1860	173	0.842	0.845	0.155	0.158	0.006	0.990	0.053	0.947	0.010	0.907	LOF
**88**	0	16	1862	174	0.845	0.851	0.149	0.155	0.000	0.991	0.000	1.000	NA	0.907	LOF
**89**	3	22	1856	171	0.851	0.856	0.144	0.149	0.017	0.988	0.120	0.880	0.030	0.906	LOF
**90**	0	19	1859	174	0.856	0.861	0.139	0.144	0.000	0.990	0.000	1.000	NA	0.906	LOF
**91**	0	13	1865	174	0.861	0.865	0.135	0.139	0.000	0.993	0.000	1.000	NA	0.909	LOF
**92**	0	20	1858	174	0.865	0.870	0.130	0.135	0.000	0.989	0.000	1.000	NA	0.905	LOF
**93**	1	18	1860	173	0.870	0.877	0.123	0.130	0.006	0.990	0.053	0.947	0.010	0.907	LOF
**94**	1	17	1861	173	0.877	0.882	0.118	0.123	0.006	0.991	0.056	0.944	0.010	0.907	LOF
**95**	0	23	1855	174	0.882	0.889	0.111	0.118	0.000	0.988	0.000	1.000	NA	0.904	LOF

**96**	0	17	1861	174	0.889	0.896	0.104	0.111	0.000	0.991	0.000	1.000	NA	0.907	LOF
**97**	1	11	1867	173	0.897	0.907	0.093	0.103	0.006	0.994	0.083	0.917	0.011	0.910	LOF
**98**	4	11	1867	170	0.907	0.920	0.080	0.093	0.023	0.994	0.267	0.733	0.042	0.912	LOF
**99**	49	7	1871	125	0.920	0.928	0.072	0.080	0.282	0.996	0.875	0.125	0.426	0.936	LOF
**100**	8	12	1866	166	0.928	0.972	0.028	0.072	0.046	0.994	0.400	0.600	0.082	0.913	LOF

Supplementary Table 8. Score percentiles of the model without clinical features across our training dataset (n = 174) and population variants (n = 13,389). For each percentile, the score thresholds (pGOF, pLOF) are shown along with their discrimination of training dataset versus population variants as accuracy, false discovery rate (FDR) and F1 score. Note that score percentiles were calculated separately for each class (GOF/LOF).

Subset	Score	LR	LR (lower bound)	LR (upper bound)	P_{in}	P_{out}	B_{in}	B_{out}	ACMG evidence
pGOF	0.0585	7.33	3.9	31.3	44	178	4	144	PP3_supporting
pGOF	0.0599	5.87	2.96	15.7	44	178	5	143	PP3_supporting
pGOF	0.0613	5.87	2.96	15.7	44	178	5	143	PP3_supporting
pGOF	0.0673	6.67	3.41	17.7	50	172	5	143	PP3_supporting
pGOF	0.0706	5.87	2.96	15.7	44	178	5	143	PP3_supporting
pGOF	0.0723	5.87	2.96	15.7	44	178	5	143	PP3_supporting
pGOF	0.0724	5.87	2.96	15.7	44	178	5	143	PP3_supporting
pGOF	0.0728	5.87	2.96	15.7	44	178	5	143	PP3_supporting
pGOF	0.0753	6.27	3.19	16.7	47	175	5	143	PP3_supporting
pGOF	0.0768	6.53	3.33	17.3	49	173	5	143	PP3_supporting
pGOF	0.0826	5.87	2.96	15.7	44	178	5	143	PP3_supporting
pGOF	0.0832	5.87	2.96	15.7	44	178	5	143	PP3_supporting
pGOF	0.0841	6.13	3.11	16.3	46	176	5	143	PP3_supporting
pGOF	0.0844	6.13	3.11	16.3	46	176	5	143	PP3_supporting
pGOF	0.0859	6.27	3.19	16.7	47	175	5	143	PP3_supporting
pGOF	0.0877	6.8	3.48	18	51	171	5	143	PP3_supporting
pGOF	0.0915	5.87	2.96	15.7	44	178	5	143	PP3_supporting
pGOF	0.0918	5.87	2.96	15.7	44	178	5	143	PP3_supporting
pGOF	0.0969	6.53	3.33	17.3	49	173	5	143	PP3_supporting
pGOF	0.0996	5.73	2.89	15.3	43	179	5	143	PP3_supporting
pGOF	0.0998	5.73	2.89	15.3	43	179	5	143	PP3_supporting
pGOF	0.1	5.6	2.81	15	42	180	5	143	PP3_supporting
pGOF	0.101	5.6	2.81	15	42	180	5	143	PP3_supporting
pGOF	0.104	5.73	2.89	15.3	43	179	5	143	PP3_supporting
pGOF	0.105	5.87	2.96	15.7	44	178	5	143	PP3_supporting
pGOF	0.108	4	2	18	24	198	4	144	No_computational_evidence
pGOF	0.11	3.2	1.75	9	24	198	5	143	No_computational_evidence

pGOF	0.121	1.47	0.667	4.67	11	211	5	143	No_computational_evidence
pGOF	0.122	1.47	0.667	4.67	11	211	5	143	No_computational_evidence
pGOF	0.124	1.73	0.833	5.33	13	209	5	143	No_computational_evidence
pGOF	0.126	1.6	0.75	5	12	210	5	143	No_computational_evidence
pGOF	0.128	1	0.286	2.67	6	216	4	144	No_computational_evidence
pGOF	0.129	1	0.286	2.67	6	216	4	144	No_computational_evidence
pGOF	0.132	1.73	0.833	5.33	13	209	5	143	No_computational_evidence
pGOF	0.138	1.5	0.571	8	9	213	4	144	No_computational_evidence
pGOF	0.139	1.78	0.8	6.67	8	214	3	145	No_computational_evidence
pGOF	0.144	1.78	0.8	6.67	8	214	3	145	No_computational_evidence
pGOF	0.144	1.78	0.556	6.67	8	214	3	145	No_computational_evidence
pGOF	0.145	1.78	0.8	6.67	8	214	3	145	No_computational_evidence
pGOF	0.146	1.33	0.476	7.33	8	214	4	144	No_computational_evidence
pGOF	0.147	1.33	0.476	7.33	8	214	4	144	No_computational_evidence
pGOF	0.147	1.07	0.417	3.67	8	214	5	143	No_computational_evidence
pGOF	0.153	2.17	0.952	10.7	13	209	4	144	No_computational_evidence
pGOF	0.155	1.67	0.667	8.67	10	212	4	144	No_computational_evidence
pGOF	0.157	1.33	0.476	7.33	8	214	4	144	No_computational_evidence
pGOF	0.158	1	0.286	2.67	6	216	4	144	No_computational_evidence
pGOF	0.159	1.17	0.381	6.67	7	215	4	144	No_computational_evidence
pGOF	0.161	0.889	0.37	2.44	8	214	6	142	No_computational_evidence
pGOF	0.162	0.762	0.333	1.83	8	214	7	141	No_computational_evidence
pGOF	0.163	0.952	0.467	2.17	10	212	7	141	No_computational_evidence
pGOF	0.169	1.47	0.667	4.67	11	211	5	143	No_computational_evidence
pGOF	0.169	1.2	0.5	4	9	213	5	143	No_computational_evidence
pGOF	0.17	1	0.444	2.67	9	213	6	142	No_computational_evidence
pGOF	0.171	1	0.444	2.67	9	213	6	142	No_computational_evidence
pGOF	0.171	1.07	0.417	3.67	8	214	5	143	No_computational_evidence

pGOF	0.172	0.933	0.333	3.33	7	215	5	143	No_computational_evidence
pGOF	0.173	0.933	0.333	3.33	7	215	5	143	No_computational_evidence
pGOF	0.173	1.17	0.381	6.67	7	215	4	144	No_computational_evidence
pGOF	0.176	1.07	0.417	3.67	8	214	5	143	No_computational_evidence
pGOF	0.18	0.933	0.333	3.33	7	215	5	143	No_computational_evidence
pGOF	0.183	1.33	0.606	2.83	14	208	7	141	No_computational_evidence
pGOF	0.184	1.33	0.606	2.83	14	208	7	141	No_computational_evidence
pGOF	0.186	1.33	0.606	2.83	14	208	7	141	No_computational_evidence
pGOF	0.19	1.56	0.667	6	7	215	3	145	No_computational_evidence
pGOF	0.192	1.56	0.667	6	7	215	3	145	No_computational_evidence
pGOF	0.194	0.727	0.356	1.52	12	210	11	137	No_computational_evidence
pGOF	0.195	0.444	0.231	0.792	14	208	21	127	No_computational_evidence
pGOF	0.199	0.456	0.303	0.667	26	196	38	110	No_computational_evidence
pGOF	0.2	0.423	0.264	0.647	26	196	41	107	No_computational_evidence
pGOF	0.209	0.412	0.25	0.643	21	201	34	114	No_computational_evidence
pGOF	0.21	0.333	0.197	0.521	19	203	38	110	No_computational_evidence
pGOF	0.211	0.333	0.197	0.521	19	203	38	110	No_computational_evidence
pGOF	0.212	0.325	0.193	0.505	19	203	39	109	No_computational_evidence
pGOF	0.212	0.317	0.188	0.49	19	203	40	108	No_computational_evidence
pGOF	0.213	0.309	0.184	0.476	19	203	41	107	No_computational_evidence
pGOF	0.213	0.302	0.181	0.463	19	203	42	106	No_computational_evidence
pGOF	0.214	0.27	0.164	0.407	19	203	47	101	BP4_supporting
pGOF	0.214	0.259	0.158	0.388	19	203	49	99	BP4_supporting
pGOF	0.215	0.259	0.158	0.388	19	203	49	99	BP4_supporting
pGOF	0.216	0.259	0.158	0.388	19	203	49	99	BP4_supporting
pGOF	0.217	0.259	0.158	0.388	19	203	49	99	BP4_supporting
pGOF	0.217	0.267	0.167	0.394	20	202	50	98	BP4_supporting
pGOF	0.217	0.295	0.195	0.444	23	199	52	96	No_computational_evidence

pGOF	0.218	0.295	0.181	0.444	23	199	52	96	No_computational_evidence
pGOF	0.218	0.295	0.195	0.444	23	199	52	96	No_computational_evidence
pGOF	0.219	0.295	0.195	0.444	23	199	52	96	No_computational_evidence
pGOF	0.219	0.284	0.189	0.426	23	199	54	94	No_computational_evidence
pGOF	0.219	0.284	0.175	0.426	23	199	54	94	No_computational_evidence
pGOF	0.22	0.289	0.192	0.435	23	199	53	95	No_computational_evidence
pGOF	0.222	0.288	0.187	0.415	22	200	51	97	BP4_supporting
pGOF	0.222	0.293	0.19	0.45	22	200	50	98	No_computational_evidence
pGOF	0.222	0.286	0.182	0.444	21	201	49	99	No_computational_evidence
pGOF	0.223	0.272	0.17	0.403	20	202	49	99	BP4_supporting
pGOF	0.224	0.272	0.17	0.403	20	202	49	99	BP4_supporting
pGOF	0.224	0.275	0.167	0.417	19	203	46	102	No_computational_evidence
pGOF	0.227	0.252	0.143	0.43	14	208	37	111	No_computational_evidence
pGOF	0.227	0.252	0.143	0.396	14	208	37	111	BP4_supporting
pGOF	0.227	0.267	0.15	0.422	14	208	35	113	No_computational_evidence
pGOF	0.229	0.272	0.151	0.485	11	211	27	121	No_computational_evidence
pGOF	0.231	0.19	0.0833	0.392	6	216	21	127	BP4_supporting
pGOF	0.232	0.182	0.08	0.37	6	216	22	126	BP4_supporting
pGOF	0.233	0.296	0.121	0.571	8	214	18	130	No_computational_evidence
pGOF	0.237	0.545	0.222	1.24	9	213	11	137	No_computational_evidence
pGOF	0.238	0.37	0.111	0.889	5	217	9	139	No_computational_evidence
pGOF	0.238	0.333	0.103	0.762	5	217	10	138	No_computational_evidence
pGOF	0.239	0.417	0.121	1.07	5	217	8	140	No_computational_evidence
pGOF	0.241	0.417	0.121	1.07	5	217	8	140	No_computational_evidence
pGOF	0.242	0.333	0.0606	0.933	4	218	8	140	No_computational_evidence
pGOF	0.243	0.333	0.0606	0.933	4	218	8	140	No_computational_evidence
pGOF	0.247	0.267	0.0513	0.667	4	218	10	138	No_computational_evidence
pGOF	0.247	0.296	0.0556	0.778	4	218	9	139	No_computational_evidence

pGOF	0.248	0.417	0.121	1.07	5	217	8	140	No_computational_evidence
pGOF	0.25	0.476	0.133	1.33	5	217	7	141	No_computational_evidence
pGOF	0.254	0.593	0.278	1.22	8	214	9	139	No_computational_evidence
pGOF	0.255	0.593	0.278	1.22	8	214	9	139	No_computational_evidence
pGOF	0.258	0.533	0.256	1.33	8	214	10	138	No_computational_evidence
pGOF	0.26	0.593	0.278	1.22	8	214	9	139	No_computational_evidence
pGOF	0.262	0.917	0.389	1.87	11	211	8	140	No_computational_evidence
pGOF	0.263	0.917	0.389	1.87	11	211	8	140	No_computational_evidence
pGOF	0.263	0.917	0.389	2.5	11	211	8	140	No_computational_evidence
pGOF	0.266	1.47	0.667	4.67	11	211	5	143	No_computational_evidence
pGOF	0.267	1.47	0.667	4.67	11	211	5	143	No_computational_evidence
pGOF	0.271	1.33	0.583	4.33	10	212	5	143	No_computational_evidence
pGOF	0.279	1.25	0.611	3.17	15	207	8	140	No_computational_evidence
pGOF	0.286	1.2	0.5	4	9	213	5	143	No_computational_evidence
pGOF	0.288	2	0.667	7.33	9	213	3	145	No_computational_evidence
pGOF	0.291	1.78	0.556	6.67	8	214	3	145	No_computational_evidence
pGOF	0.292	1.07	0.571	2.22	16	206	10	138	No_computational_evidence
pGOF	0.293	0.815	0.359	2	11	211	9	139	No_computational_evidence
pGOF	0.296	1	0.286	2.67	6	216	4	144	No_computational_evidence
pGOF	0.298	1	0.286	2.67	6	216	4	144	No_computational_evidence
pGOF	0.307	0.667	0.222	2	6	216	6	142	No_computational_evidence
pGOF	0.314	1.05	0.533	2.33	11	211	7	141	No_computational_evidence
pGOF	0.321	1.47	0.667	4.67	11	211	5	143	No_computational_evidence
pGOF	0.321	1.33	0.583	4.33	10	212	5	143	No_computational_evidence
pGOF	0.323	1.17	0.381	6.67	7	215	4	144	No_computational_evidence
pGOF	0.327	1.17	0.381	6.67	7	215	4	144	No_computational_evidence
pGOF	0.331	1	0.286	2.67	6	216	4	144	No_computational_evidence
pGOF	0.334	1	0.286	2.67	6	216	4	144	No_computational_evidence

pGOF	0.336	1	0.286	2.67	6	216	4	144	No_computational_evidence
pGOF	0.343	0.311	0.148	0.667	7	215	15	133	No_computational_evidence
pGOF	0.354	0.314	0.127	0.615	8	214	17	131	No_computational_evidence
pGOF	0.356	0.19	0.0392	0.424	4	218	14	134	No_computational_evidence
pGOF	0.358	0.157	0.0333	0.333	4	218	17	131	BP4_supporting
pGOF	0.359	0.157	0.0333	0.333	4	218	17	131	BP4_supporting
pGOF	0.36	0.157	0.0333	0.333	4	218	17	131	BP4_supporting
pGOF	0.361	0.157	0.0333	0.333	4	218	17	131	BP4_supporting
pGOF	0.364	0.208	0.0702	0.41	5	217	16	132	BP4_supporting
pGOF	0.365	0.208	0.0702	0.41	5	217	16	132	BP4_supporting
pGOF	0.366	0.25	0.105	0.556	6	216	16	132	No_computational_evidence
pGOF	0.366	0.267	0.111	0.606	6	216	15	133	No_computational_evidence
pGOF	0.367	0.267	0.111	0.606	6	216	15	133	No_computational_evidence
pGOF	0.374	0.8	0.25	3	6	216	5	143	No_computational_evidence
pGOF	0.375	0.471	0.254	0.821	12	210	17	131	No_computational_evidence
pGOF	0.379	0.533	0.281	0.97	12	210	15	133	No_computational_evidence
pGOF	0.379	0.489	0.246	0.909	11	211	15	133	No_computational_evidence
pGOF	0.381	1.05	0.533	2.33	11	211	7	141	No_computational_evidence
pGOF	0.389	0.75	0.364	1.6	9	213	8	140	No_computational_evidence
pGOF	0.393	0.247	0.129	0.455	10	212	27	121	No_computational_evidence
pGOF	0.394	0.214	0.104	0.361	9	213	28	120	BP4_supporting
pGOF	0.401	0.333	0.0606	0.933	4	218	8	140	No_computational_evidence
pGOF	0.404	0.148	0.0667	0.29	6	216	27	121	BP4_supporting
pGOF	0.406	0.173	0.0645	0.319	7	215	27	121	BP4_supporting
pGOF	0.407	0.173	0.0645	0.319	7	215	27	121	BP4_supporting
pGOF	0.408	0.173	0.0645	0.319	7	215	27	121	BP4_supporting
pGOF	0.409	0.173	0.0645	0.319	7	215	27	121	BP4_supporting
pGOF	0.41	0.148	0.0667	0.29	6	216	27	121	BP4_supporting

pGOF	0.411	0.148	0.0667	0.29	6	216	27	121	BP4_supporting
pGOF	0.412	0.148	0.0667	0.29	6	216	27	121	BP4_supporting
pGOF	0.414	0.123	0.0444	0.222	5	217	27	121	BP4_supporting
pGOF	0.425	0.16	0.0714	0.317	6	216	25	123	BP4_supporting
pGOF	0.437	0.179	0.0667	0.333	7	215	26	122	BP4_supporting
pGOF	0.441	0.2	0.087	0.417	6	216	20	128	No_computational_evidence
pGOF	0.445	0.198	0.086	0.348	8	214	27	121	BP4_supporting
pGOF	0.456	0.222	0.108	0.377	9	213	27	121	BP4_supporting
pGOF	0.46	0.317	0.16	0.549	10	212	21	127	No_computational_evidence
pGOF	0.479	0.515	0.296	0.863	17	205	22	126	No_computational_evidence
pGOF	0.495	3.33	1.33	11.3	15	207	3	145	No_computational_evidence
pGOF	0.509	2.17	0.952	10.7	13	209	4	144	No_computational_evidence
pGOF	0.512	2.17	0.952	10.7	13	209	4	144	No_computational_evidence
pGOF	0.513	2.17	0.952	10.7	13	209	4	144	No_computational_evidence
pGOF	0.524	2.33	1.05	11.3	14	208	4	144	No_computational_evidence
pGOF	0.526	2.5	1.14	12	15	207	4	144	No_computational_evidence
pGOF	0.528	2.5	1.14	12	15	207	4	144	No_computational_evidence
pGOF	0.53	2.5	1.14	12	15	207	4	144	No_computational_evidence
pGOF	0.539	2.83	1.33	13.3	17	205	4	144	No_computational_evidence
pGOF	0.552	2.4	1.25	7	18	204	5	143	No_computational_evidence
pGOF	0.562	3.78	1.56	12.7	17	205	3	145	No_computational_evidence
pGOF	0.568	3.56	1.44	12	16	206	3	145	No_computational_evidence
pGOF	0.576	3.33	1.33	11.3	15	207	3	145	No_computational_evidence
pGOF	0.598	1.78	0.556	6.67	8	214	3	145	No_computational_evidence
pGOF	0.613	2.22	0.778	8	10	212	3	145	No_computational_evidence
pGOF	0.623	2.22	0.778	8	10	212	3	145	No_computational_evidence
pGOF	0.638	2.22	0.778	8	10	212	3	145	No_computational_evidence
pGOF	0.645	2.22	0.778	8	10	212	3	145	No_computational_evidence

pGOF	0.657	3.78	1.56	12.7	17	205	3	145	No_computational_evidence
pGOF	0.659	4.22	1.78	14	19	203	3	145	No_computational_evidence
pGOF	0.677	4.44	1.89	14.7	20	202	3	145	No_computational_evidence
pGOF	0.69	6.22	2.78	20	28	194	3	145	PP3_supporting
pGOF	0.698	4.5	2.29	20	27	195	4	144	No_computational_evidence
pGOF	0.732	5.11	2.22	16.7	23	199	3	145	No_computational_evidence
pGOF	0.737	5.11	2.22	16.7	23	199	3	145	No_computational_evidence
pGOF	0.739	4.89	2.11	16	22	200	3	145	No_computational_evidence
pGOF	0.741	4.89	2.11	16	22	200	3	145	No_computational_evidence
pGOF	0.742	4.89	2.11	16	22	200	3	145	No_computational_evidence
pGOF	0.743	4.89	2.11	16	22	200	3	145	No_computational_evidence
pGOF	0.746	5.56	2.44	18	25	197	3	145	PP3_supporting
pGOF	0.758	5	2.57	22	30	192	4	144	PP3_supporting
pGOF	0.777	5.56	2.44	18	25	197	3	145	PP3_supporting
pGOF	0.784	6	2.67	19.3	27	195	3	145	PP3_supporting
pGOF	0.787	6	2.67	19.3	27	195	3	145	PP3_supporting
pGOF	0.789	6	2.67	19.3	27	195	3	145	PP3_supporting
pGOF	0.798	5.17	2.67	22.7	31	191	4	144	PP3_supporting
pGOF	0.803	7.56	3.44	24	34	188	3	145	PP3_supporting
pGOF	0.804	7.56	3.44	24	34	188	3	145	PP3_supporting
pGOF	0.815	7.56	3.44	24	34	188	3	145	PP3_supporting
pGOF	0.816	6.44	2.89	20.7	29	193	3	145	PP3_supporting
pGOF	0.827	4.89	2.11	16	22	200	3	145	No_computational_evidence
pGOF	0.852	4.67	2	15.3	21	201	3	145	No_computational_evidence
pGOF	0.856	5.11	2.22	16.7	23	199	3	145	No_computational_evidence
pGOF	0.859	3.33	1.33	11.3	15	207	3	145	No_computational_evidence
pGOF	0.862	4	1.67	13.3	18	204	3	145	No_computational_evidence
pGOF	0.867	4	1.67	13.3	18	204	3	145	No_computational_evidence

pGOF	0.868	4.22	1.78	14	19	203	3	145	No_computational_evidence
pGOF	0.872	4.89	2.11	16	22	200	3	145	No_computational_evidence
pGOF	0.875	5.11	2.22	16.7	23	199	3	145	No_computational_evidence
pGOF	0.879	8.89	4.11	28	40	182	3	145	PP3_supporting
pGOF	0.882	9.11	4.22	28.7	41	181	3	145	PP3_supporting
pGOF	0.889	9.33	4.33	29.3	42	180	3	145	PP3_supporting
pGOF	0.892	9.33	4.33	29.3	42	180	3	145	PP3_supporting
pGOF	0.895	9.33	4.33	29.3	42	180	3	145	PP3_supporting
pGOF	0.906	9.33	4.33	29.3	42	180	3	145	PP3_supporting
pGOF	0.908	9.33	4.33	29.3	42	180	3	145	PP3_supporting
pGOF	0.91	9.33	4.33	29.3	42	180	3	145	PP3_supporting
pGOF	0.911	9.33	4.33	29.3	42	180	3	145	PP3_supporting
pGOF	0.914	9.33	4.33	29.3	42	180	3	145	PP3_supporting
pGOF	0.915	9.33	4.33	29.3	42	180	3	145	PP3_supporting
pGOF	0.918	9.33	4.33	29.3	42	180	3	145	PP3_supporting
pGOF	0.92	9.33	4.33	29.3	42	180	3	145	PP3_supporting
pGOF	0.922	9.33	4.33	29.3	42	180	3	145	PP3_supporting
pGOF	0.928	9.33	4.33	29.3	42	180	3	145	PP3_supporting
pGOF	0.929	9.33	4.33	29.3	42	180	3	145	PP3_supporting
pGOF	0.93	9.33	4.33	29.3	42	180	3	145	PP3_supporting
pGOF	0.935	9.33	4.33	29.3	42	180	3	145	PP3_supporting
pGOF	0.939	9.33	4.33	29.3	42	180	3	145	PP3_supporting
pGOF	0.943	9.33	4.33	29.3	42	180	3	145	PP3_supporting
pLOF	0.0572	9.33	4.33	29.3	42	180	3	145	PP3_supporting
pLOF	0.0605	9.33	4.33	29.3	42	180	3	145	PP3_supporting
pLOF	0.065	9.33	4.33	29.3	42	180	3	145	PP3_supporting
pLOF	0.0698	9.33	4.33	29.3	42	180	3	145	PP3_supporting
pLOF	0.0715	9.33	4.33	29.3	42	180	3	145	PP3_supporting

pLOF	0.0716	9.33	4.33	29.3	42	180	3	145	PP3_supporting
pLOF	0.0718	9.33	4.33	29.3	42	180	3	145	PP3_supporting
pLOF	0.0784	9.33	4.33	29.3	42	180	3	145	PP3_supporting
pLOF	0.08	9.33	4.33	29.3	42	180	3	145	PP3_supporting
pLOF	0.0803	9.33	4.33	29.3	42	180	3	145	PP3_supporting
pLOF	0.0817	9.33	4.33	29.3	42	180	3	145	PP3_supporting
pLOF	0.0824	9.33	4.33	29.3	42	180	3	145	PP3_supporting
pLOF	0.0847	9.33	4.33	29.3	42	180	3	145	PP3_supporting
pLOF	0.0857	9.33	4.33	29.3	42	180	3	145	PP3_supporting
pLOF	0.0888	9.33	4.33	29.3	42	180	3	145	PP3_supporting
pLOF	0.0891	9.33	4.33	29.3	42	180	3	145	PP3_supporting
pLOF	0.0902	9.33	4.33	29.3	42	180	3	145	PP3_supporting
pLOF	0.0923	9.33	4.33	29.3	42	180	3	145	PP3_supporting
pLOF	0.0939	9.33	4.33	29.3	42	180	3	145	PP3_supporting
pLOF	0.105	9.33	4.33	29.3	42	180	3	145	PP3_supporting
pLOF	0.108	9.33	4.33	29.3	42	180	3	145	PP3_supporting
pLOF	0.111	9.33	4.33	29.3	42	180	3	145	PP3_supporting
pLOF	0.118	9.11	4.22	28.7	41	181	3	145	PP3_supporting
pLOF	0.121	8.89	4.11	28	40	182	3	145	PP3_supporting
pLOF	0.125	5.11	2.22	16.7	23	199	3	145	No_computational_evidence
pLOF	0.128	4.89	2.11	16	22	200	3	145	No_computational_evidence
pLOF	0.132	4.22	1.78	14	19	203	3	145	No_computational_evidence
pLOF	0.133	4	1.67	13.3	18	204	3	145	No_computational_evidence
pLOF	0.138	4	1.67	13.3	18	204	3	145	No_computational_evidence
pLOF	0.141	3.33	1.33	11.3	15	207	3	145	No_computational_evidence
pLOF	0.144	5.11	2.22	16.7	23	199	3	145	No_computational_evidence
pLOF	0.148	4.67	2	15.3	21	201	3	145	No_computational_evidence
pLOF	0.173	4.89	2.11	16	22	200	3	145	No_computational_evidence

pLOF	0.184	6.44	2.89	20.7	29	193	3	145	PP3_supporting
pLOF	0.185	7.56	3.44	24	34	188	3	145	PP3_supporting
pLOF	0.196	7.56	3.44	24	34	188	3	145	PP3_supporting
pLOF	0.197	7.56	3.44	24	34	188	3	145	PP3_supporting
pLOF	0.202	5.17	2.67	22.7	31	191	4	144	PP3_supporting
pLOF	0.211	6	2.67	19.3	27	195	3	145	PP3_supporting
pLOF	0.213	6	2.67	19.3	27	195	3	145	PP3_supporting
pLOF	0.216	6	2.67	19.3	27	195	3	145	PP3_supporting
pLOF	0.223	5.56	2.44	18	25	197	3	145	PP3_supporting
pLOF	0.242	5	2.57	22	30	192	4	144	PP3_supporting
pLOF	0.254	5.56	2.44	18	25	197	3	145	PP3_supporting
pLOF	0.257	4.89	2.11	16	22	200	3	145	No_computational_evidence
pLOF	0.258	4.89	2.11	16	22	200	3	145	No_computational_evidence
pLOF	0.259	4.89	2.11	16	22	200	3	145	No_computational_evidence
pLOF	0.261	4.89	2.11	16	22	200	3	145	No_computational_evidence
pLOF	0.263	5.11	2.22	16.7	23	199	3	145	No_computational_evidence
pLOF	0.268	5.11	2.22	16.7	23	199	3	145	No_computational_evidence
pLOF	0.302	4.5	2.29	20	27	195	4	144	No_computational_evidence
pLOF	0.31	6.22	2.78	20	28	194	3	145	PP3_supporting
pLOF	0.323	4.44	1.89	14.7	20	202	3	145	No_computational_evidence
pLOF	0.341	4.22	1.78	14	19	203	3	145	No_computational_evidence
pLOF	0.343	3.78	1.56	12.7	17	205	3	145	No_computational_evidence
pLOF	0.355	2.22	0.778	8	10	212	3	145	No_computational_evidence
pLOF	0.362	2.22	0.778	8	10	212	3	145	No_computational_evidence
pLOF	0.377	2.22	0.778	8	10	212	3	145	No_computational_evidence
pLOF	0.387	2.22	0.778	8	10	212	3	145	No_computational_evidence
pLOF	0.402	1.78	0.556	6.67	8	214	3	145	No_computational_evidence
pLOF	0.424	3.33	1.33	11.3	15	207	3	145	No_computational_evidence

pLOF	0.432	3.56	1.44	12	16	206	3	145	No_computational_evidence
pLOF	0.438	3.78	1.56	12.7	17	205	3	145	No_computational_evidence
pLOF	0.448	2.4	1.25	7	18	204	5	143	No_computational_evidence
pLOF	0.461	2.83	1.33	13.3	17	205	4	144	No_computational_evidence
pLOF	0.47	2.5	1.14	12	15	207	4	144	No_computational_evidence
pLOF	0.472	2.5	1.14	12	15	207	4	144	No_computational_evidence
pLOF	0.474	2.5	1.14	12	15	207	4	144	No_computational_evidence
pLOF	0.476	2.33	1.05	11.3	14	208	4	144	No_computational_evidence
pLOF	0.487	2.17	0.952	10.7	13	209	4	144	No_computational_evidence
pLOF	0.488	2.17	0.952	10.7	13	209	4	144	No_computational_evidence
pLOF	0.491	2.17	0.952	10.7	13	209	4	144	No_computational_evidence
pLOF	0.505	3.33	1.33	11.3	15	207	3	145	No_computational_evidence
pLOF	0.521	0.515	0.296	0.863	17	205	22	126	No_computational_evidence
pLOF	0.54	0.317	0.16	0.549	10	212	21	127	No_computational_evidence
pLOF	0.544	0.222	0.108	0.377	9	213	27	121	BP4_supporting
pLOF	0.555	0.198	0.086	0.348	8	214	27	121	BP4_supporting
pLOF	0.559	0.2	0.087	0.417	6	216	20	128	No_computational_evidence
pLOF	0.563	0.179	0.0667	0.333	7	215	26	122	BP4_supporting
pLOF	0.575	0.16	0.0714	0.317	6	216	25	123	BP4_supporting
pLOF	0.586	0.123	0.0444	0.261	5	217	27	121	BP4_supporting
pLOF	0.588	0.148	0.0667	0.29	6	216	27	121	BP4_supporting
pLOF	0.589	0.148	0.0667	0.29	6	216	27	121	BP4_supporting
pLOF	0.59	0.148	0.0667	0.29	6	216	27	121	BP4_supporting
pLOF	0.591	0.173	0.0645	0.319	7	215	27	121	BP4_supporting
pLOF	0.592	0.173	0.0645	0.319	7	215	27	121	BP4_supporting
pLOF	0.593	0.173	0.0645	0.319	7	215	27	121	BP4_supporting
pLOF	0.594	0.173	0.0645	0.319	7	215	27	121	BP4_supporting
pLOF	0.596	0.148	0.0667	0.29	6	216	27	121	BP4_supporting

pLOF	0.599	0.333	0.0606	0.933	4	218	8	140	No_computational_evidence
pLOF	0.606	0.214	0.104	0.361	9	213	28	120	BP4_supporting
pLOF	0.607	0.247	0.129	0.406	10	212	27	121	BP4_supporting
pLOF	0.611	0.75	0.364	1.6	9	213	8	140	No_computational_evidence
pLOF	0.619	1.05	0.533	2.33	11	211	7	141	No_computational_evidence
pLOF	0.621	0.489	0.246	0.909	11	211	15	133	No_computational_evidence
pLOF	0.621	0.533	0.281	0.97	12	210	15	133	No_computational_evidence
pLOF	0.625	0.471	0.254	0.821	12	210	17	131	No_computational_evidence
pLOF	0.626	0.8	0.25	3	6	216	5	143	No_computational_evidence
pLOF	0.633	0.267	0.111	0.5	6	216	15	133	No_computational_evidence
pLOF	0.633	0.267	0.111	0.606	6	216	15	133	No_computational_evidence
pLOF	0.634	0.267	0.111	0.5	6	216	15	133	No_computational_evidence
pLOF	0.634	0.25	0.105	0.556	6	216	16	132	No_computational_evidence
pLOF	0.635	0.208	0.0702	0.41	5	217	16	132	BP4_supporting
pLOF	0.636	0.208	0.0702	0.41	5	217	16	132	BP4_supporting
pLOF	0.639	0.157	0.0333	0.333	4	218	17	131	BP4_supporting
pLOF	0.64	0.157	0.0333	0.333	4	218	17	131	BP4_supporting
pLOF	0.641	0.157	0.0333	0.333	4	218	17	131	BP4_supporting
pLOF	0.642	0.157	0.0333	0.333	4	218	17	131	BP4_supporting
pLOF	0.644	0.19	0.0392	0.424	4	218	14	134	No_computational_evidence
pLOF	0.646	0.314	0.127	0.615	8	214	17	131	No_computational_evidence
pLOF	0.657	0.311	0.148	0.667	7	215	15	133	No_computational_evidence
pLOF	0.664	1	0.286	2.67	6	216	4	144	No_computational_evidence
pLOF	0.666	1	0.286	2.67	6	216	4	144	No_computational_evidence
pLOF	0.669	1	0.286	2.67	6	216	4	144	No_computational_evidence
pLOF	0.673	1.17	0.381	6.67	7	215	4	144	No_computational_evidence
pLOF	0.677	1.17	0.381	6.67	7	215	4	144	No_computational_evidence
pLOF	0.679	1.33	0.583	4.33	10	212	5	143	No_computational_evidence

pLOF	0.679	1.47	0.667	4.67	11	211	5	143	No_computational_evidence
pLOF	0.686	1.05	0.533	2.33	11	211	7	141	No_computational_evidence
pLOF	0.693	0.667	0.222	2	6	216	6	142	No_computational_evidence
pLOF	0.702	1	0.286	2.67	6	216	4	144	No_computational_evidence
pLOF	0.704	1	0.286	2.67	6	216	4	144	No_computational_evidence
pLOF	0.707	0.815	0.359	1.56	11	211	9	139	No_computational_evidence
pLOF	0.708	1.07	0.571	2.22	16	206	10	138	No_computational_evidence
pLOF	0.709	1.78	0.556	6.67	8	214	3	145	No_computational_evidence
pLOF	0.712	2	0.667	7.33	9	213	3	145	No_computational_evidence
pLOF	0.714	1.2	0.5	4	9	213	5	143	No_computational_evidence
pLOF	0.721	1.25	0.611	3.17	15	207	8	140	No_computational_evidence
pLOF	0.729	1.33	0.583	4.33	10	212	5	143	No_computational_evidence
pLOF	0.733	1.47	0.667	4.67	11	211	5	143	No_computational_evidence
pLOF	0.734	1.47	0.667	4.67	11	211	5	143	No_computational_evidence
pLOF	0.737	0.917	0.389	1.87	11	211	8	140	No_computational_evidence
pLOF	0.738	0.917	0.389	1.87	11	211	8	140	No_computational_evidence
pLOF	0.74	0.593	0.278	1.22	8	214	9	139	No_computational_evidence
pLOF	0.742	0.533	0.256	1.06	8	214	10	138	No_computational_evidence
pLOF	0.745	0.593	0.278	1.22	8	214	9	139	No_computational_evidence
pLOF	0.746	0.593	0.278	1.22	8	214	9	139	No_computational_evidence
pLOF	0.75	0.476	0.133	1.33	5	217	7	141	No_computational_evidence
pLOF	0.752	0.417	0.121	1.07	5	217	8	140	No_computational_evidence
pLOF	0.753	0.296	0.0556	0.778	4	218	9	139	No_computational_evidence
pLOF	0.753	0.267	0.0513	0.667	4	218	10	138	No_computational_evidence
pLOF	0.757	0.333	0.0606	0.933	4	218	8	140	No_computational_evidence
pLOF	0.758	0.333	0.0606	0.933	4	218	8	140	No_computational_evidence
pLOF	0.759	0.417	0.121	1.07	5	217	8	140	No_computational_evidence
pLOF	0.761	0.417	0.121	1.07	5	217	8	140	No_computational_evidence

pLOF	0.762	0.333	0.103	0.762	5	217	10	138	No_computational_evidence
pLOF	0.762	0.37	0.111	0.889	5	217	9	139	No_computational_evidence
pLOF	0.763	0.545	0.222	1.24	9	213	11	137	No_computational_evidence
pLOF	0.767	0.296	0.121	0.571	8	214	18	130	No_computational_evidence
pLOF	0.768	0.182	0.08	0.37	6	216	22	126	BP4_supporting
pLOF	0.769	0.19	0.0833	0.392	6	216	21	127	BP4_supporting
pLOF	0.771	0.272	0.151	0.485	11	211	27	121	No_computational_evidence
pLOF	0.773	0.267	0.15	0.422	14	208	35	113	No_computational_evidence
pLOF	0.773	0.252	0.143	0.396	14	208	37	111	BP4_supporting
pLOF	0.776	0.275	0.167	0.417	19	203	46	102	No_computational_evidence
pLOF	0.776	0.272	0.17	0.403	20	202	49	99	BP4_supporting
pLOF	0.777	0.272	0.17	0.403	20	202	49	99	BP4_supporting
pLOF	0.778	0.286	0.182	0.444	21	201	49	99	No_computational_evidence
pLOF	0.778	0.293	0.19	0.424	22	200	50	98	No_computational_evidence
pLOF	0.778	0.288	0.187	0.439	22	200	51	97	No_computational_evidence
pLOF	0.78	0.289	0.178	0.435	23	199	53	95	No_computational_evidence
pLOF	0.78	0.289	0.192	0.435	23	199	53	95	No_computational_evidence
pLOF	0.781	0.284	0.175	0.426	23	199	54	94	No_computational_evidence
pLOF	0.781	0.284	0.189	0.426	23	199	54	94	No_computational_evidence
pLOF	0.781	0.284	0.188	0.426	23	199	54	94	No_computational_evidence
pLOF	0.781	0.295	0.195	0.444	23	199	52	96	No_computational_evidence
pLOF	0.782	0.295	0.195	0.444	23	199	52	96	No_computational_evidence
pLOF	0.782	0.295	0.181	0.444	23	199	52	96	No_computational_evidence
pLOF	0.783	0.295	0.181	0.444	23	199	52	96	No_computational_evidence
pLOF	0.783	0.267	0.167	0.394	20	202	50	98	BP4_supporting
pLOF	0.783	0.259	0.158	0.388	19	203	49	99	BP4_supporting
pLOF	0.784	0.259	0.158	0.388	19	203	49	99	BP4_supporting
pLOF	0.785	0.259	0.158	0.388	19	203	49	99	BP4_supporting

pLOF	0.786	0.259	0.158	0.388	19	203	49	99	BP4_supporting
pLOF	0.786	0.27	0.164	0.407	19	203	47	101	BP4_supporting
pLOF	0.787	0.302	0.181	0.463	19	203	42	106	No_computational_evidence
pLOF	0.787	0.309	0.184	0.476	19	203	41	107	No_computational_evidence
pLOF	0.788	0.317	0.188	0.49	19	203	40	108	No_computational_evidence
pLOF	0.788	0.325	0.193	0.505	19	203	39	109	No_computational_evidence
pLOF	0.789	0.333	0.197	0.521	19	203	38	110	No_computational_evidence
pLOF	0.79	0.333	0.197	0.521	19	203	38	110	No_computational_evidence
pLOF	0.791	0.412	0.25	0.643	21	201	34	114	No_computational_evidence
pLOF	0.8	0.423	0.264	0.647	26	196	41	107	No_computational_evidence
pLOF	0.801	0.456	0.303	0.71	26	196	38	110	No_computational_evidence
pLOF	0.805	0.444	0.231	0.792	14	208	21	127	No_computational_evidence
pLOF	0.806	0.727	0.356	1.52	12	210	11	137	No_computational_evidence
pLOF	0.808	1.56	0.667	6	7	215	3	145	No_computational_evidence
pLOF	0.81	1.56	0.667	6	7	215	3	145	No_computational_evidence
pLOF	0.814	1.33	0.606	2.83	14	208	7	141	No_computational_evidence
pLOF	0.816	1.33	0.606	2.83	14	208	7	141	No_computational_evidence
pLOF	0.817	1.33	0.606	4	14	208	7	141	No_computational_evidence
pLOF	0.82	0.933	0.333	3.33	7	215	5	143	No_computational_evidence
pLOF	0.824	1.07	0.417	3.67	8	214	5	143	No_computational_evidence
pLOF	0.827	1.17	0.381	6.67	7	215	4	144	No_computational_evidence
pLOF	0.827	1.17	0.381	3.18	7	215	4	144	No_computational_evidence
pLOF	0.827	0.933	0.333	3.33	7	215	5	143	No_computational_evidence
pLOF	0.828	0.933	0.333	3.33	7	215	5	143	No_computational_evidence
pLOF	0.829	1.07	0.417	3.67	8	214	5	143	No_computational_evidence
pLOF	0.829	1	0.444	2.67	9	213	6	142	No_computational_evidence
pLOF	0.83	1	0.444	2.67	9	213	6	142	No_computational_evidence
pLOF	0.831	1.2	0.5	4	9	213	5	143	No_computational_evidence

pLOF	0.831	1.47	0.667	4.67	11	211	5	143	No_computational_evidence
pLOF	0.837	0.952	0.467	2.17	10	212	7	141	No_computational_evidence
pLOF	0.838	0.762	0.333	1.83	8	214	7	141	No_computational_evidence
pLOF	0.839	0.889	0.37	2.44	8	214	6	142	No_computational_evidence
pLOF	0.841	1.17	0.381	3	7	215	4	144	No_computational_evidence
pLOF	0.842	1	0.286	2.67	6	216	4	144	No_computational_evidence
pLOF	0.843	1.33	0.476	3.33	8	214	4	144	No_computational_evidence
pLOF	0.845	1.67	0.667	8.67	10	212	4	144	No_computational_evidence
pLOF	0.847	2.17	0.952	10.7	13	209	4	144	No_computational_evidence
pLOF	0.853	1.07	0.417	3.67	8	214	5	143	No_computational_evidence
pLOF	0.853	1.33	0.476	7.33	8	214	4	144	No_computational_evidence
pLOF	0.854	1.33	0.476	7.33	8	214	4	144	No_computational_evidence
pLOF	0.855	1.78	0.8	6.67	8	214	3	145	No_computational_evidence
pLOF	0.856	1.78	0.8	6.67	8	214	3	145	No_computational_evidence
pLOF	0.856	1.78	0.556	6.67	8	214	3	145	No_computational_evidence
pLOF	0.861	1.78	0.556	6.67	8	214	3	145	No_computational_evidence
pLOF	0.862	1.5	0.571	8	9	213	4	144	No_computational_evidence
pLOF	0.868	1.73	0.833	5.33	13	209	5	143	No_computational_evidence
pLOF	0.871	1	0.286	2.67	6	216	4	144	No_computational_evidence
pLOF	0.872	1	0.286	2.67	6	216	4	144	No_computational_evidence
pLOF	0.874	1.6	0.75	5	12	210	5	143	No_computational_evidence
pLOF	0.876	1.73	0.833	5.33	13	209	5	143	No_computational_evidence
pLOF	0.878	1.47	0.667	4.67	11	211	5	143	No_computational_evidence
pLOF	0.879	1.47	0.667	4.67	11	211	5	143	No_computational_evidence
pLOF	0.89	3.2	1.75	9	24	198	5	143	No_computational_evidence
pLOF	0.892	4	2	18	24	198	4	144	No_computational_evidence
pLOF	0.895	5.87	2.96	15.7	44	178	5	143	PP3_supporting
pLOF	0.896	5.73	2.89	15.3	43	179	5	143	PP3_supporting

pLOF	0.899	5.6	2.81	15	42	180	5	143	PP3_supporting
pLOF	0.9	5.6	2.81	15	42	180	5	143	PP3_supporting
pLOF	0.9	5.73	2.89	15.3	43	179	5	143	PP3_supporting
pLOF	0.903	6.53	3.33	17.3	49	173	5	143	PP3_supporting
pLOF	0.908	5.87	2.96	15.7	44	178	5	143	PP3_supporting
pLOF	0.909	5.87	2.96	15.7	44	178	5	143	PP3_supporting
pLOF	0.912	6.8	3.48	18	51	171	5	143	PP3_supporting
pLOF	0.914	6.27	3.19	16.7	47	175	5	143	PP3_supporting
pLOF	0.916	6.13	3.11	16.3	46	176	5	143	PP3_supporting
pLOF	0.917	5.87	2.96	15.7	44	178	5	143	PP3_supporting
pLOF	0.923	6.53	3.33	17.3	49	173	5	143	PP3_supporting
pLOF	0.925	6.27	3.19	16.7	47	175	5	143	PP3_supporting
pLOF	0.927	5.87	2.96	15.7	44	178	5	143	PP3_supporting
pLOF	0.928	5.87	2.96	15.7	44	178	5	143	PP3_supporting
pLOF	0.929	5.87	2.96	15.7	44	178	5	143	PP3_supporting
pLOF	0.933	6.67	3.41	17.7	50	172	5	143	PP3_supporting
pLOF	0.939	5.87	2.96	15.7	44	178	5	143	PP3_supporting
pLOF	0.94	5.87	2.96	15.7	44	178	5	143	PP3_supporting
pLOF	0.941	7.33	3.9	31.3	44	178	4	144	PP3_supporting

Supplementary Table 9. Computational evidence calibration according to the ACMG likelihood ratio-based framework. Likelihood ratio (LR) bounds are shown separately for each class probability (pGOF, pLOF).

Gene	Term	Term description	Odds Ratio	95% CI, lower bound	95% CI, upper bound	p-value	FDR-adjusted p-value
GABRB3	HP:0012639	Abnormal nervous system morphology	0.02	0.00	0.17	<0.001	<0.001
GABRB3	HP:0002011	Morphological central nervous system abnormality	0.02	0.00	0.17	<0.001	<0.001
GABRB3	HP:0012443	Abnormal brain morphology	0.02	0.00	0.17	<0.001	<0.001
GABRB3	HP:0100547	Abnormal forebrain morphology	0.02	0.00	0.17	<0.001	<0.001
GABRB3	HP:0002060	Abnormal cerebral morphology	0.02	0.00	0.17	<0.001	<0.001
GABRB3	HP:0002977	Aplasia/Hypoplasia involving the central nervous system	0.02	0.00	0.17	<0.001	<0.001
GABRB3	HP:0007364	Aplasia/Hypoplasia of the cerebrum	0.02	0.00	0.17	<0.001	<0.001
GABRB3	HP:0040195	Decreased head circumference	0.02	0.00	0.17	<0.001	<0.001
GABRB3	HP:0000252	Microcephaly	0.02	0.00	0.17	<0.001	<0.001
GABRA1	HP:0012639	Abnormal nervous system morphology	0.03	0.00	0.21	<0.001	<0.001
GABRA1	HP:0002011	Morphological central nervous system abnormality	0.03	0.00	0.21	<0.001	<0.001
GABRA1	HP:0012443	Abnormal brain morphology	0.03	0.00	0.21	<0.001	<0.001
GABRA1	HP:0100547	Abnormal forebrain morphology	0.03	0.00	0.21	<0.001	<0.001
GABRA1	HP:0002060	Abnormal cerebral morphology	0.03	0.00	0.21	<0.001	<0.001
GABRA1	HP:0002977	Aplasia/Hypoplasia involving the central nervous system	0.03	0.00	0.21	<0.001	<0.001

Gene	Term	Term description	Odds Ratio	95% CI, lower bound	95% CI, upper bound	p-value	FDR-adjusted p-value
GABRA1	HP:0007364	Aplasia/Hypoplasia of the cerebrum	0.03	0.00	0.21	<0.001	<0.001
GABRA1	HP:0040195	Decreased head circumference	0.03	0.00	0.21	<0.001	<0.001
GABRA1	HP:0000252	Microcephaly	0.03	0.00	0.21	<0.001	<0.001
GABRG2	HP:0033127	Abnormality of the musculoskeletal system	0.01	0.00	0.18	<0.001	0.002
GABRB3	HP:0100022	Abnormality of movement	0.06	0.01	0.32	<0.001	0.005
GABRB3	HP:0000152	Abnormality of head or neck	0.10	0.02	0.38	<0.001	0.005
GABRB3	HP:0000234	Abnormality of the head	0.10	0.02	0.38	<0.001	0.005
GABRB3	HP:0000924	Abnormality of the skeletal system	0.10	0.02	0.38	<0.001	0.005
GABRB3	HP:0011842	Abnormal skeletal morphology	0.10	0.02	0.38	<0.001	0.005
GABRB3	HP:0009121	Abnormal axial skeleton morphology	0.10	0.02	0.38	<0.001	0.005
GABRB3	HP:0000929	Abnormal skull morphology	0.10	0.02	0.38	<0.001	0.005
GABRB3	HP:0000240	Abnormality of skull size	0.10	0.02	0.38	<0.001	0.005
GABRB3	HP:0032892	Infection-related seizure	20.82	2.73	947.92	<0.001	0.008
GABRB3	HP:0032894	Seizure precipitated by febrile infection	20.82	2.73	947.92	<0.001	0.008
GABRG2	HP:0003011	Abnormality of the musculature	0.02	0.00	0.28	<0.001	0.008
GABRG2	HP:0011804	Abnormal muscle physiology	0.02	0.00	0.28	<0.001	0.008
GABRG2	HP:0003808	Abnormal muscle tone	0.02	0.00	0.28	<0.001	0.008
GABRG2	HP:0001252	Hypotonia	0.02	0.00	0.28	<0.001	0.008

Gene	Term	Term description	Odds Ratio	95% CI, lower bound	95% CI, upper bound	p-value	FDR-adjusted p-value
GABRB3	HP:0033127	Abnormality of the musculoskeletal system	0.12	0.03	0.44	<0.001	0.013
GABRB3	HP:0002373	Febrile seizure (within the age range of 3 months to 6 years)	18.37	2.39	838.67	0.001	0.017
GABRA1	HP:0000152	Abnormality of head or neck	0.13	0.03	0.50	0.001	0.028
GABRA1	HP:0000234	Abnormality of the head	0.13	0.03	0.50	0.001	0.028
GABRA1	HP:0000924	Abnormality of the skeletal system	0.13	0.03	0.50	0.001	0.028
GABRA1	HP:0011842	Abnormal skeletal morphology	0.13	0.03	0.50	0.001	0.028
GABRA1	HP:0009121	Abnormal axial skeleton morphology	0.13	0.03	0.50	0.001	0.028
GABRA1	HP:0000929	Abnormal skull morphology	0.13	0.03	0.50	0.001	0.028
GABRA1	HP:0000240	Abnormality of skull size	0.13	0.03	0.50	0.001	0.028
GABRA1	HP:0033127	Abnormality of the musculoskeletal system	0.18	0.05	0.58	0.002	0.043
GABRB3	HP:0005484	Secondary microcephaly	0.06	0.00	0.51	0.002	0.047
GABRB3	HP:0003011	Abnormality of the musculature	0.19	0.05	0.65	0.003	0.077
GABRB3	HP:0011804	Abnormal muscle physiology	0.19	0.05	0.65	0.003	0.077
GABRB3	HP:0003808	Abnormal muscle tone	0.19	0.05	0.65	0.003	0.077
GABRA1	HP:0001939	Abnormality of metabolism/homeostasis	5.64	1.50	26.98	0.004	0.083
GABRA1	HP:0012337	Abnormal homeostasis	5.64	1.50	26.98	0.004	0.083
GABRA1	HP:0004370	Abnormality of temperature regulation	5.64	1.50	26.98	0.004	0.083

Gene	Term	Term description	Odds Ratio	95% CI, lower bound	95% CI, upper bound	p-value	FDR-adjusted p-value
GABRA1	HP:0001945	Fever	5.64	1.50	26.98	0.004	0.083
GABRA1	HP:0032892	Infection-related seizure	13.10	1.72	596.17	0.004	0.088
GABRA1	HP:0032894	Seizure precipitated by febrile infection	13.10	1.72	596.17	0.004	0.088
GABRA1	HP:0002373	Febrile seizure (within the age range of 3 months to 6 years)	13.10	1.72	596.17	0.004	0.088
GABRB3	HP:0011451	Primary microcephaly	0.07	0.00	0.61	0.004	0.088
GABRB3	HP:0001939	Abnormality of metabolism/homeostasis	8.04	1.51	82.92	0.005	0.099
GABRB3	HP:0012337	Abnormal homeostasis	8.04	1.51	82.92	0.005	0.099
GABRB3	HP:0004370	Abnormality of temperature regulation	8.04	1.51	82.92	0.005	0.099
GABRB3	HP:0001945	Fever	8.04	1.51	82.92	0.005	0.099
GABRB2	HP:0033127	Abnormality of the musculoskeletal system	0.09	0.01	0.72	0.009	0.159
GABRB3	HP:0001332	Dystonia	0.09	0.00	0.73	0.010	0.178
GABRA1	HP:0002197	Generalized-onset seizure	10.27	1.32	471.96	0.010	0.181
GABRB3	HP:0011443	Abnormality of coordination	10.75	1.31	505.39	0.013	0.219
GABRB3	HP:0001251	Ataxia	10.75	1.31	505.39	0.013	0.219
GABRB3	HP:0007359	Focal-onset seizure	0.22	0.06	0.79	0.014	0.234
GABRA1	HP:0003011	Abnormality of the musculature	0.24	0.07	0.81	0.015	0.234
GABRA1	HP:0011804	Abnormal muscle physiology	0.24	0.07	0.81	0.015	0.234
GABRA1	HP:0003808	Abnormal muscle tone	0.24	0.07	0.81	0.015	0.234
GABRA1	HP:0011097	Epileptic spasm	0.10	0.00	0.88	0.017	0.235

Gene	Term	Term description	Odds Ratio	95% CI, lower bound	95% CI, upper bound	p-value	FDR-adjusted p-value
GABRB2	HP:0003011	Abnormality of the musculature	0.13	0.01	0.85	0.017	0.235
GABRB2	HP:0011804	Abnormal muscle physiology	0.13	0.01	0.85	0.017	0.235
GABRB2	HP:0000152	Abnormality of head or neck	0.09	0.00	0.86	0.019	0.235
GABRB2	HP:0000234	Abnormality of the head	0.09	0.00	0.86	0.019	0.235
GABRB2	HP:0000924	Abnormality of the skeletal system	0.09	0.00	0.86	0.019	0.235
GABRB2	HP:0011842	Abnormal skeletal morphology	0.09	0.00	0.86	0.019	0.235
GABRB2	HP:0009121	Abnormal axial skeleton morphology	0.09	0.00	0.86	0.019	0.235
GABRB2	HP:0000929	Abnormal skull morphology	0.09	0.00	0.86	0.019	0.235
GABRB2	HP:0000240	Abnormality of skull size	0.09	0.00	0.86	0.019	0.235
GABRB2	HP:0012639	Abnormal nervous system morphology	0.09	0.00	0.86	0.019	0.235
GABRB2	HP:0002011	Morphological central nervous system abnormality	0.09	0.00	0.86	0.019	0.235
GABRB2	HP:0012443	Abnormal brain morphology	0.09	0.00	0.86	0.019	0.235
GABRB2	HP:0100547	Abnormal forebrain morphology	0.09	0.00	0.86	0.019	0.235
GABRB2	HP:0002060	Abnormal cerebral morphology	0.09	0.00	0.86	0.019	0.235
GABRB2	HP:0002977	Aplasia/Hypoplasia involving the central nervous system	0.09	0.00	0.86	0.019	0.235
GABRB2	HP:0007364	Aplasia/Hypoplasia of the cerebrum	0.09	0.00	0.86	0.019	0.235

Gene	Term	Term description	Odds Ratio	95% CI, lower bound	95% CI, upper bound	p-value	FDR-adjusted p-value
GABRB2	HP:0040195	Decreased head circumference	0.09	0.00	0.86	0.019	0.235
GABRB2	HP:0000252	Microcephaly	0.09	0.00	0.86	0.019	0.235
GABRG2	HP:0001939	Abnormality of metabolism/homeostasis	11.03	1.12	573.65	0.020	0.235
GABRG2	HP:0012337	Abnormal homeostasis	11.03	1.12	573.65	0.020	0.235
GABRG2	HP:0004370	Abnormality of temperature regulation	11.03	1.12	573.65	0.020	0.235
GABRG2	HP:0001945	Fever	11.03	1.12	573.65	0.020	0.235
GABRB3	HP:0032792	Tonic seizure	0.10	0.00	0.91	0.022	0.256
GABRA1	HP:0001332	Dystonia	0.16	0.01	0.95	0.026	0.297
GABRB3	HP:0025766	Abnormal affect	9.14	1.08	434.29	0.027	0.297
GABRB3	HP:0025780	Abnormal volitional state	9.14	1.08	434.29	0.027	0.297
GABRB3	HP:5200263	Abnormally increased volition	9.14	1.08	434.29	0.027	0.297
GABRB3	HP:0000734	Disinhibition	9.14	1.08	434.29	0.027	0.297
GABRB3	HP:0100543	Cognitive impairment	9.05	1.08	427.64	0.028	0.297
GABRB3	HP:5200044	Reduced attention regulation	9.05	1.08	427.64	0.028	0.297
GABRB3	HP:0000736	Short attention span	9.05	1.08	427.64	0.028	0.297
GABRB3	HP:0001252	Hypotonia	0.29	0.08	1.00	0.032	0.335
GABRG2	HP:0032892	Infection-related seizure	11.96	1.14	646.43	0.033	0.344
GABRG2	HP:0032894	Seizure precipitated by febrile infection	11.96	1.14	646.43	0.033	0.344
GABRB3	HP:0011446	Abnormality of mental function	3.38	1.02	11.91	0.034	0.354

Gene	Term	Term description	Odds Ratio	95% CI, lower bound	95% CI, upper bound	p-value	FDR-adjusted p-value
GABRB3	HP:0004305	Involuntary movements	0.17	0.02	1.01	0.035	0.362
GABRG2	HP:0002373	Febrile seizure (within the age range of 3 months to 6 years)	9.13	0.86	493.09	0.042	0.422
GABRG2	HP:0002197	Generalized-onset seizure	9.13	0.86	493.09	0.042	0.422
GABRA1	HP:0020219	Motor seizure	0.34	0.11	1.07	0.045	0.446

Supplementary Table 10. Overview of 108 HPO terms with nominally significant gene-function-phenotype associations. Enrichment was obtained using Fisher's exact test, counting the number of individuals with and without the phenotype across functional groups (GOF/LOF). Positive enrichment (OR>1) signifies that a phenotype is more common in LOF variants. Multiple testing correction was done using the Benjamini-Hochberg procedure to obtain the false discovery rate (FDR) adjusted p-value.

Supplementary References

1. Brünger T, Krey I, Kim S, et al. Gene Portals: A Framework for Integrating Clinical, Functional, and Structural Evidence into Rare Disease Variant Classification. *Genetic and Genomic Medicine*. Preprint posted online March 6, 2026. doi:10.64898/2026.03.05.26347086

2. Pejaver V, Byrne AB, Feng BJ, et al. Calibration of computational tools for missense variant pathogenicity classification and ClinGen recommendations for PP3/BP4 criteria. *Am J Hum Genet*. 2022;109(12):2163-2177. doi:10.1016/j.ajhg.2022.10.013

3. Lal D, May P, Perez-Palma E, et al. Gene family information facilitates variant interpretation and identification of disease-associated genes in neurodevelopmental disorders. *Genome Med*. 2020;12(1):28. doi:10.1186/s13073-020-00725-6

4. Brünger T, Ivaniuk A, Pérez-Palma E, et al. Conserved missense variant pathogenicity and correlated phenotypes across paralogous genes. *Genome Biol*. 2025;26(1):197. doi:10.1186/s13059-025-03663-x

5. Jaganathan K, Kyriazopoulou Panagiotopoulou S, McRae JF, et al. Predicting Splicing from Primary Sequence with Deep Learning. *Cell*. 2019;176(3):535-548.e24. doi:10.1016/j.cell.2018.12.015

6. Zeng T, Li YI. Predicting RNA splicing from DNA sequence using Pangolin. *Genome Biol*. 2022;23(1):103. doi:10.1186/s13059-022-02664-4

7. de Sainte Agathe JM, Filser M, Isidor B, et al. SpliceAI-visual: a free online tool to improve SpliceAI splicing variant interpretation. *Hum Genomics*. 2023;17(1):7. doi:10.1186/s40246-023-00451-1

8. Epi25 Collaborative. Exome sequencing of 20,979 individuals with epilepsy reveals shared and distinct ultra-rare genetic risk across disorder subtypes. *Nat Neurosci*. 2024;27(10):1864-1879. doi:10.1038/s41593-024-01747-8

9. Hamosh A, Scott AF, Amberger JS, Bocchini CA, McKusick VA. Online Mendelian Inheritance in Man (OMIM), a knowledgebase of human genes and genetic disorders. *Nucleic Acids Res*. 2005;33(Database Issue):D514-D517. doi:10.1093/nar/gki033

10. Rehm HL, Berg JS, Brooks LD, et al. ClinGen — The Clinical Genome Resource. *New England Journal of Medicine*. 2015;372(23):2235-2242. doi:10.1056/NEJMsr1406261

11. Oliver KL, Scheffer IE, Bennett MF, Grinton BE, Bahlo M, Berkovic SF. Genes4Epilepsy: An epilepsy gene resource. *Epilepsia*. 2023;64(5):1368-1375. doi:10.1111/epi.17547

12. Richards S, Aziz N, Bale S, et al. Standards and Guidelines for the Interpretation of Sequence Variants: A Joint Consensus Recommendation of the American College of Medical Genetics and Genomics and the Association for Molecular Pathology. *Genet Med*. 2015;17(5):405-424. doi:10.1038/gim.2015.30

13. Ioannidis NM, Rothstein JH, Pejaver V, et al. REVEL: An Ensemble Method for Predicting the Pathogenicity of Rare Missense Variants. *Am J Hum Genet*. 2016;99(4):877-885. doi:10.1016/j.ajhg.2016.08.016

14. Millevert C, Kan ASH, Hanke M, et al. The genetic and phenotypic spectrum of GABRB1-related disorders. *Brain*. Published online June 5, 2025:awaf213. doi:10.1093/brain/awaf213
